# Mechanosensitive FHL2 tunes endothelial function via microtubule-actomyosin crosstalk

**DOI:** 10.1038/s44318-026-00807-y

**Published:** 2026-05-26

**Authors:** Shailaja Seetharaman, John Devany, Ha Ram Kim, Emma Johanna van Bodegraven, Theresa Chmiel, Shentu Tzu-Pin, Wen-hung Chou, Yun Fang, Margaret L Gardel

**Affiliations:** 1 Department of Physics, The University of Chicago, Chicago, IL USA; 2 James Franck Institute, The University of Chicago, Chicago, IL USA; 3 Institute for Biophysical Dynamics, The University of Chicago, Chicago, IL USA; 4 Mechanobiology Institute, National University of Singapore, Singapore, Singapore; 5 Department of Physiology, Yong Loo Lin School of Medicine, National University of Singapore, Singapore, Singapore; 6 Pritzker School of Molecular Engineering, The University of Chicago, Chicago, IL USA; 7 Department of Medicine, Biological Sciences Division, The University of Chicago, Chicago, IL USA; 8 Department of Translational Neuroscience, Brain Center, University Medical Center Utrecht, Utrecht University, Utrecht, The Netherlands; 9 Graduate Program in Biophysical Sciences, University of Chicago, Chicago, IL USA; 10 Molecular Genetics & Cell Biology, The University of Chicago, Chicago, IL USA; 11 Biohub, Chicago, IL USA

**Keywords:** Cell Adhesion, Polarity & Cytoskeleton, Vascular Biology & Angiogenesis

## Abstract

Endothelial tissues are essential mechanosensors in the vasculature, and defects in their response to mechanical cues such as blood flow can lead to endothelial dysfunction and cardiovascular diseases like atherosclerosis. Here, we explore how mechanoresponses tune endothelial tissue physiology and function. By bulk RNA sequencing in endothelial cells experiencing varying flow profiles, we identify a set of novel mechanosensitive genes associated with the cytoskeleton and adhesion structures. We focus on a cytoskeletal protein, Four-and-a-half LIM protein 2 (FHL2), which is consistently enriched in endothelial tissues experiencing atherosclerosis-prone disturbed flow, both in vitro and in vivo. We demonstrate that increased FHL2 expression is necessary and sufficient to induce hallmarks of atherosclerosis-like endothelial phenotypes, including aberrant cell morphology, discontinuous cell junctions, hypercontractility, and increased tissue permeability. Strikingly, this atherosclerosis-like phenotype requires the force-sensitive binding of FHL2 to the actin cytoskeleton. Mechanistically, we show that FHL2 controls endothelial tissue phenotypes by promoting RhoGTPase-dependent actomyosin contractility via release of the microtubule-bound RhoGTPase effector, GEF-H1. These findings reveal a positive mechanochemical feedback wherein FHL2 force-sensitivity tunes multi-scale mechanoresponses and endothelial tissue physiology.

## Synopsis

Defects in endothelial cell response to flow lead to tissue dysfunction and cardiovascular diseases like atherosclerosis. This study identifies FHL2 as a mechanosensitive regulator that induces atherosclerotic-like tissue phenotypes including lack of cell alignment with flow, discontinuous cell junctions, and hypercontractility.

- FHL2 is upregulated and enriched on stress fibres in endothelial tissues subjected to atherosclerosis-promoting disturbed flow.
- FHL2 increases Rho-ROCK-mediated actomyosin contractility via GEF-H1 release from microtubules.
- FHL2 expression and force-dependent localisation to actin drive junction disruption and tissue permeability.
- FHL2 reinforces positive feedback loop triggering actomyosin contractility leading to atherosclerosis-like phenotypes.

## Abstract (web summary)

Force-dependent localisation of FHL2 to stress fibres regulates actomyosin contractility and atherosclerosis-like phenotypes in endothelial cells.

## Graphical abstract

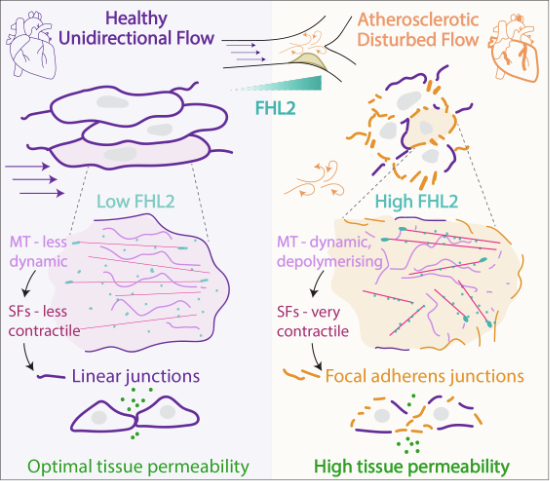

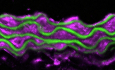

## Introduction

Endothelial cells are critical components of the vasculature that constantly sense and respond to mechanical cues, including shear stress from blood flow and stretch during vascular dilation (Davies, 1997; Davies et al, 1994). These mechanoresponses are crucial for the formation and maintenance of blood vessels during development, angiogenesis, and homeostasis (Chien, 2007; Dessalles et al, 2021). However, perturbations to mechanical cues in the vasculature can drive the onset and progression of cardiovascular diseases including atherosclerosis, stenosis, and aneurysms (Lim and Harraz, 2024).

Endothelial mechanotransduction requires specialised cell surface receptors (e.g., membrane receptors, cilia, and ion channels (Shay-Salit et al, 2002; Hierck et al, 2008; Thodeti et al, 2009; Van der Heiden et al, 2008; Polacheck et al, 2017; Mack et al, 2017)), but also mechanical contacts at adhesions to the extracellular matrix (focal adhesions) or neighbouring cells (adherens junctions) and in the cytoskeleton (Jaalouk and Lammerding, 2009; Tzima et al, 2005; Conway et al, 2013; Birukova et al, 2016). These mechanosensitive processes are crucial for determining endothelial tissue phenotypes and functions, including changes in adhesion morphology and permeability (Stroka and Aranda-Espinoza, 2011; Friedrich et al, 2019; Li et al, 2014). For instance, in healthy vasculature with laminar and unidirectional flow (UF) of blood, endothelial cells adapt to display a highly elongated and aligned morphology, and form a semi-permeable tissue barrier through stable adherens junctions (Claesson-Welsh et al, 2021; Schulte et al, 2011). However, disturbed flow (DF) of blood in arteries, typically seen at bifurcations and regions of large curvature in blood vessels, promotes the formation of atherosclerotic plaques (Zarins et al, 1983; Morbiducci et al, 2016). On the cellular scale, DF promotes drastic phenotypical remodelling of the endothelium with the loss of adherens junctions, which increases tissue permeability and inflammation (Davies et al, 2013).

**Figure 1:**
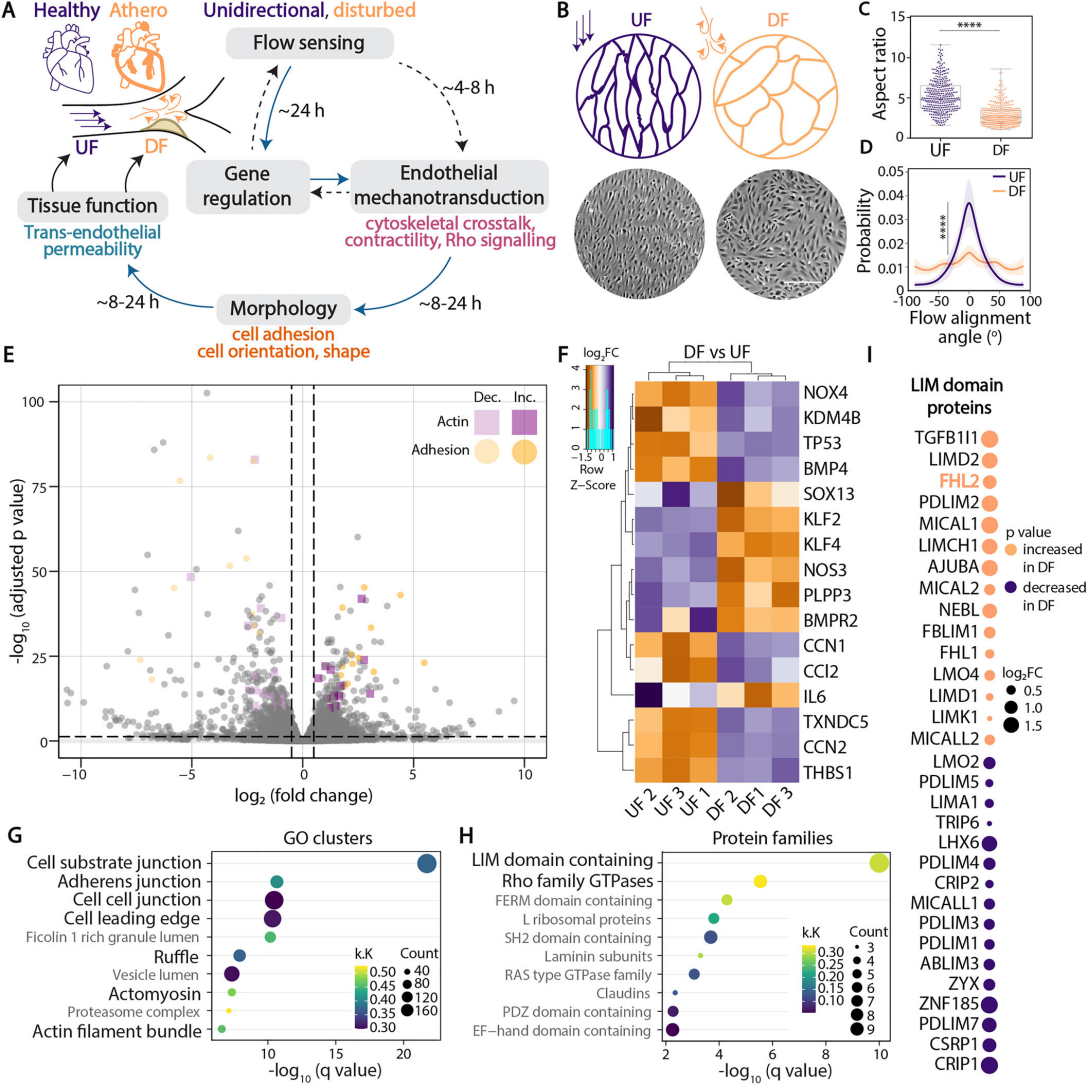
Identification of flow-sensitive gene expression profiles in endothelial cells. (**A**) Schematic showing the interplay between gene expression and endothelial mechanotransduction that facilitates multi-scale tissue adaptation, morphology, and function. In particular, hypercontractility of tissues through RhoGTPase mechano-signalling, cell-cell adhesion formation, and gene expression, collectively orchestrate tissue functions including permeability. (**B**) TeloHAECs (human aortic endothelial cells) subjected to two flow profiles: healthy laminar unidirectional flow (UF) and athero-prone disturbed flow (DF). Brightfield images of cells under UF and DF are shown. ****P < 1 × 10^−15^. (**C**) Graph shows the aspect ratio (ratio of the major to the minor axis of a cell) of cells under UF and DF. In the box-and-whisker plot, the box extends from the 25th to the 75th percentile, the whiskers show the minimum and maximum values, and the line within the box represents the median. *N* = 3 independent experiments (*n* = 361 for UF, and *n* = 425 for DF). ***P = 0.0004. (**D**) From brightfield images, the alignment of cells under UF and DF are calculated by local gradient orientation, and represented as the probability distribution of cells with the flow alignment angles (centred around 0^o^). Shaded error bars representing standard deviation are shown. *N* = 3 independent experiments (*n* = 30 for UF, and *n* = 32 for DF; fields of view, with >100 cells/field). (**E**) Volcano plot shows differentially expressed genes (DEGs) between TeloHAECs under UF and DF (dotted lines indicate log_2_FC 0.5 and -log_10_(adjusted *P* value = 0.05)). 1260/12539 genes were significantly upregulated in DF (adjusted *P* value < 0.05, log_2_FC > 0.5), and 2079/12539 genes were significantly downregulated in DF (adjusted *P* value < 0.05, log_2_FC < 0.5) (Dataset EV1). Genes annotated to adhesion-related GO terms (Dataset EV2; dark orange circles: log_2_FC >0, light orange circles: log_2_FC <0) and actin-related Gene Ontology (GO) terms (Dataset EV2; dark purple squares: log_2_FC >0, light purple squares: log_2_FC <0). (**F**) Heatmap showing Z-scored rlog transformed count data (log_2_ transformation and normalization with respect to library size) normalized of flow-sensitive genes across samples. (**G**) Top 10 most significantly enriched cellular compartment GO terms. (**H**) All DEGs which were present within adhesion- and actin-related GO terms were tested for overrepresentation in gene families. The top 10 most significantly enriched gene families are shown. (**G**, **H**) Dot size = gene set size (count = number of genes; k.K. = number of DEG genes in GO term/number of genes in the GO term. (**I**) Dotplot showing all genes significantly different between TeloHAECs under UF and DF which encode for LIM domain proteins ordered by adjusted *P* value and logFC. The size of the dots indicates absolute log_2_FC values. Four-and-a-half LIM protein 2 (FHL2) is highlighted in orange as an upregulated gene in cells under DF. For (**E**, **F**), *N* = 3 independent experiments. Statistical tests: (**C**, **D**) Unpaired Student’s *t* test followed by Mann–Whitney test (two-tailed), (**E**–**I**) *P* values using Wald’s test, multiple testing using Benjamini–Hochberg method. In the box-and-whisker plots, the box extends from the 25th to 75th percentile, the whiskers show minimum and maximum values, and the line within the box represents the median.

Endothelial tissue adaptation is driven by mechanochemical signalling; in response to flow or environmental stiffness, the RhoGTPase signalling pathway is activated, and via RhoA, there is heightened actomyosin contractility (Fig. 1A). This is often described as a feature of atherosclerotic tissues (Shimokawa et al, 2016). In turn, high contractility disrupts adherens junctions and enhances tissue permeability (Fig. 1A) (Huveneers et al, 2012). In addition to mechanochemical signalling mediating morphological phenotypes, specific transcriptional signatures are also associated with endothelial dysfunction (Fig. 1A) (Hajra et al, 2000; Tzima et al, 2001, 2002; Wójciak-Stothard et al, 2001). For instance, Krüppel-like factor *KLF2* mediates athero-protective functions in endothelial tissues, while transcription factor *NF-κB* promotes inflammatory and athero-prone states (Cybulsky and Marsden, 2014; Dabravolski et al, 2022). However, the precise mechanisms of how tissue mechanotransduction operates through rapid biochemical signals and long-term transcriptional regulation remains elusive. Thus, uncovering this multi-scale integration of endothelial mechanotransduction is critical for advancing our capabilities to precisely engineer vascular physiology. Furthermore, given these complexities, there exist very few therapies for cardiovascular diseases that directly target vascular cell dysfunction (Tamargo et al, 2023). Therefore, we sought to understand the mechanochemical feedback loops that shape the onset and progression of atherosclerotic phenotypes.

Here, we identify Four-and-a-half LIM domain protein 2 (FHL2) as a key regulator of multiscale mechanotransduction that drives the onset of athero-like endothelial phenotypes. Using RNA sequencing, we identify mechanosensitive genes in human aortic endothelial tissues subjected to flow profiles mimicking athero-protective UF and athero-prone DF. We observed differential expression of numerous adhesion- and actomyosin-related genes, with the LIM (Lin11, Isl-1 and Mec-3) domain family of proteins being the top enriched gene family within these categories. Using human aortic endothelial cells and mouse models, we find that *FHL2* is transcriptionally upregulated in DF conditions. We demonstrate that FHL2 expression is necessary and sufficient to drive hallmarks of athero-like tissue, including discontinuous adherens junctions and enhanced tissue permeability. Further, we demonstrate that FHL2 promotes actomyosin contractility through crosstalk with the microtubule cytoskeleton, and this requires force-sensitive binding of FHL2 to actin. Specifically, FHL2 alters microtubule dynamics and organisation to promote contractility via the release of a Rho guanine nucleotide exchange factor, GEF-H1, from microtubules into the cytosol, where GEF-H1 can promote RhoA signalling. Finally, subsequent FHL2-mediated hypercontractility, via reinforcement of a positive feedback loop, yields athero-like phenotypes. Broadly, our results uncover a novel mechanochemical feedback providing crucial mechanistic insight into how endothelial mechanoresponses go awry to promote atherosclerotic phenotypes.

## Results

### Transcriptomics reveals novel mechanosensitive genes in endothelial tissue response to flow

In healthy arteries resistant to atherosclerosis, blood flow is laminar and unidirectional; however, in arterial sites prone to atherosclerosis such as bifurcations, branches, and curvatures (Zarins et al, 1983; Morbiducci et al, 2016), vascular endothelial cells are constantly activated by disturbed blood flow. An example of this is the human carotid artery bifurcation, which most commonly develops atherosclerotic plaques (Wang et al, 2016; Dai et al, 2004) (Fig. 1A). Within this site, recent work has identified two arterial waveforms: “athero-protective” and “athero-prone” flows, which represent blood flow in the distal internal carotid artery and carotid sinus respectively (Fig. 1A) (Dai et al, 2004). Specifically, unidirectional flow (UF) has a high shear stress of ~2 Pa (average of 20 dyn/cm^2^, with a peak of ~40 dyn/cm^2^). This mimics the athero-protective hemodynamic flow in the distal carotid artery in humans, which ranges from 1.5 to 7 Pa. On the other hand, an oscillatory disturbed flow (DF) has a very low shear stress of ~0.2 Pa (average of 2 dyn/cm^2^, with a peak of ~10 dyn/cm^2^) and represents flows in athero-prone arterial bifurcations (Fig. 1A,B) (Dai et al, 2004; Wang et al, 2013). In other words, the DF in athero-prone regions of arteries are defined by the low time-average shear stress with rapid changes in direction (Wang et al, 2013). In this study, these hemodynamic waveforms were modelled in vitro using a motorised cone-plate system to precisely tune flow profiles and determine how healthy and athero-like phenotypes emerge in endothelial tissues in vitro (Dai et al, 2004; Krause et al, 2018; Wu et al, 2015; Chiu and Chien, 2011).

Using this model, we applied UF and DF for 24 h to a monolayer of human aortic endothelial cells (TeloHAEC; Fig. 1B). Consistent with previous studies, we observed that under UF, endothelial cells elongated and aligned in the direction of flow (Fig. 1B–D). In contrast, cell elongation and tissue alignment were lost in cells subjected to DF (Fig. 1B–D). These morphological characteristics are consistent with the phenotypes seen in vivo of the descending thoracic aorta and the aortic arch (inner curvature) that experience UF and DF, respectively (Waters et al, 2011). To describe these dysfunction endothelial morphologies, henceforth in this work, we refer to these as “athero-like” DF phenotypes.

A limited number of transcriptomic studies have been carried out in endothelial cells experiencing static, low or high shear stress (Dai et al, 2004; Ajami et al, 2017; Zaidel-Bar et al, 2005). Building on a previous study, the flow profiles used here provide a reliable comparison between two distinct conditions of healthy UF and athero-prone DF (Wu et al, 2017). Using endothelial cells under UF and DF as a robust model system, we performed bulk RNA sequencing to identify the transcriptional changes accompanying endothelial mechanoresponses to 24 h of flow. This transcriptomic screen revealed 3339 differentially expressed genes (DEGs; adjusted *P* value < 0.05), with 1260 and 2079 genes upregulated in DF and UF, respectively (Fig. 1E; Dataset EV1). Under these conditions, we depict known endothelial flow-mediated transcriptional changes, including Krüppel-like factors *KLF2* and *KLF4*, and nitric oxide synthase 3 (*NOS3*) (Fig. 1F) (Tamargo et al, 2023; Souilhol et al, 2020).

**Figure EV1:**
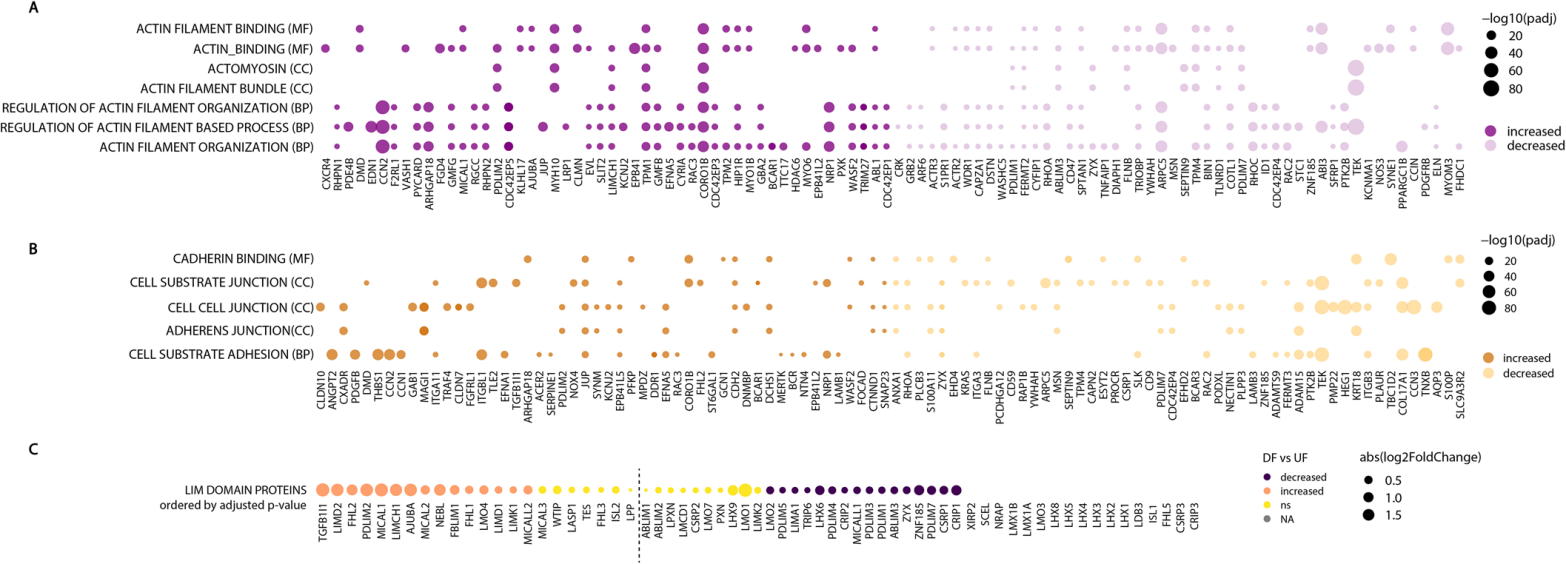
Actin- and adhesion- related differentially expressed genes in response to flow. (**A**) Actin-related genes present within the top 10 BP, CC or MF most enriched actin-related GO terms which are significantly different between endothelial cells under UF or DF are shown. The size of the dot reflects the −log_10_(adjusted *P* value). (**B**) Adhesion-related genes present within the top 10 BP, CC or MF most enriched adhesion-related GO terms which are significantly different between endothelial cells under UF or DF are shown. The size of the dot reflects the -log_10_(adjusted *P* value). (**C**) Dotplot showing all genes encoding for LIM domain proteins which were detected in endothelial cells under DF or UF. Genes are ordered by adjusted *P* value and log_2_FC. The size of the dots indicates absolute log_2_FC values. The left-most dot represents the most significantly upregulated gene in DF, and the right-most dot is the most significantly downregulated gene in DF encoding for a LIM domain protein (orange = *P*adj <0.05 & log_2_FC >0, purple = *P*adj <0.05 & log_2_FC <0, yellow = *P*adj = > 0.05). Genes of the LIM family that are not expressed in TeloHAECs are also shown.

Further, gene ontology (GO) analysis on all DEGs indicated that the most enriched gene sets in the “cellular component CC” category were related to “actin” and “adhesion” (Fig. 1E,G; Dataset EV2; actin- and adhesion-related gene sets are defined in the methods). The top DEGs belonging to these two categories are listed in Fig. EV1A,B. Amongst the DEGs annotated to the “actin” and “adhesion” terms, we observed that LIM domain-containing proteins and RhoGTPases were the top enriched gene families (Fig. 1H). In fact, we found that 31 genes in the LIM domain containing family were differentially expressed in response to different flow profiles (Figs. 1I and EV1C). As LIM domain and RhoGTPase families of proteins are crucial regulators of mechanotransduction in adherent cells (Anderson et al, 2021), we chose to focus on the role of these genes in the context of flow-induced endothelial dysfunction.

### Disturbed flow induces FHL2 endothelial expression in vitro and in vivo

The LIM domain protein family contains ~70 genes divided into 14 classes, which play critical roles in cytoskeletal organisation, adhesion, and migration, and broadly contribute to diseases including cancer. In numerous cases, the LIM domain proteins exhibit force-dependent localisation to adherens junctions, focal adhesions, and the actomyosin stress fibres, and have been implicated in mechanotransduction at these structures (Smith et al, 2010; Babu et al, 2012; Winkelman et al, 2020; Seetharaman et al, 2023; Sun et al, 2020; Sala and Oakes, 2021; Nakazawa et al, 2016; Razzell et al, 2018; Oakes et al, 2017). However, only a limited number of LIM-mediated mechanotransduction pathways are known. In addition, although some LIM domain proteins are thought to act as transcriptional co-factors (Kadrmas and MC 2004), how their transcriptional activity tunes mechanotransduction have not been explored. Thus, the functions of mechanosensitive LIM proteins and LIM-dependent downstream signalling cascades remain largely elusive, particularly in the case of cardiovascular systems. Interestingly, one of the most upregulated genes in our screen was a LIM domain protein, FHL2 (Fig. 1I). FHL2 is both highly expressed in the heart (Chan et al, 1998) and exhibits force-sensitive recruitment to actin (Sun et al, 2020). Further, studies have shown that mice with *Fhl2* deletion developed smaller atherosclerotic plaques and exhibited lower inflammation (Chen et al, 2020; Chu et al, 2010; Ebrahimian et al, 2015; Johannessen et al, 2006), suggesting a role for FHL2 in atherosclerotic disease progression.

**Figure 2:**
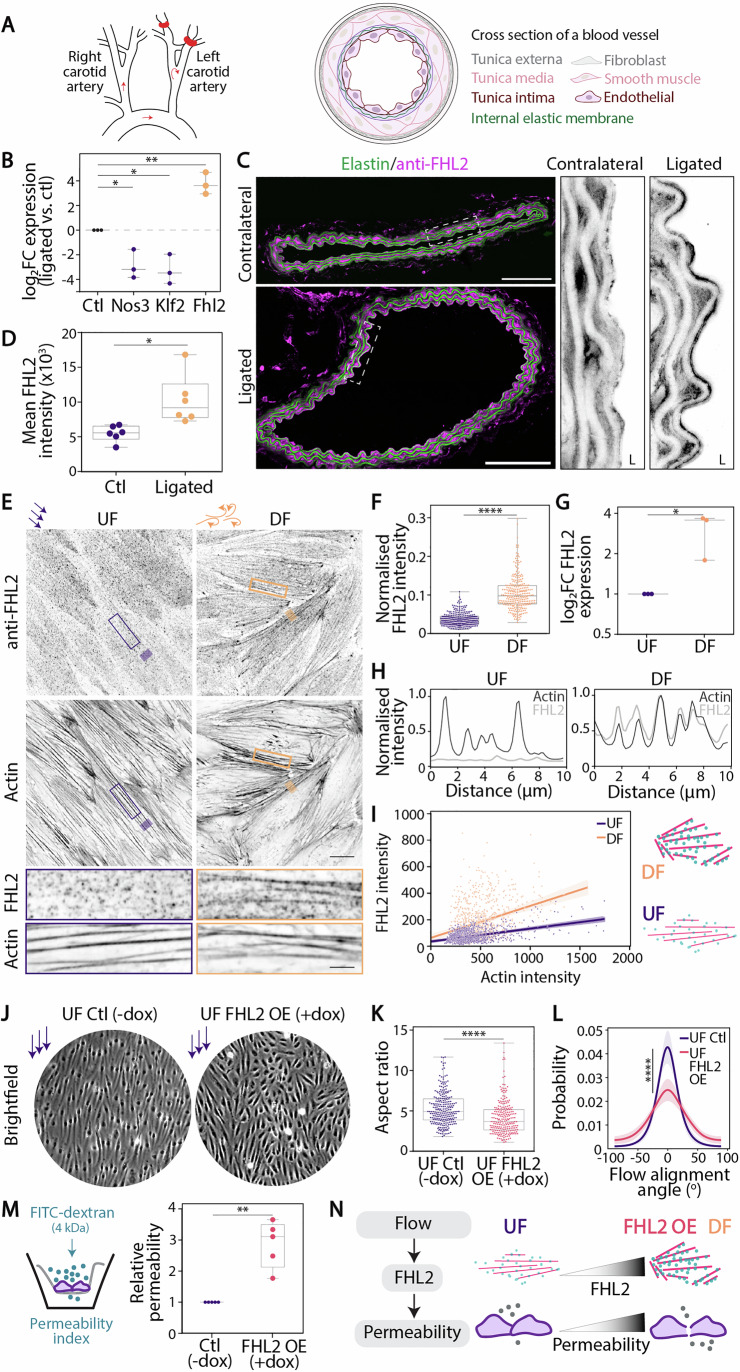
FHL2 expression and localisation to stress fibres is enriched in cells under athero-prone DF. (**A**) Partial ligation of the left carotid artery of the aortic arch in mice (indicated with a red band). The right carotid artery is labelled. 48 h post ligation surgery, mice were euthanised and RNA was extracted from the intima of both carotid arteries (with the contralateral artery as control). A cross-section of a blood vessel is shown, with tunica intima consisting of the endothelial layer. (**B**) Graph shows the fold change expression of genes with respect to the expression in control (Ctl) arteries, as measured by qPCR. Two flow-sensitive genes nitric oxide synthase 3 (*Nos3*) and Krüppel-like Factor 2 (*Klf2*) are shown as positive controls/validations. The fold change in *Fhl2* expression is depicted. *N* = 3 mice. *P = Ctl vs. Nos3, Klf2 is 0.025, 0.0130 respectively, **P = Ctl vs Fhl2 is 0.0056. (**C**) Confocal images of cross sections of contralateral and ligated mouse arteries stained for FHL2, imaged along with Elastin. Zoomed-in images of FHL2 staining from each arterial section are shown, with ‘L’ indicating the lumen and endothelial side. *N* = 2 mice (*n* = 6 sections per condition). (**D**) Mean fluorescence intensity of FHL2 measured in the intima region of each cross-section of mouse contralateral or ligated arteries. *N* = 2 mice (*n* = 6 sections per condition). **P = 0.022. (**E**) Inverted contrast confocal images of TeloHAECs under UF and DF, stained for actin to label stress fibres and FHL2. The regions marked in purple and orange boxes are zoomed in below. (**F**) Fluorescence intensity of FHL2 normalised to actin intensity of each cell, in TeloHAECs under UF and DF. *N* = 2 independent experiments (*n* = 290 for UF, 289 for DF). ****P < 0.0001. (**G**) Normalised expression of FHL2, as measured by qPCR, in TeloHAECs under DF compared to cells under UF. *N* = 3 independent experiments. *P < 0.03. (**H**) Line scans showing FHL2 and actin intensities from a representative region of a stress fibre. (**I**) Graph showing actin intensity vs. FHL2 intensity in a region of a stress fibre. *N* = 3 independent experiments (*n* = 1026 SF regions for UF, 917 SF regions for DF). Slopes are 0.09 and 0.24 for UF and DF, respectively. Shaded error bars are shown. (**J**) Brightfield images of Control (Ctl; -dox) and FHL2 overexpressing (OE; +dox) TeloHAECs under UF are shown. (**K**) Graph shows the aspect ratio (ratio of the major to the minor axis of a cell) of control and FHL2 OE (+dox) cells under UF. *N* = 2 independent experiments (*n* = 253 for Ctl, 236 for FHL2 OE). ****P < 0.0001. (**L**) From brightfield images, the alignment of control and FHL2 OE (+dox) cells under UF are calculated by local gradient orientation and represented as the probability distribution of cells with the flow alignment angles (centred around 0°). Shaded error bars representing standard deviation are shown. *N* = 3 independent experiments (*n* = 22 for UF Ctl, and *n* = 26 for UF FHL2 OE; fields of view, with >100 cells/field). ***P < 0.0008. (**M**) Schematic of a transwell permeability assay, where dextran-FITC (green) was added to the top chamber, and permeability was calculated by measuring green fluorescence in the collected media from the top and bottom chambers. Graph shows relative permeability levels of FHL2 OE cells, normalised with respect to the Ctl. *N* = 5 independent experiments. **P = 0.005. (**N**) Schematic showing that FHL2 levels increase in cells under DF. With increased FHL2 expression, endothelial tissues exhibit high permeability and the lack of cell alignment with flow (disrupted tissue phenotypes), while cells with lower levels of FHL2 (UF or Ctl) have normal physiological tissue phenotypes. In the box-and-whisker plots, the box extends from the 25th to the 75th percentile, the whiskers show the minimum and maximum values, and the line within the box represents the median. Scale: (**B**) 100 µm, (**E**) 20 µm; inset 5 µm. Statistical tests: (**B**) one-way ANOVA followed by Tukey’s multiple comparison’s test. (**D**, **N**) Paired *t* test. (**F**, **G**, **K**–**M**) Student’s *t* test followed by Mann–Whitney test. (**I**) Linear regression. *P < 0.05, **P < 0.005 ****P < 0.0001. In the box-and-whisker plots in (**D**, **F**, **K**), the box extends from the 25th to 75th percentile, the whiskers show minimum and maximum values, and the line within the box represents the median. In (**B**, **G**), the whiskers indicate the minimum and maximum values, and the centre line represents the median.

To assess the regulation of *Fhl2* in vivo, we employed a partial carotid artery ligation mouse model to induce endothelial dysfunction. Here, partial ligation of the left carotid artery induces acute disturbed flow and low shear stress in the artery, resulting in the formation of an atherosclerotic plaque in approximately 2 weeks post-surgery (Cheng et al, 2006; Nam et al, 2009) (Fig. 2A). Most of the effects seen in arteries at 24–48 h post-ligation are attributed to the DF alone, and not atherosclerosis-induced endothelial dysfunction. Exploiting this powerful model system, we isolated the endothelium-enriched intima of contralateral (non-ligated right carotid artery, as control) and ligated mouse arteries 48 h post-surgery, and subsequently extracted RNA from these mouse intima tissues (Fig. 2A). Quantitative PCR revealed that the levels of crucial transcription factors and well-known flow-sensitive genes, *Klf2* and *Nos3*, are downregulated by ~3–4-fold in the intima of ligated arteries (Fig. 2B), which is consistent with Fig. 1 and previous work (Tamargo et al, 2023). In ligated arteries with DF, we observed that *Fhl2* expression increased by >3-fold compared to the contralateral arteries (Fig. 2B). Next, we tested whether these transcriptional changes correlated with protein expression variations in mouse arteries. By immunofluorescence staining for FHL2 in partial ligation mouse models, we observed that the endothelial lining of the ligated mouse artery had a striking increase in FHL2 expression compared to the contralateral control arteries (Fig. 2C,D). As *Fhl2* expression increases within 2 days post ligation, our results strongly suggest that endothelial *Fhl2* expression precedes the onset of an atherosclerotic plaque.

**Figure EV2:**
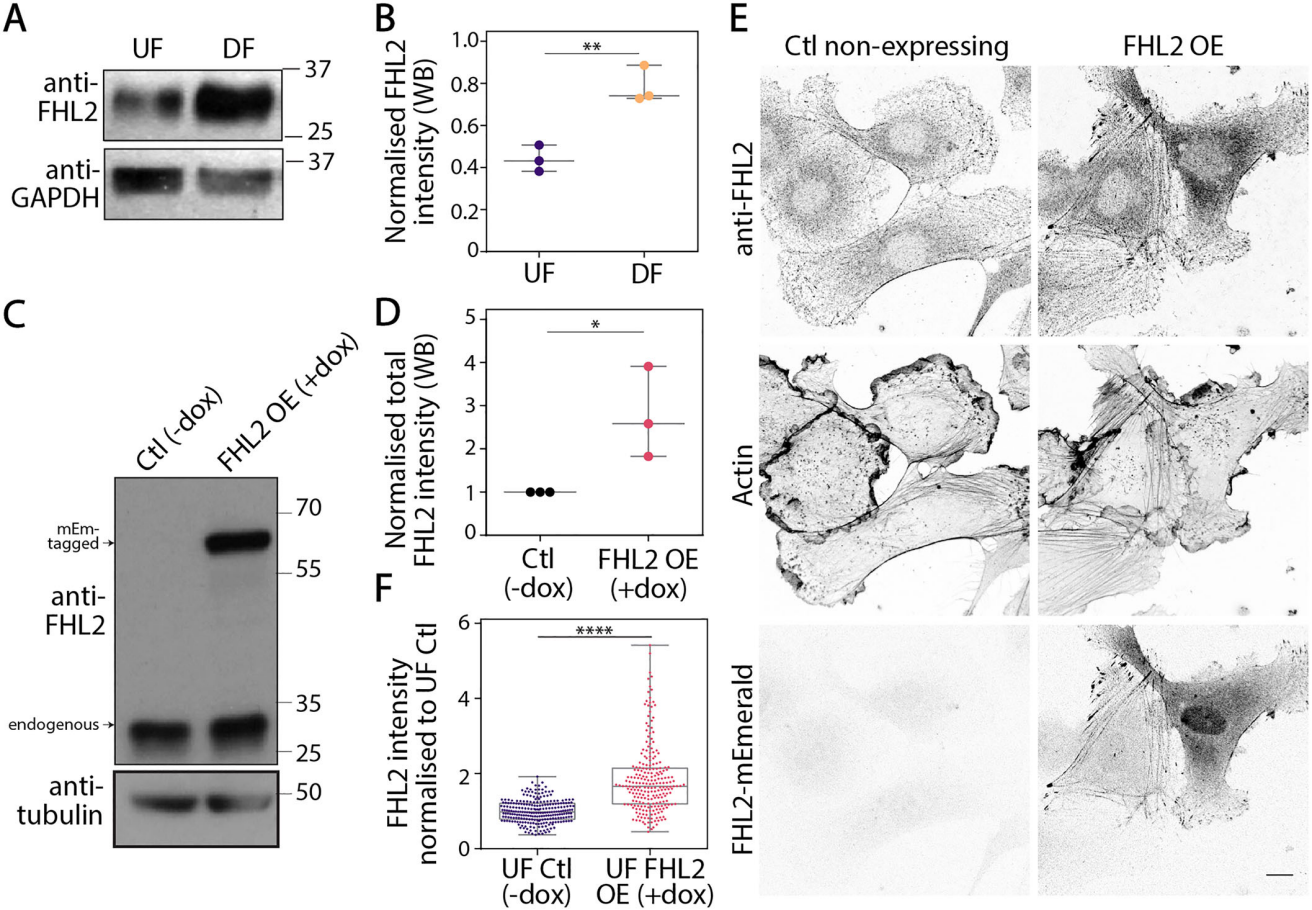
FHL2 expression and localisation in endothelial cells. (**A**) Western blot of lysates from TeloHAECs subjected to UF and DF for 24 h. Samples were analysed by immunoblotting for FHL2 and GAPDH (loading control). (**B**) Normalised FHL2 intensity in UF and DF cells. *N* = 3 independent experiments. Dots represent experimental means, whiskers show the minimum and maximum values, and the line within represents the median. (**C**) Western blot of lysates from Ctl (-dox) and FHL2-overexpressing (+dox; FHL2 OE) cells. Samples were analysed by immunoblotting for FHL2 and tubulin (loading control). FHL2 blotting reveals two bands - endogenous FHL2 (~32 kDa) and overexpressed m-Emerald tagged FHL2 protein (~60 kDa). (**D**) Graph shows the ‘total FHL2 expression levels’ in Ctl and FHL2 OE cells, defined by the sum of the intensities of both bands (FHL2 endogenous + overexpression) normalised to the respective loading control. *N* = 3 independent experiments. Dots represent experimental means, whiskers show the minimum and maximum values, and the line within represents the median. (**E**) Immunostaining of FHL2 and actin, and visualisation of mEmerald-FHL2 tagging in Ctl and FHL2-OE cells. (**F**) Ctl and FHL2 OE cells were subjected to UF and immunostained for FHL2 and actin. Graph shows normalised FHL2 intensity of each cell under UF in both conditions. *N* = 2 independent experiments (*n* = 253 for Ctl and 236 for FHL2 OE). Scale: (**E**) 10 µm. Statistical tests: (**B**, **D**) Unpaired *t* test. (**E**) Unpaired *t* test followed by Mann–Whitney test. ****P < 0.0001, **P < 0.01, *P < 0.05. In the box-and-whisker plots, the box extends from the 25th to 75th percentile, the whiskers show minimum and maximum values, and the line within the box represents the median.

Next, we investigated whether our findings from mouse tissues were observable in monolayers of endothelial cells subjected to 24 h of UF and DF profiles (as described in Fig. 1). High resolution confocal imaging of FHL2 and actin allowed for quantification of changes in overall FHL2 protein levels and its subcellular localization (Fig. 2E). FHL2 protein levels averaged across a cell increased by ~twofold in DF (Fig. 2E,F). This was also validated via western blotting (Fig. EV2A,B). Consistent with the RNA sequencing data and the expression in in vivo mouse intima, qPCR revealed that FHL2 transcription was also increased by ~threefold in DF (Fig. 2G).

Concurrently, the subcellular distribution of FHL2 also changed in response to flow. To quantify this, intensity profiles across stress fibres in each condition were obtained. While robust actin stress fibres were seen in both UF and DF conditions (Fig. 2E), stress fibres in DF conditions were strongly enriched with FHL2 (Fig. 2E,H). To explore whether changes in FHL2 localization arose from differences in stress fibre actin density, we plotted FHL2 intensity as a function of actin intensity for a variety of stress fibres. We observed large stress fibres with high FHL2 intensity in DF cells. On the contrary, in UF, majority of stress fibres were smaller, and the occasional large stress fibres did not contain a proportionally high FHL2 intensity as was seen in DF cells. Taken together with previous work showing FHL2 binds tensed actin (Sun et al, 2020), our results suggest that in cells under DF, actomyosin bundles are highly tense and facilitate the binding of FHL2 to stress fibres (Fig. 2D,H). Our initial analysis revealed that FHL2 expression is higher in endothelial cells experiencing DF, which correlates with athero-like endothelial phenotypes. Therefore, we sought to determine if this increase in FHL2 plays a causative role and is sufficient to induce endothelial dysfunction. To test this, we generated a doxycycline (dox)-inducible endothelial cell line overexpressing FHL2 (TeloHAEC FHL2-mEmerald; FHL2 OE), where the FHL2 protein levels measured by western blotting (Fig. EV2C,D) or immunofluorescence (Fig. EV2E,F) were approximately two times higher than in control cells. This difference in FHL2 expression is similar to the fold change between endogenous FHL2 levels in cells under UF and DF (Fig. 2E,F). We also confirmed that the localisation of FHL2 in the overexpression cells resembled the endogenous protein (Fig. EV2E).

With this system, we investigated whether perturbing FHL2 expression levels alone would result in endothelial dysfunction in cells experiencing UF. We observed that FHL2-overexpressing cells subjected to UF were less elongated compared to the control cells (Fig. 2I,J), with reduced cell alignment in the direction of flow (Fig. 2K). Thus, FHL2 overexpression is sufficient to drive the cell morphological phenotypes, similar to DF conditions, even when subjected to a healthy athero-protective UF.

Due to the FHL2-mediated defects in cell morphology and alignment, we specifically focused on whether changes in FHL2 expression resulted in tissue-scale endothelial dysfunction. A well-established readout of endothelial dysfunction in in vivo as well as in vitro DF models is tissue permeability or leakiness (Orr et al, 2007; Rickman et al, 2023). To measure tissue permeability, we used transwell assays to assess the amount of fluorescent dextran (FITC-dextran 4 kDa) that crossed the endothelial monolayer. We observed that in vitro endothelial tissues with FHL2 overexpression were approximately three times more permeable compared to the control endothelial monolayers (Fig. 2L). Taken together, our results from in vitro and in vivo experiments strongly demonstrate that FHL2 is upregulated in response to DF. We further show that increased FHL2 expression, as seen in DF, promotes tissue permeability (Fig. 2M,N). This suggests a crucial role for FHL2 transcriptional regulation and localisation in regulating endothelial mechanoresponses to DF, by contributing to the onset of athero-like phenotypes.

### FHL2 expression drives the formation of discontinuous focal adherens junctions

**Figure 3:**
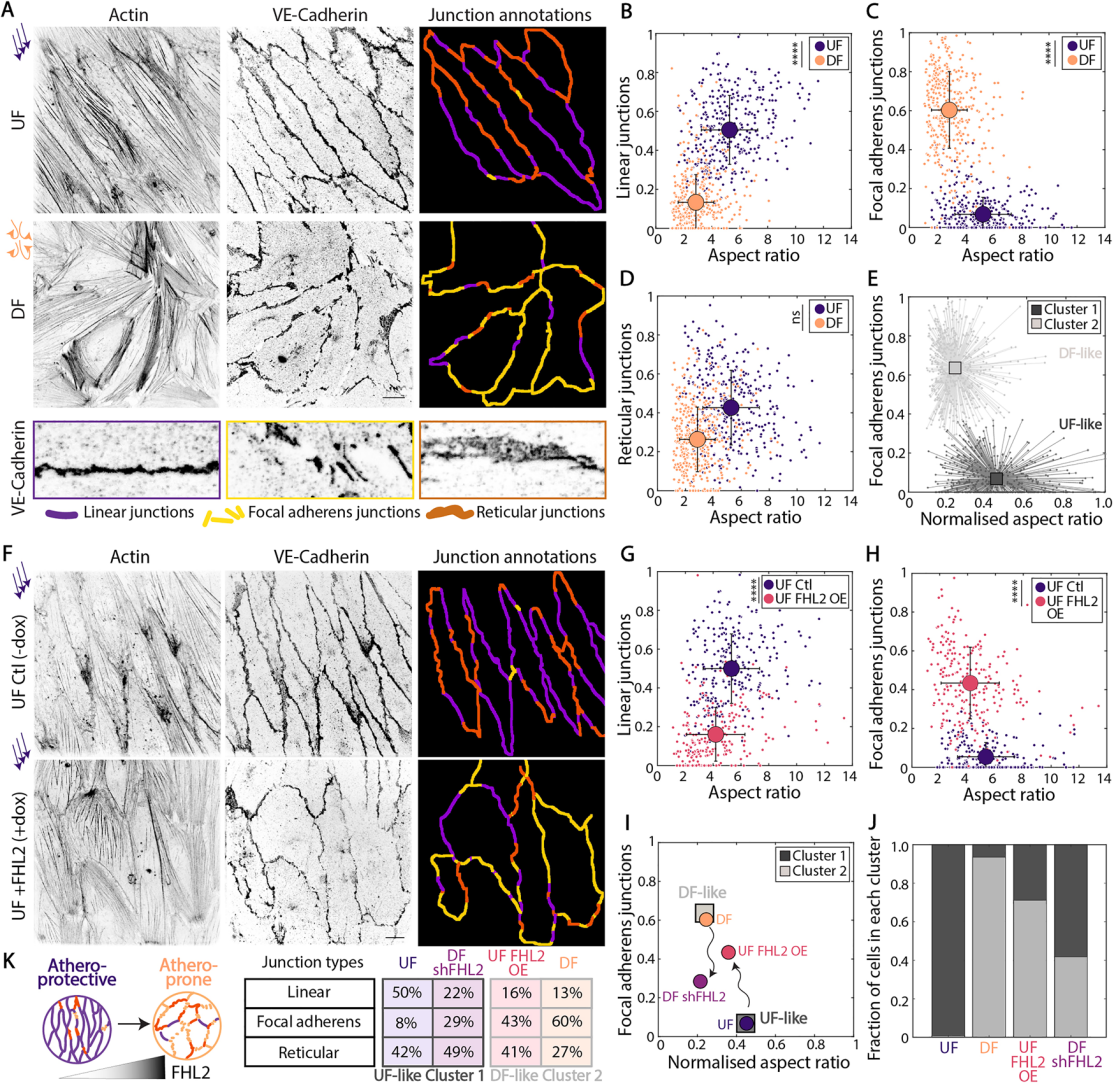
FHL2 overexpression drives focal adherens junction formation, similar to DF cell clusters. (**A**) Inverted contrast confocal images of TeloHAECs under UF and DF, stained for actin and vascular endothelial cadherin, VE-Cadherin (to label adherens junctions). Three cell junction types were annotated using VE-Cadherin staining: i. linear junctions, ii. reticular junctions, iii. focal adherens junctions. Examples of each junction type are shown as zoomed-in inverted contrast images on the bottom. (**B**–**D**) Graphs showing the aspect ratio (ratio of the major to the minor axis of a cell) vs. the fraction of the cell perimeter occupied by linear junctions (**B**), focal adherens junctions (**C**), reticular junctions (**D**). *N* = 3 independent experiments (*n* = 356 for UF, 417 for DF). ns: *P* value not significant, ****P < 0.0001. (**E**) Using measurements of aspect ratio and fractions of the three junction types in (**B**–**D**), cells were clustered using k-means, with *k* = 2. Two clusters and the corresponding data points are shown. *N* > 3 independent experiments (*n* = 1179 cells clustered into cluster 1 or 2). (**F**) Inverted contrast confocal images of control (Ctl; -dox) and FHL2-overexpressing (OE; +dox) TeloHAECs under UF, stained for phalloidin and VE-Cadherin. Annotated junctions are depicted as in (**A**). (**G**–**I**) Graphs showing the aspect ratio (ratio of the major to the minor axis of a cell) vs. the fraction of the cell perimeter occupied by linear junctions (**B**), focal adherens junctions (**C**), reticular junctions (**D**). *N* = 2 independent experiments (*n* = 253 for UF Ctl, 256 for UF FHL2 OE). The UF Ctl means were pooled from five experiments for statistical tests. ****P < 0.0001. (**J**) Centroids of clusters 1 and 2 (from (**E**)) are depicted. The means of FHL2 overexpressing cells under UF and FHL2-knockdown (UF shFHL2 (+dox)) cells are shown, with the aspect ratio normalised according to clusters 1 and 2. Inset shows a bar graph of the fraction of cells (from UF, DF, UF FHL2 OE, and DF shFHL2) in each predefined cluster, using k-means clustering approach. Wavy arrows depict how expression levels of FHL2 tunes the fraction of focal adherens junctions. *N* = 3 for UF, DF, and *N* = 2 independent experiments for FHL2 OE (*n* = 1179 cells from control UF and DF clustered into cluster 1 or 2, 236 for UF FHL2 OE, 225 for DF shFHL2). (**K**) Summary showing junction morphologies under varying FHL2 expression levels and flow profiles. Scale: (**A**, **F**) 20 µm. Statistical tests: (**B**–**D**, **G**, **H**) Unpaired *t* test. In the scatter plots, large central circles represent the mean, and error bars indicate the standard deviation (SD) for both aspect ratio (*x* axis) and fraction of junctions (*y* axis).

Endothelial permeability and barrier function are tightly regulated by the stability and integrity of adherens junctions (Friedrich et al, 2019; Huveneers et al, 2012; Schulte et al, 2011). In atherosclerotic arteries, the breakdown of cell junctions is a classical marker of endothelial dysfunction (Tamargo et al, 2023; Dai et al, 2004). By visualising VE-Cadherin in endothelial cells under UF and DF, three junction morphologies are observed (Fig. 3A): (1) linear junctions which are thin, intact, well-formed and stable; (2) focal adherens junctions which represent the dynamic and discontinuous junctions, with parts of the junctions along cell borders being broken (Oldenburg et al, 2015; Millán et al, 2010); and (3) reticular junctions which are wider, overlapping and honeycomb-like structures (Fernández-Martín et al, 2012).

**Figure EV3:**
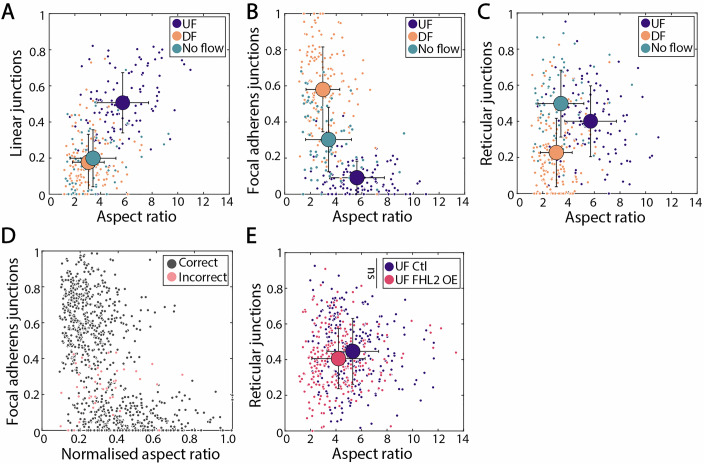
Junction morphology analysis and unbiased clustering of cell populations under varying flow profiles. (**A**) Graphs showing the aspect ratio (ratio of the major to the minor axis of a cell) vs. the fraction of the cell perimeter occupied by linear junctions (**A**), focal adherens junctions (**B**), reticular junctions (**C**). *n* = 66 for No Flow, 106 for UF, 149 for DF. (**D**) Using measurements of aspect ratio and fractions of the three junction types in Fig. 3B–D, cells were clustered using k-means, with *k* = 2. Two clusters and the corresponding data points are shown. *N *> 3 independent experiments (*n *= 1179 cells clustered into Cluster 1 or Cluster 2). Graphs showing the normalised aspect ratio vs. the fraction of the cell perimeter occupied by focal adherens junctions. Each cell is represented as a dot, and all the WT cells under UF and DF from across various experiments were clustered and are represented on the graph. Cells are colour coded based on whether they were correctly categorised into Clusters 1 or 2, based on the experimental condition. All the grey dots show that the cells that were under UF or DF were clustered into UF-like Cluster 1 or DF-like Cluster 2, respectively. The pink dots represent a mismatch in the clustering where the cells are clustered different from the actual experimental condition. (**E**) Graphs showing the aspect ratio (ratio of the major to the minor axis of a cell) vs. the fraction of the cell perimeter occupied by reticular junctions. *N* = 2 independent experiments (*n* = 253 for UF Ctl, 256 for UF FHL2 OE). Statistical tests: Unpaired *t* test. ns: *P* value not significant. In the box-and-whisker plots, the box extends from the 25th to 75th percentile, the whiskers show minimum and maximum values, and the line within the box represents the median.

First, we systematically categorised junction morphologies using VE-Cadherin immunostaining images of in vitro endothelial tissues in UF and DF as well as in the absence of flow. As described in Fig. 1C as well as previous in vivo studies (Waters et al, 2011), a major parameter distinguishing UF and DF phenotypes is the cell aspect ratio or cell elongation. Thus, by plotting the cell aspect ratio versus the fraction of linear, reticular or focal adherens junctions per cell, we identified different cell populations that represent UF and DF conditions distinctly (Fig. 3B–D). We show that in healthy endothelial tissues under UF, cells exhibited a high proportion of their perimeter marked by linear junctions (~50%; Fig. 3B), and a very low fraction of discontinuous focal adherens junctions (~8%; Fig. 3C). On the other hand, cells under DF have very different junction morphologies, with focal adherens junctions dominating the phenotype, occupying ~60% of the junction perimeter (Fig. 3C). Further, the fraction of the perimeter occupied by reticular junctions was highly similar between cells under UF and DF (Fig. 3D). As a control, we also measured junction morphologies in the absence of flow (no flow). Under no flow conditions, we observed a decrease in linear junctions, accompanied by a higher fraction of discontinuous focal adherens junctions as compared to cells exposed to UF (Fig. EV3A,B). These junction morphologies are similar to DF phenotypes, although less pronounced. Furthermore, cells under no flow exhibited higher fraction of reticular junctions (Fig. EV3C). Such junction phenotypes are consistent with previous studies showing that cells experiencing low shear or static conditions exhibit discontinuous adherens junctions (Noria et al, 1999; Oldenburg et al, 2015), similar to athero-like DF phenotypes. Our results represent a high resolution and quantitative characterisation of the adherens junction morphologies, where the abundance of the different junction classes serves as key readouts of endothelial tissue phenotypes under UF and DF. Thus, our findings strongly demonstrate that in endothelial tissues under DF, there is a tendency to build focal adherens junctions, which in turn, results in high tissue permeability (Fig. 2M).

Next, in an unbiased way, we sought to classify cells into clusters with the objective of distinctly representing cell populations solely based on the similarities and differences between each cell/data point. We performed an unsupervised k-means clustering with multiple parameters of cell and junction morphologies measured in endothelial tissues (aspect ratio, linear, reticular, and focal adherens junction fractions). This analysis revealed two strikingly distinct clusters which were nearly identical to grouping by UF and DF conditions (Figs. 3E and EV3D). We, therefore, designated these clusters as UF-like Cluster 1 (C1) and DF-like Cluster 2 (C2; Fig. 3E).

With this in-depth characterisation of endothelial junction morphologies during flow-sensing, we then explored whether FHL2 upregulation was sufficient to drive athero-like morphological phenotypes, even when in vitro endothelial tissues were subjected to athero-protective UF for 24 h. Using our analysis pipeline described above, we characterised cell junction morphologies from VE-Cadherin immunostaining images of control and FHL2-overexpressing cells subjected to UF. FHL2-overexpressing cells under UF displayed a significant increase in the formation of discontinuous focal adherens junctions, with a smaller amount of linear cell junctions (Fig. 3F–H). By plotting these junction parameters with respect to the aspect ratio, we identify that the population of cells overexpressing FHL2 are strikingly distinct from the control cells. In particular, FHL2 overexpression cells displayed fewer linear junctions (~16%; Fig. 3F,G) and an augmented proportion of focal adherens junctions (~43%; Fig. 3F,H). Moreover, the proportion of reticular junctions between the control and FHL2-overexpressing cells under UF was not significantly different (~42% and 41%, respectively; Fig. EV3E). Overall, our results strongly demonstrate that high FHL2 expression drives athero-like endothelial junction phenotypes, even when subjected to healthy UF.

**Figure EV4:**
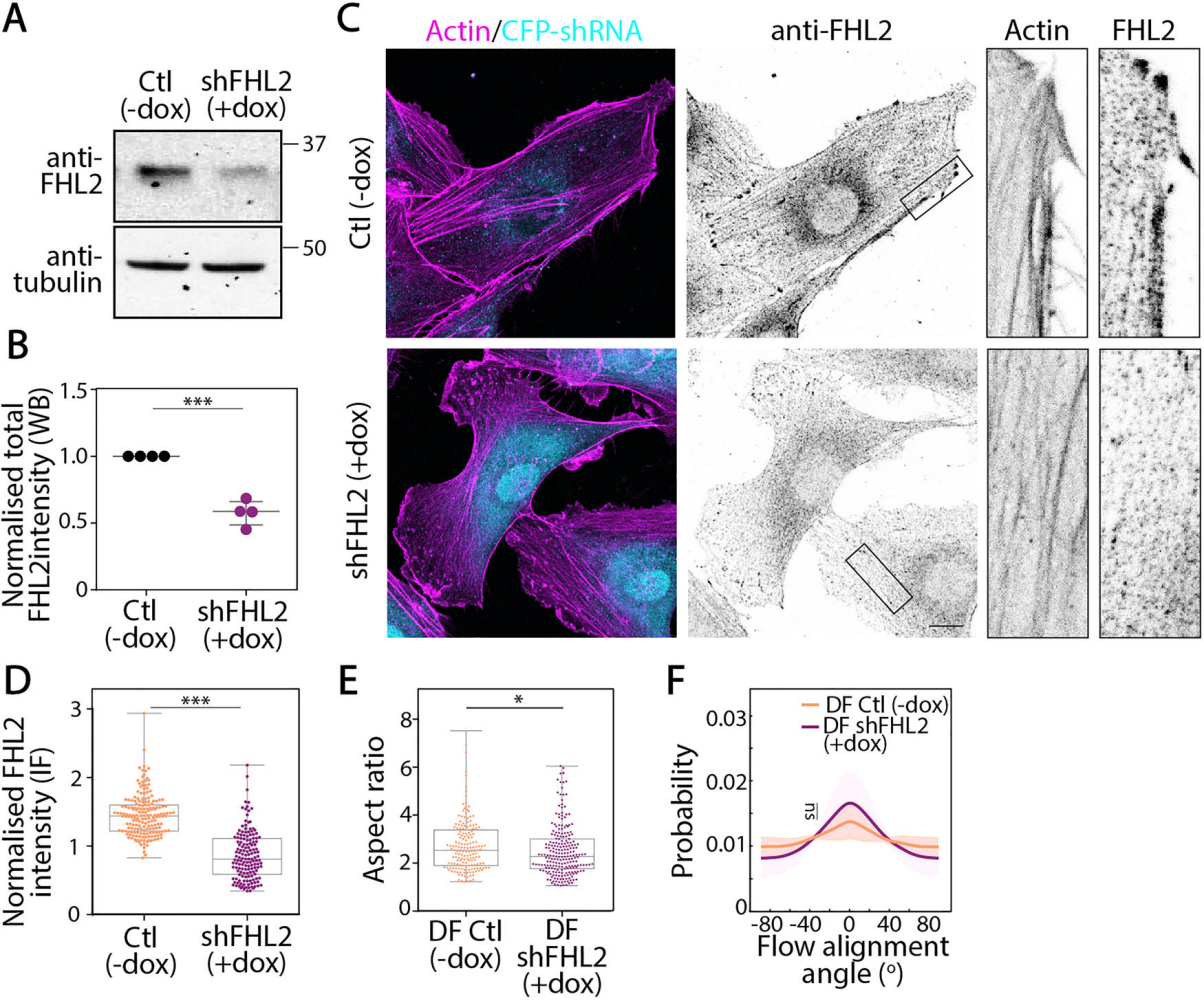
Knockdown of FHL2 in endothelial tissue response to DF. (**A**) Western blot of lysates from control (Ctl; -dox) and FHL2-knockdown (shFHL2; +dox) TeloHAECs. Samples were analysed by immunoblotting for FHL2 and tubulin (loading control). (**B**) Normalised FHL2 intensity in Ctl and shFHL2 cells. *N* = 4 independent experiments. Dots represent experimental means, whiskers show the minimum and maximum values, and the line within represents the median. (**C**) Inverted contrast images of Ctl and shFHL2 cells immunostained for actin and FHL2. (**D**) Fluorescence intensity of FHL2 normalised to actin intensity of each cell, in Ctl and shFHL2 TeloHAECs. *N* = 5 independent experiments (*n* = 187 for Ctl and 151 for shFHL2). (**E**) Graph shows the aspect ratio (ratio of the major to the minor axis of a cell) of control and shFHL2 (+dox) cells under DF. *N* = 2 independent experiments (*n* = 171 for Ctl, 225 for shFHL2). (**F**) From brightfield images, the alignment of Ctl and shFHL2 cells under DF are calculated by local gradient orientation and represented as the probability distribution of cells with the flow alignment angles (centred around 0°). Shaded error bars representing standard deviation are shown. *N* = 1 independent experiment (*n* = 32 for UF Ctl, and *n* = 39 for shFHL2; fields of view, with >100 cells/field). Scale: (C) 10 µm. Statistical tests: (**B**) Unpaired *t* test. (**D**) Paired, two-tailed *t* test. (**E**, **F**) Unpaired *t* test followed by Mann–Whitney test. *P < 0.05, **P < 0.01, ***P < 0.001, ns: *P* value not significant. In the box-and-whisker plots, the box extends from the 25th to 75th percentile, the whiskers show minimum and maximum values, and the line within the box represents the median.

**Figure EV5:**
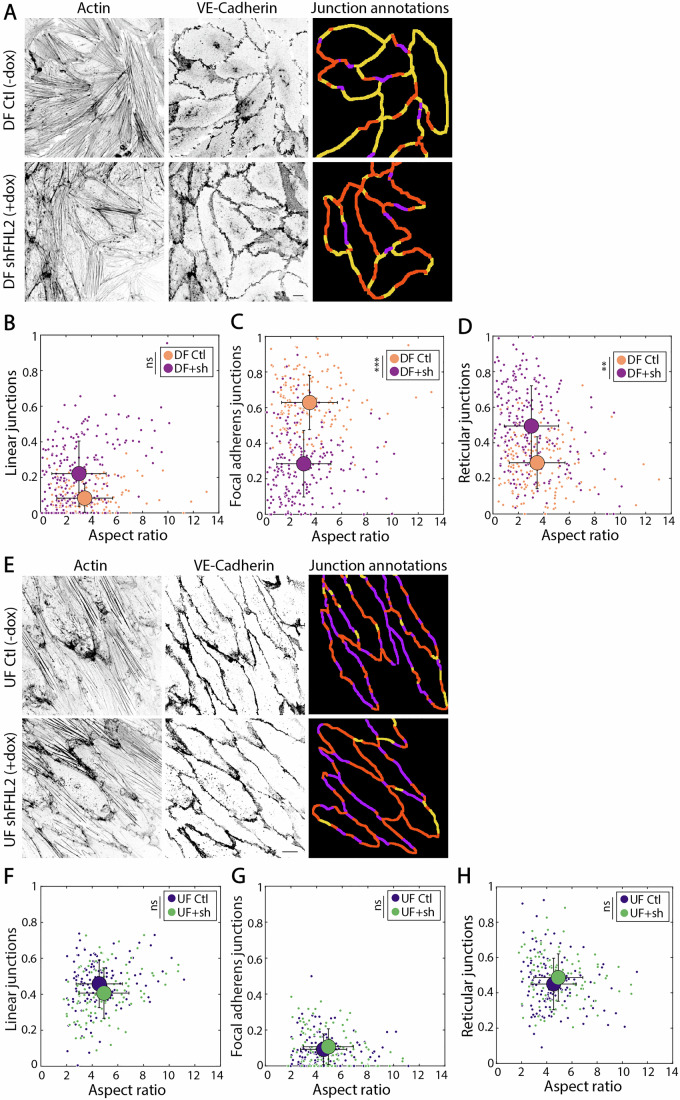
Knockdown of FHL2 partially reverses focal adherens junction morphology without any effects on control conditions. (**A**) Inverted contrast confocal images of control (Ctl; -dox) and shFHL2 TeloHAECs under DF, stained for phalloidin and VE-Cadherin. Annotated junctions are depicted as in Fig. 3A (purple: linear junctions, yellow: focal adherens junctions, orange: reticular junctions). (**B**–**D**) Graphs showing the aspect ratio (ratio of the major to the minor axis of a cell) vs. the fraction of the cell perimeter occupied by linear junctions (**B**), focal adherens junctions (**C**), reticular junctions (**D**). *N* = 2 independent experiments (*n* = 171 for DF Ctl, 225 for DF shFHL2). The DF Ctl means were pooled from 5 experiments for statistical tests. (**E**) Inverted contrast confocal images of control (Ctl; -dox) and shFHL2 TeloHAECs under UF, stained for phalloidin and VE-Cadherin. Annotated junctions are depicted as in Fig. 3A (purple: linear junctions, yellow: focal adherens junctions, orange: reticular junctions). (**F**–**H**) Graphs showing the aspect ratio (ratio of the major to the minor axis of a cell) vs. the fraction of the cell perimeter occupied by linear junctions (**F**), focal adherens junctions (**G**), reticular junctions (**H**). *N* = 2 independent experiments (*n* = 135 for UF Ctl, 107 for UF shFHL2). The UF Ctl means were pooled from five experiments for statistical tests. Scale: (**A**, **E**) 10 µm. Statistical tests: (**B**–**D**, **F**–**H**) Unpaired *t* test followed by Mann–Whitney test. **P < 0.01, ***P < 0.001, ns: *P* value not significant.

We then assessed whether the athero-like junction morphologies seen in DF can be rescued by FHL2 knockdown. To this end, we generated a doxycycline (dox)-inducible endothelial cell line expressing tet-on shRNA for FHL2 (TeloHAEC shFHL2), where FHL2 protein levels are significantly reduced, as measured by western blotting (Fig. EV4A,B) and immunofluorescence (Fig. EV4C,D). FHL2 knockdown cells under DF did not alter the cell aspect ratio or alignment (Fig. EV4E–G); however, we observed a significantly higher fraction of the junction perimeter occupied by linear junctions (~22%; Fig. EV5A,B) and a much lower fraction of focal adherens junctions (~29%; Fig. EV5A,C) compared to control cells under DF. There was also a significant increase in the fraction of reticular junctions in the FHL2-knockdown cells compared to control cells under DF (Fig. EV5A,D). We also verified that there were no significant changes in the three junction morphologies in FHL2-knockdown cells in UF as compared to the control endothelial monolayers (Fig. EV5E–H). Altogether, our results show that reducing FHL2 expression partially rescues the athero-like phenotypes, by restoring linear junction morphologies.

**Figure EV6:**
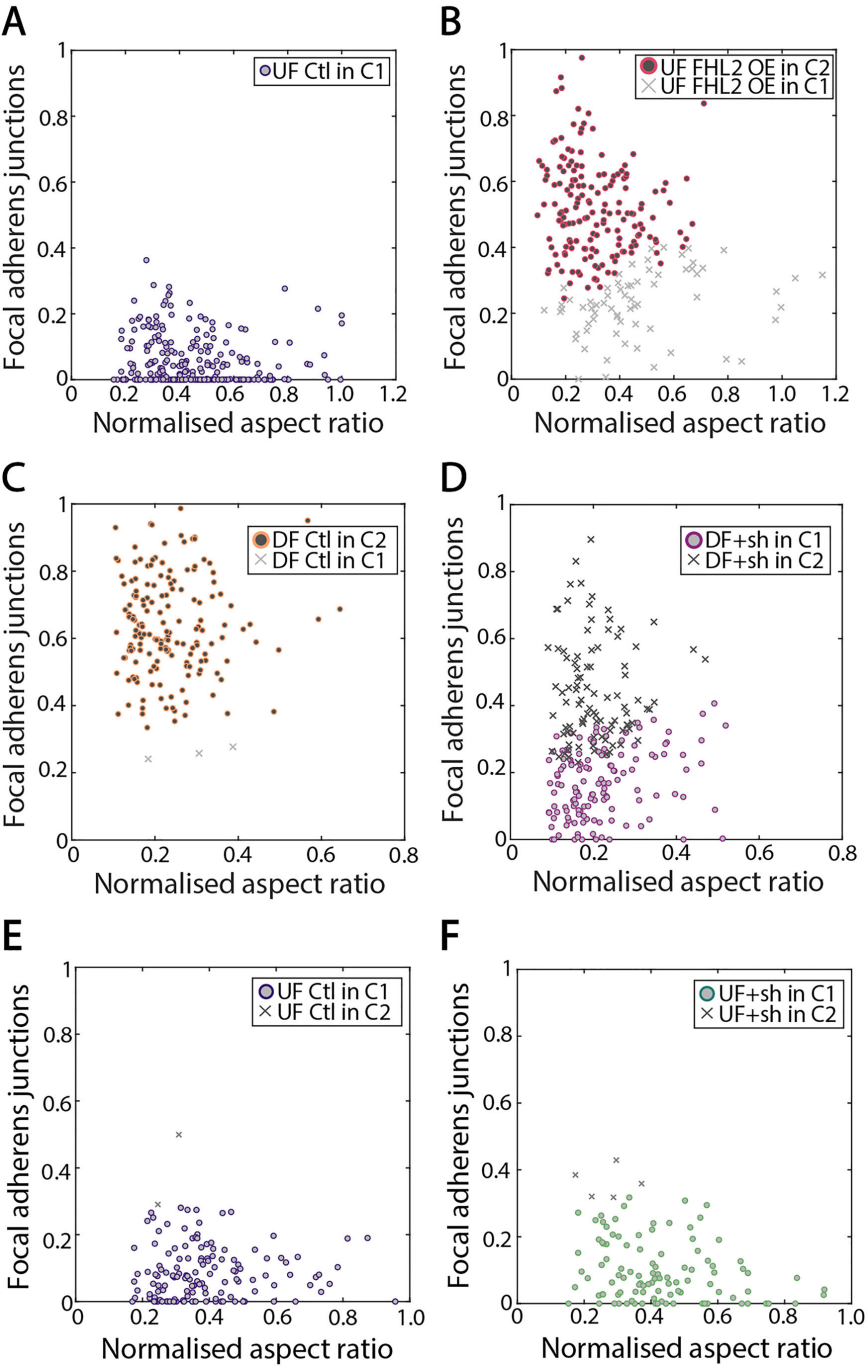
Clustering analysis of cell populations in UF and DF revealed that FHL2 OE cells are highly represented in DF-like cluster. (**A**, **B**) UF Ctl and FHL2 OE cells were clustered (unbiased) into the pre-defined UF-like Cluster 1 (C1) or DF-like Cluster 2 (C2). Graphs showing the normalised aspect ratio vs. the fraction of the cell perimeter occupied by focal adherens junctions in (**A**) Ctl, (**B**) FHL2 OE cells. (**A**) UF Ctl cells completely belong to UF-like Cluster 1. (**B**) FHL2 OE cells were clustered into the predefined clusters, and the circles represent the dominant cluster (in this case, DF-like Cluster 2; C2), while the ‘x’ depicts the minority cluster (in this case, UF-like Cluster 1; C1). (**C**, **D**) DF Ctl and shFHL2 cells were clustered (unbiased) into the pre-defined UF-like Cluster 1 (C1) or DF-like Cluster 2 (C2). Graphs showing the normalised aspect ratio vs. the fraction of the cell perimeter occupied by focal adherens junctions in (**C**) Ctl, (**D**) shFHL2 cells. (**C**) DF Ctl cells almost completely belong to DF-like Cluster 2 (circles represent the dominant cluster, in this case, DF-like Cluster 2, C2; while the ‘x’ depicts the minority cluster (in this case, UF-like Cluster 1; C1). (**D**) DF shFHL2 cells were clustered into the predefined clusters, and the circles represent the dominant cluster (in this case, UF-like Cluster 1; C1), while the ‘x’ depicts the minority cluster (in this case, DF-like Cluster 2; C2). (**E**, **F**) UF Ctl and shFHL2 cells were clustered (unbiased) into the pre-defined UF-like Cluster 1 (C1) or DF-like Cluster 2 (C2). Graphs showing the normalised aspect ratio vs. the fraction of the cell perimeter occupied by focal adherens junctions in (**E**) Ctl, (**F**) shFHL2 cells. (**E**) UF Ctl cells almost completely belong to UF-like Cluster 1 (circles represent the dominant cluster, in this case, UF-like Cluster 1, C1; while the ‘x’ depicts the minority cluster (in this case, DF-like Cluster 2; C2). (**F**) UF shFHL2 cells were clustered into the predefined clusters, and the circles represent the dominant cluster (in this case, UF-like Cluster 1; C1), while the ‘x’ depicts the minority cluster (in this case, DF-like Cluster 2; C2).

Finally, to compare the effects of varying FHL2 expression on the adherens junction morphologies, we plotted the experimental means of all conditions, along with the centroids of Clusters 1 and 2 (Fig. 3I). As indicated by the black arrows, tuning FHL2 levels in the absence of external mechanical input drives phenotypes towards UF-like and DF-like Clusters 1 and 2. Furthermore, with the clustering approach described in Fig. 3E, in each condition, we determined the number of cells in UF-like Cluster 1 and DF-like Cluster 2 (Fig. 3J). Strikingly, in conditions with high FHL2 levels (~94% of DF, ~70% of UF FHL2 OE), a majority of cells were found in the DF-like Cluster 2, and on the other hand, in conditions with low FHL2, most cells (~99% of UF) belonged to the UF-like Cluster 1 (Figs. 3J and EV6A,B). In addition, with FHL2-knockdown under DF, a high fraction (~59%) of cells reverted to UF-like Cluster 1 (Figs. 3J and EV6C,D), suggesting rescue of athero-like junction phenotypes. Finally, there was no difference between FHL2-knockdown and control cells under UF, with ~99% of cells represented in Cluster 1 (Fig. EV6E,F).

Overall, our results strongly demonstrate that FHL2 expression is necessary and sufficient to drive athero-like phenotypes (Fig. 3K). We also find that reducing FHL2 levels partially reverses athero-like phenotypes induced by DF, through the abrogation of leaky focal adherens junctions (Fig. 3K). Taken together, we show that FHL2 expression tunes endothelial function through the remodelling of adherens junctions.

### Force-sensitive FHL2 binding to stress fibres drives athero-like endothelial phenotypes

**Figure 4:**
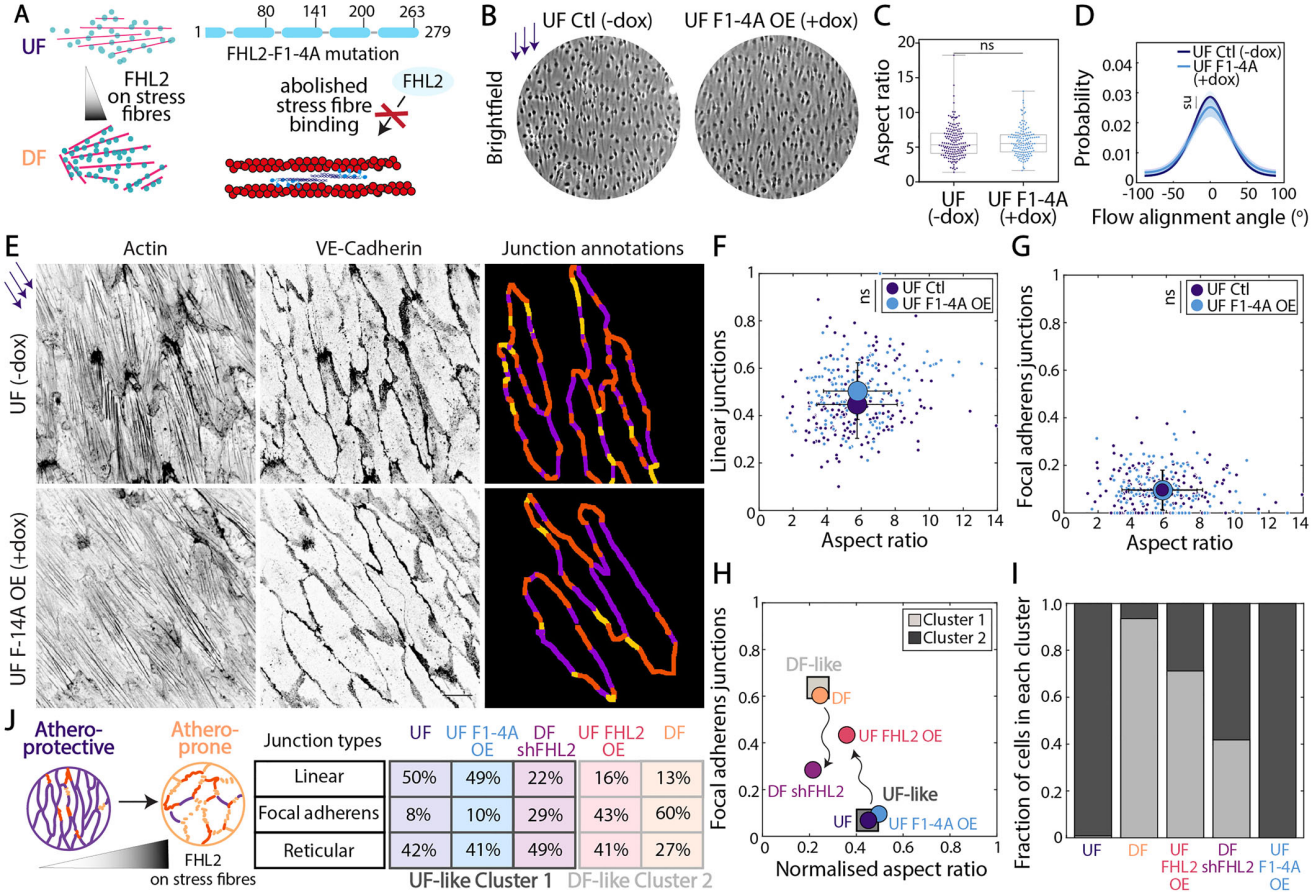
FHL2 binding to stress fibres is essential for promoting athero-prone phenotypes. (**A**) Schematic of the four phenylalanine point mutations created in FHL2, to generate FHL2 F1-4A (mutations previously described in (Sun et al, 2020)). (**B**) Inverted contrast brightfield images of TeloHAECs overexpressing mEmerald tagged- FHL2 WT or FHL2 F1-4A. (**C**) Graph shows the aspect ratio (ratio of the major to the minor axis of a cell) of control (-dox) and F1-4A overexpressing (OE; +dox) TeloHAECs under UF and DF. *N* = 2 independent experiments (*n* = 143 for Ctl and 167 for F1-4A OE). ns: *P* value (=0.64) not significant. (**D**) From brightfield images, the alignment of control and F1-4A overexpressing cells under UF are calculated by local gradient orientation, and represented as the probability distribution of cells with the flow alignment angles (centred around 0°). Shaded error bars representing standard deviation are shown. *N* = 3 independent experiments (*n* = 34 for UF Ctl, and *n* = 39 for UF F1-4A OE; fields of view, with >100 cells/field). ns: *P* value (=0.22) not significant. (**E**) Inverted contrast confocal images of control and F1-4A overexpressing TeloHAECs under UF, stained for phalloidin and VE-Cadherin. Annotated junctions (three types) are depicted. (**F**–**H**) Graphs showing the aspect ratio (ratio of the major to the minor axis of a cell) vs. the fraction of the cell perimeter occupied by linear junctions (**F**), focal adherens junctions (**G**). *N* = 2 independent experiments (*n* = 143 for UF Ctl, 167 for UF F1-4A OE). One data point belonging to UF Ctl (aspect ratio 18.25, linear 0.62, focal adherens 0.01) used in the analyses is excluded from the graph for representation purposes. The UF Ctl means were pooled from five experiments for statistical tests. ns: *P* value (=0.209 for (**F**), 0.806 for (**G**)) not significant. (**I**) Centroids of Clusters 1 and 2 (from Fig. 3) are depicted. The means of F1-4A and FHL2 overexpressing cells under UF, and FHL2 knockdown cells under DF are shown, with the aspect ratio normalised according to Clusters 1 and 2. Inset shows a bar graph of the fraction of cells (from UF, DF, UF FHL2 OE, and DF shFHL2, UF F1-4A OE) in each predefined cluster, using k-means clustering approach. Wavy arrows depict how expression levels of FHL2 tune the fractions of focal adherens junctions. *N* = 3 for UF, DF, and *N* = 2 independent experiments for F1-4A OE (*n* = 1179 cells from control UF and DF clustered into cluster 1 or 2, 236 for UF FHL2 OE, 225 for DF shFHL2, 167 for UF F1-4A OE). In the box-and-whisker plot, the box extends from the 25th to the 75th percentile, the whiskers show the minimum and maximum values, and the line within the box represents the median. (**J**) Summary showing junction morphologies under varying FHL2 expression levels and flow profiles. Scale: (**E**) 20 µm. Statistical tests: (**C**, **D**) Student’s *t* test followed by Mann–Whitney test. (**F**, **G**) Unpaired Student’s *t* test. ns: *P* value not significant. In the box-and-whisker plots, the box extends from the 25th to 75th percentile, the whiskers show minimum and maximum values, and the line within the box represents the median. In the scatter plots, large central circles represent the mean, and error bars indicate the standard deviation (SD) for both aspect ratio (*x* axis) and fraction of junctions (*y* axis).

**Figure EV7:**
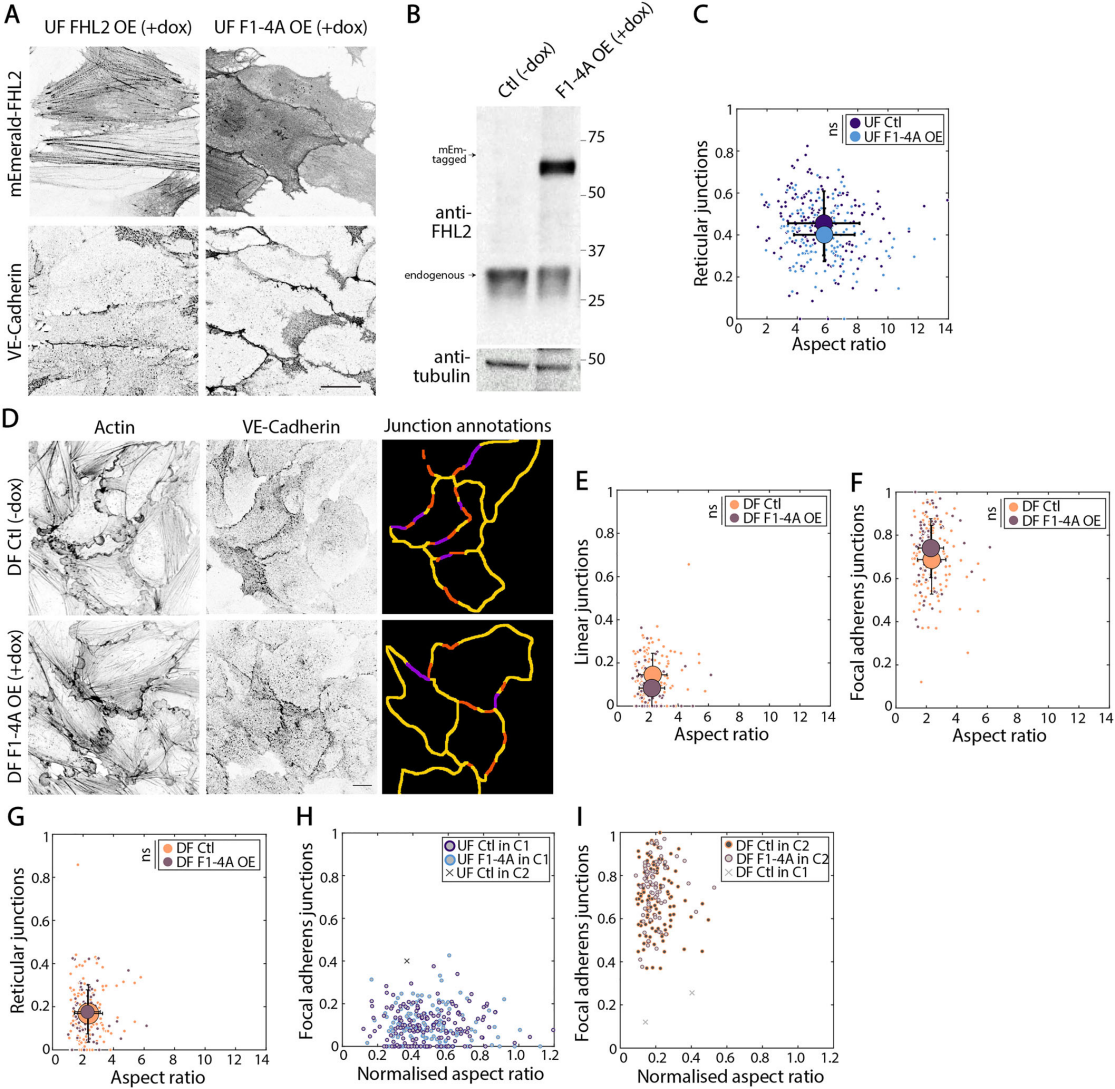
FHL2 mutant and clustering analysis shows its similarity with UF-like Cluster 1. (**A**) Inverted contrast images of FHL2-overexpressing (FHL2 OE; +dox) and FHL2-F1-4A-overexpressing (F1-4A OE; +dox) cells immunostained for VE-Cadherin. mEmerald tagging of FHL2 protein is visualised. (**B**) Western blot of lysates from control (Ctl; -dox) and F1-4A OE (+dox) cells. Samples were analysed by immunoblotting for FHL2 and tubulin (loading control). FHL2 blotting reveals two bands - endogenous FHL2 (~ 32 kDa) and overexpressed m-Emerald tagged FHL2-F1-4A mutant protein (~60 kDa). *N* = 2 independent experiments. (**C**) Graph showing the aspect ratio (ratio of the major to the minor axis of a cell) vs. the fraction of the cell perimeter occupied by reticular junctions. *N* = 2 independent experiments (*n* = 143 for UF Ctl, 167 for UF F1-4A OE). One data point belonging to UF Ctl (aspect ratio 18.25, linear 0.62, focal adherens 0.01) used in the analyses is excluded from the graph for representation purposes. The UF Ctl means were pooled from 5 experiments for statistical tests. (**D**) Inverted contrast confocal images of control (Ctl; -dox) and FHL2-F1-4A-overexpressing (F1-4A OE; +dox) TeloHAECs under DF, stained for phalloidin and VE-Cadherin. Annotated junctions are depicted as in Fig. 3A (purple: linear junctions, yellow: focal adherens junctions, orange: reticular junctions). (**E**–**G**) Graphs showing the aspect ratio (ratio of the major to the minor axis of a cell) vs. the fraction of the cell perimeter occupied by linear junctions (**E**), focal adherens junctions (**F**), reticular junctions (**G**). *N* = 2 independent experiments (*n* = 121 for DF Ctl, 74 for DF F1-4A OE). (**H**, **I**) UF Ctl and UF F1-4A OE cells were clustered (unbiased) into the pre-defined UF-like Cluster 1 (C1) or DF-like Cluster 2 (C2). Graphs showing the normalised aspect ratio vs. the fraction of the cell perimeter occupied by focal adherens junctions in (**H**) Ctl, (**I**) F1-4A OE cells. UF Ctl and UF F1-4A OE cells almost completely belong to UF-like Cluster 1 (circles represent the dominant cluster, in this case, UF-like Cluster 1, C1; while the ‘x’ depicts the minority cluster (in this case, DF-like Cluster 2; C2). Scale: (**A**, **D**) 20 µm. Statistical tests: (**C**, **E**–**G**) Unpaired *t* test followed by Mann–Whitney test. ns: *P* value not significant.

Given that FHL2 is enriched on stress fibres in DF or FHL2 overexpression, we investigated whether this localisation arises from its force-dependent recruitment to actin filaments (Sun et al, 2020; Winkelman et al, 2020). Thus, we abrogated the mechanosensitive localisation of FHL2 to F-actin by generating a doxycycline (dox)-inducible TeloHAEC cell line with four phenylalanine-to-alanine point-mutations (Sun et al, 2020), F80A, F141A, F200A, and F263A (TeloHAEC FHL2-F1-4A-mEmerald, F1-1A OE; Figs. 4A and EV7A,B).

First, we investigated whether the mutant F1-4A-overexpressing cells, which lacks binding to stress fibres, has the potential to drive endothelial dysfunction like the WT FHL2 protein. In UF conditions, F1-4A-overexpressing cells recapitulated the alignment and aspect ratio observed in control cells (Fig. 4B–D). Further, F1-4A-overexpressing cells had a high proportion of linear (~49%; Fig. 4E,F) and reticular junctions (~41%; Fig. EV7C), and a very low fraction of focal adherens junctions (~10%; Fig. 4G). This is in sharp contrast to FHL2-overexpressing cells under UF that exhibited predominantly focal adherens junctions (Fig. 2I–K). Overall, in terms of cell alignment as well as junction morphologies, the overexpression of mutant F1-4A that lacks stress fibre-binding did not show significant differences compared to control (Fig. 4E–G). We also verified that the F1-4A-overexpression in cells under DF (Fig. EV7D) exhibited high fraction of focal adherens junctions and very low linear junction morphologies, similar to control cells under DF. In this case, the endogenous FHL2 expression in DF is still prevalent, and contributes to endothelial dysfunction (Fig. EV7E–G). Thus, our results show that mutating the residues important for force-sensitive binding of FHL2 to actin abrogates its role in promoting an athero-like phenotypes. In other words, these athero-like phenotypes arise only with high FHL2 expression and its localisation to stress fibres.

Finally, to compare the effects of stress fibre-bound FHL2 and varying FHL2 levels on junction morphologies, we depicted the mean of F1-4A-overexpressing cells on the graph shown in Fig. 3I. Here, F1-4A-overexpressing cells were strikingly similar to UF conditions (Fig. 4H). We further determined the fraction of F1-4A-overexpressing cells in each cluster and found that all the cells belonged to the UF-like cluster 1 (Figs. 4I and EV7H). On the other hand, in DF, all the F1-4A-overexpression cells still fall in Cluster 2, possibly due to the basally high FHL2 levels induced by flow (Fig. EV7I).

Overall, our results indicate that the overexpression of cytoplasmic, non-stress fibre binding mutant of FHL2 had no detrimental effects on cell aspect ratio, alignment, or junction morphologies when subjected to UF (Fig. 4J). This also implies that in cells where FHL2 levels are low and there is no enrichment on stress fibres, the cellular phenotypes mimic control cells under UF. Taken together with our previous findings, we demonstrate a crucial function for FHL2 expression and localisation to stress fibres in promoting an athero-like phenotypes.

### FHL2 promotes actomyosin contractility

Given that FHL2 expression drives cell junction remodelling and tissue permeability, we sought to understand the mechanisms governing these tissue-scale phenotypes and function. We know that adherens junctions are coupled to the actomyosin cytoskeleton; as such, they are strongly modulated by changes in RhoGTPase signalling (Huveneers et al, 2012; Liu et al, 2010; Wójciak-Stothard et al, 2001; Angulo-Urarte et al, 2018; Cerutti and Ridley, 2017). For instance, in endothelial cells, focal adherens junctions arise at the ends of actomyosin stress fibres (Millán et al, 2010; Wójciak-Stothard et al, 2001). Thus, we investigated whether changes in FHL2 expression modulates cellular contractility.

**Figure 5:**
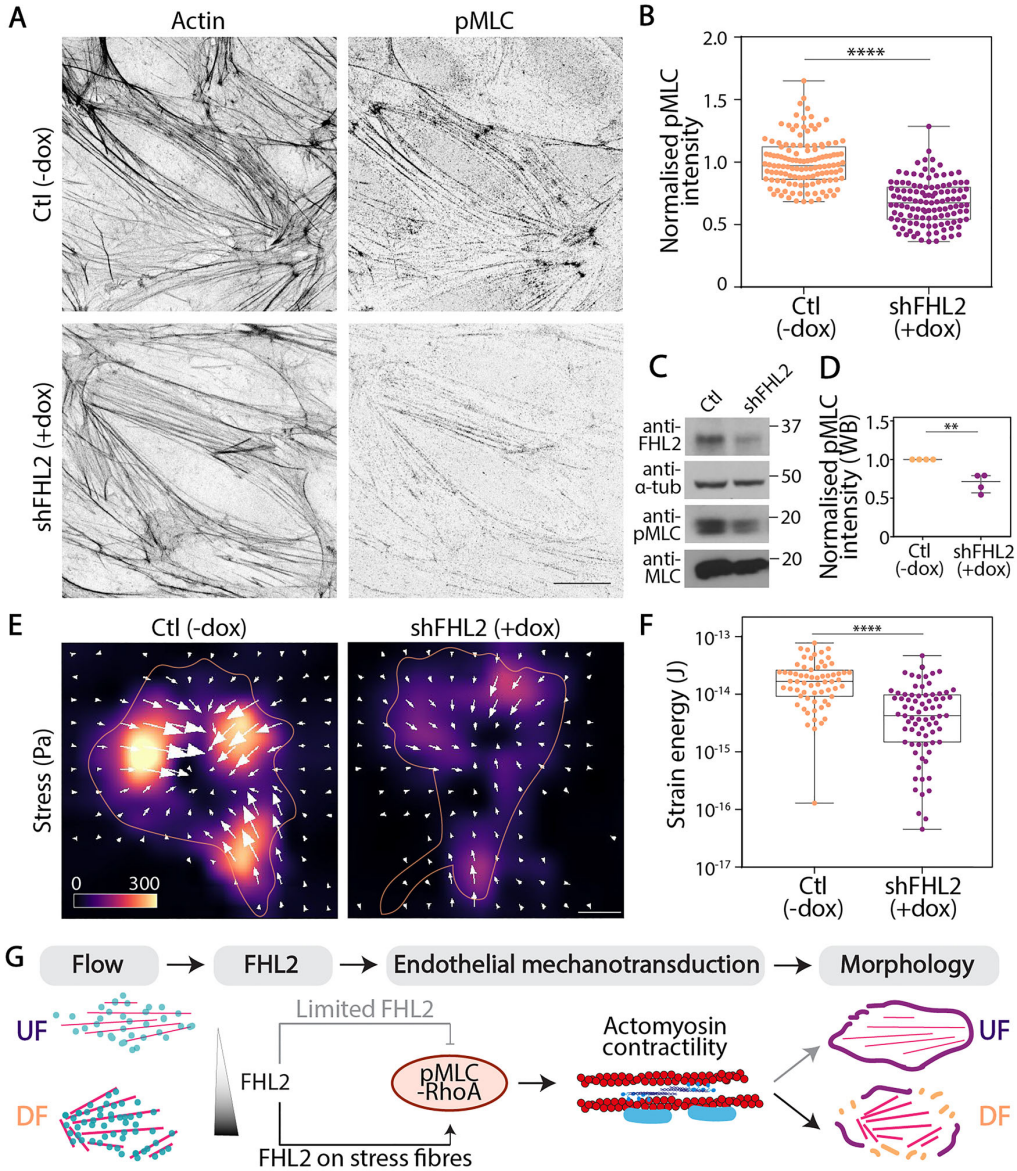
FHL2 expression promotes actomyosin contractility. (**A**) Inverted contrast images of TeloHAECs immunostained for phalloidin (to label actomyosin stress fibres) and phosphorylated (serine 19) myosin light chain (pMLC). (**B**) Graph shows the normalised pMLC levels (pMLC intensity/actin intensity) by immunostaining in control (Ctl; -dox) and FHL2-knockdown cells (shFHL2; +dox). *N* = 3 independent experiments (*n* = 125 for Ctl, 119 for shFHL2). ****P < 0.0001. (**C**) Western blot of lysates from Ctl and shFHL2 cells. Samples were analysed by immunoblotting for FHL2, pMLC, MLC, and tubulin (loading control). For representative purposes, this blot is also depicted in Fig. EV8A. (**D**) Normalised pMLC intensity (pMLC/MLC intensity) in Ctl and shFHL2 cells from western blots. *N* = 4 independent experiments. **P = 0.0023. (**E**) Traction stress maps a representative Ctl (-dox) and FHL2-knockdown (shFHL2; +dox). Vectors indicate direction and magnitude of the stress. (**F**) Strain energy of each cell (in Joules) for Ctl (-dox) and FHL2-knockdown (shFHL2; +dox) cells shown. *N* = 2 independent experiments (*n* = 64 for Ctl, 75 for shFHL2). ****P < 0.0001. (**G**) Schematic showing that the levels of FHL2 in cells alters actomyosin contractility. Taken together with Figs. 3 and 4, schematic shows that the localisation of FHL2 to stress fibres is important for endothelial mechanoresponses. Scale: (**A**) 50 µm, (**E**) 10 µm. Statistical tests: (**B**, **F**) Unpaired *t* test followed by Mann–Whitney Test, (**D**) Unpaired *t* test. In the box-and-whisker plots in (**B**, **F**), the box extends from the 25th to 75th percentile, the whiskers show minimum and maximum values, and the line within the box represents the median. In (**D**), the whiskers indicate the minimum and maximum values, and the centre line represents the median.

**Figure EV8:**
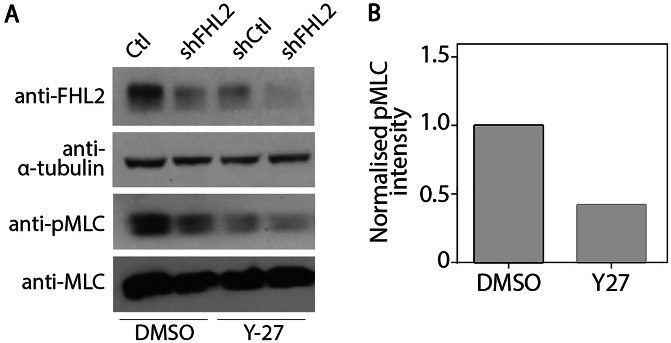
FHL2 and pMLC levels upon ROCK inhibition using Y-27632. (**A**) Western blot of lysates from Ctl and shFHL2 cells (from Fig. 5), with or without Rho-kinase inhibitor (Y-27632 a.k.a. Y-27) are shown. Samples were analysed by immunoblotting for FHL2, pMLC, MLC, and tubulin (loading control). For representative purposes, this blot blot shows the first two lanes that are also depicted in Fig. 5C. (**B**) Normalised pMLC intensity (pMLC/MLC intensity) in cells treated with DMSO Ctl or Y-27. *N* = 1 independent experiment. In the box-and-whisker plots, the box extends from the 25th to 75th percentile, the whiskers show minimum and maximum values, and the line within the box represents the median.

When Rho signalling is triggered, Rho-kinase phosphorylates myosin light chain (pMLC), which in turn, elevates actomyosin contractility. Therefore, in our system, we wondered whether the cell junction defects were caused by heightened actomyosin contractility with FHL2 expression. To this end, we first measured pMLC levels in control and FHL2-knockdown cells. Immunostaining for pMLC and actin revealed a ~30–40% reduction in FHL2-knockdown cells (Fig. 5A,B). These measurements were corroborated with western blotting, where a 30% reduction in pMLC was observed in FHL2-knockdown cells (Figs. 5C,D and EV8A,B).

Next, by employing traction force microscopy (TFM), we computed force generation in endothelial cells plates on fibronectin-coated polyacrylamide gels of 2.8 kPa stiffness. We also calculated the overall strain energy exerted by cells onto these substrates. We observed that FHL2-knockdown resulted in approximately twofold lower strain energies compared to control cells (Fig. 5E,F). These findings underscore the importance of FHL2 in promoting actomyosin contractility through RhoGTPase signalling. Taken together with results from Fig. 4, this also suggests that FHL2-mediated contractility is essential for discontinuous adherens junction morphologies, which is a characteristic hallmark of endothelial dysfunction (Fig. 5G).

### FHL2 promotes actomyosin contractility through crosstalk with the microtubule cytoskeleton

RhoA-mediated contractility is often regulated by Rho guanine exchange factors (RhoGEFs) or RhoGTPase activating proteins (RhoGAPs) (Hodge and Ridley, 2016). In endothelial cells, the RhoGEF, GEF-H1, is one of the most abundantly expressed GEFs and has been implicated widely in mechanotransduction of various cell types (Chang et al, 2008; Krendel et al, 2002; Guilluy et al, 2011; Birukova et al, 2010; Kopf et al, 2020). Thus, our hypothesis was that FHL2-mediated contractility could occur through RhoGTPase signalling, via GEF-H1.

It has been demonstrated that GEF-H1 is inactive when it is directly bound to and sequestered on microtubules; on the other hand, the release of GEF-H1 from microtubules into the cytoplasm is critical for its ability to bind and activate RhoA (Chang et al, 2008; Krendel et al, 2002; Jiu et al, 2017; Meiri et al, 2012). Here, we utilised this localisation of GEF-H1, either on microtubules or in the cytoplasm, as a key readout of its activity and role in tuning RhoA-mediated contractility.

**Figure 6:**
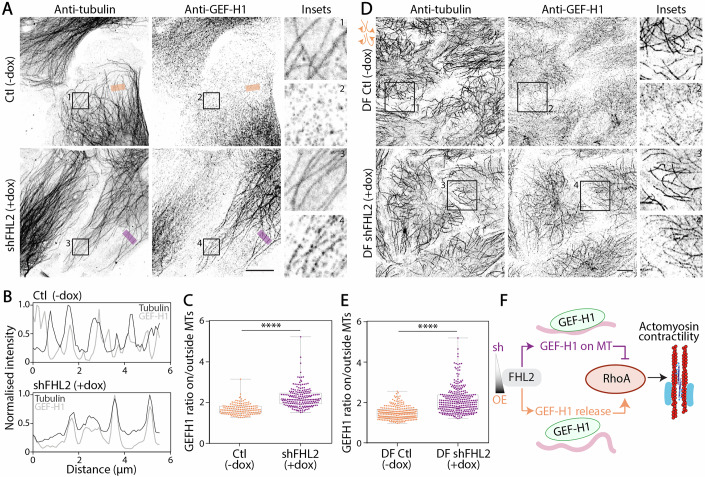
FHL2 promotes the release of GEFH1 from microtubules to the cytoplasm. (**A**) Inverted contrast confocal images of control (Ctl; -dox) and FHL2-knockdown (shFHL2; +dox) TeloHAECs, immunostained for α-tubulin and Rho guanine nucleotide exchange factor, GEF-H1. GEF-H1 sequestered on microtubules renders the GEF inactive, while cytoplasmic localisation (released from microtubules) marks the active pool of GEF-H1 that can activate RhoA. (**B**) Line scans showing GEF-H1 and tubulin intensities from a representative region of a microtubule. (**C**) Graph showing the ratio of GEF-H1 intensity on vs. off microtubules. *N* = 2 independent experiments (*n* = 151 for Ctl, 203 for shFHL2; each dot represents a region of interest; ~2 regions per cell measured). ****P < 0.0001. (**D**) Inverted contrast confocal images of Ctl (-dox) and shFHL2 ( + dox) TeloHAECs under DF, immunostained for α-tubulin and GEF-H1. (**E**) Graph showing the ratio of GEF-H1 intensity on vs. off microtubules. *N* = 3 independent experiments (*n* = 303 for DF Ctl, 344 for DF shFHL2; each dot represents a region of interest; ~1–2 regions per cell measured). (**F**) Schematic showing that FHL2 expression promotes the release of GEF-H1 into the cytoplasm, which can activate RhoA. On the other hand, cells with low FHL2 levels exhibit microtubule-bound inactive GEF-H1. Scale: (**A**) 10 µm, (**D**) 20 µm. Statistical tests: (**C**, **E**) Unpaired *t* test followed by Mann–Whitney test. In the box-and-whisker plots, the box extends from the 25th to 75th percentile, the whiskers show minimum and maximum values, and the line within the box represents the median.

We first tested whether FHL2 alters GEF-H1 localisation. In control cells under static/no flow conditions, GEF-H1 localisation was primarily cytoplasmic (Fig. 6A). We observed high intensity peaks of tubulin, with the lack of GEF-H1 corresponding to these peaks in control cells (Fig. 6B). By contrast, in FHL2-knockdown cells, GEF-H1 peaks are coincident with tubulin intensity peaks, indicating a strong localisation of GEF-H1 to microtubules (Fig. 6A,B).

Next, we quantitatively assessed the localisation of GEF-H1 on a microtubule versus its released form in the cytoplasm (Fig. 6C). In FHL2 knockdown cells, our analysis revealed that the ratio of GEF-H1 on microtubules versus the cytoplasm was higher, in line with our observations showing strong decoration of the microtubule network by GEF-H1 (Fig. 6A,B). This suggests that with FHL2 expression, GEF-H1 is predominantly cytoplasmic, and thus, facilitates downstream Rho signalling.

**Figure EV9:**
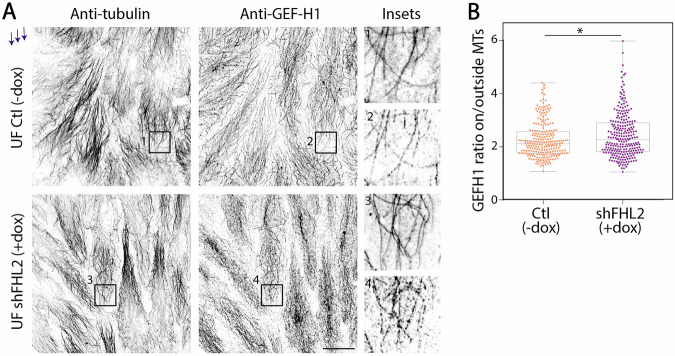
GEFH1 localisation on microtubules in cells under UF. (**A**) Inverted contrast confocal images of control (Ctl; -dox) and FHL2-knockdown (shFHL2; +dox) TeloHAECs under UF, immunostained for α-tubulin and Rho guanine nucleotide exchange factor, GEF-H1. (**B**) Graph showing the ratio of GEF-H1 intensity on vs. off microtubules. *N* = 3 independent experiments (*n* = 237 for UF Ctl, 250 for UF shFHL2; each dot represents a region of interest; ~1–2 regions per cell measured). Scale: (**A**) 20 µm. Statistical tests: (**B**) Unpaired *t* test followed by Mann–Whitney test. *P < 0.05. In the box-and-whisker plots, the box extends from the 25th to 75th percentile, the whiskers show minimum and maximum values, and the line within the box represents the median.

To verify that FHL2-mediated contractility occurs through GEF-H1 release, we tested whether GEF-H1 localisation varies in response to flow and FHL2 expression. In control cells under DF, GEF-H1 is highly cytoplasmic, as shown by the lower microtubule-bound GEF-H1 (Fig. 6D,E). Strikingly, in FHL2 knockdown cells, we observed significant sequestration of GEF-H1 on microtubules, even under DF (Fig. 6D,E). We further observed that under UF, both control and FHL2 knockdown cells have significant GEF-H1 accumulation on the microtubule network, suggesting a high fraction of inactive GEF-H1 (Fig. EV9A,B).

Taken together, we provide a mechanism by which FHL2 drives athero-like phenotypes in endothelial tissues, through the release of GEF-H1 from microtubules, which in turn, is known to promote RhoGTPase-mediated contractility (Fig. 6F).

### FHL2 promotes microtubule dynamics to control GEF-H1 localisation

Given that FHL2 expression is key to tuning GEF-H1 localisation, we investigated the mechanisms by which FHL2 promoted the release of GEF-H1 from microtubules. GEF-H1 release primarily occurs at the cell protrusions and edges through changes in microtubule dynamics and stabilisation (Guilluy et al, 2011; Azoitei et al, 2019; Rafiq et al, 2019; Waterman-Storer and Salmon, 1997; Gupton et al, 2002; Seetharaman et al, 2022). Specifically, when microtubules undergo depolymerisation, GEF-H1 is released into the cytoplasm to drive robust actomyosin contractility in several cell types (Meiri et al, 2012; Chang et al, 2008; Rafiq et al, 2019). Further, microtubule stabilisation through post-translational modifications like acetylation, or microtubule uncoupling from focal adhesions at the cortex through KANK and other cortical microtubule stabilising complex proteins, promotes GEF-H1 activity, resulting in heightened contractility (Rafiq et al, 2019; Seetharaman et al, 2022; Aureille et al, 2024).

**Figure 7:**
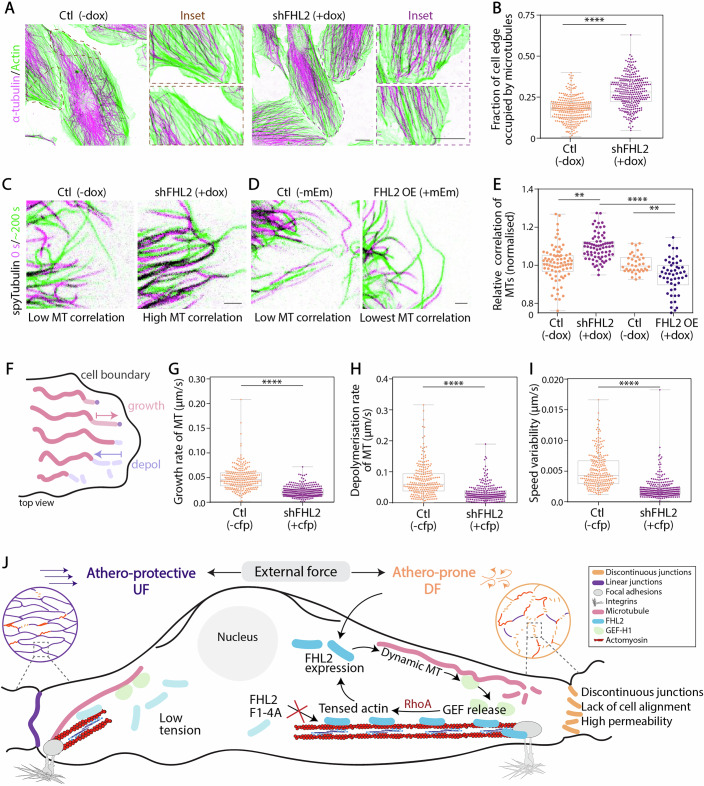
FHL2 augments microtubule dynamics to promote actomyosin contractility. (**A**) Inverted contrast confocal images of TeloHAECs stained for α-tubulin (magenta) and actin (green). Cell protrusion is indicated by the region marked with dotted line, and zoomed-in images of the protrusion are shown. (**B**) Graph shows the fraction of the cell edge occupied by microtubules, by measuring α-tubulin intensity in regions marking the cell protrusions. *N* = 3 independent experiments (*n* = 312 for Ctl, 307 for shFHL2; each dot represents a region; ~1–2 regions per cell measured). ****P < 0.0001. (**C**, **D**) Using spyTubulin labelling in cells imaged over time, microtubule fluctuations between each frame are represented as the correlation index. Inverted contrast images of microtubules in (**C**) Ctl (-dox), shFHL2 (+dox), and (**D**) Ctl (-dox), FHL2 OE (+dox) cells. Two representative frames, approximately 200 s apart are shown in green and magenta. A high correlation index corresponds to low fluctuations or dynamics, while a low correlation index suggests a highly fluctuating microtubule network. (**E**) Graph represents the normalised relative correlation of microtubules over time in Ctl, shFHL2 (+dox), and FHL2 OE (+dox) cells. *N* = 3 independent experiments for Ctl and shFHL2, 5 independent experiments for Ctl and FHL2 OE (*n* = 79 for Ctl, 74 for shFHL2, 37 for Ctl, 57 for FHL2 OE; each dot represents a region in a cell protrusion). **P = 0.0044 for Ctl vs. shFHL2, **P = 0.0043 for Ctl vs. FHL2 OE, ****P < 0.0001 for shFHL2 vs. FHL2 OE. (**F**) Schematic of microtubule growth and depolymerisation in the protrusion of a cell. This was captured by live-imaging microtubules (using spy-Tubulin) with a time interval of ~5 s. (**G**) Graph shows growth rate of microtubules in Ctl (cfp-negative) and shFHL2 (cfp-positive) cells. Microtubule growth rate is defined by the average length increase per time for all frames during periods of growth. *N* = 2 independent experiments (*n* = 246 for Ctl, 308 for shFHL2; each dot represents a microtubule tip growth event). ****P < 0.0001. (**H**) Graph shows depolymerisation rate of microtubules in Ctl (cfp-negative) and shFHL2 (cfp-positive) cells. Microtubule depolymerisation rate is defined by the average length increase per time for all frames during periods of shrinkage. *N* = 2 independent experiments (*n* = 240 for Ctl, 261 for shFHL2; each dot represents a microtubule tip depolymerisation event). ****P < 0.0001. (**I**) Graph shows speed variability of microtubules in Ctl (cfp-negative) and shFHL2 (cfp-positive) cells. Speed variability is defined by the standard deviation of the absolute values of displacement between frames. *N* = 2 independent experiments (*n* = 246 for Ctl, 315 for shFHL2; each dot represents a microtubule tip depolymerisation event). ****P < 0.0001. (**J**) Model of FHL2-mediated endothelial mechanoresponses. Upregulation of FHL2 and its binding to stress fibres in response to DF results in the release of GEF-H1 from microtubules into the cytosol, where it can activate RhoA. In turn, cells exhibit hypercontractility, where stress fibres build tension and pull adherens junctions to cause breakages and discontinuity. Subsequently, with discontinuous focal adherens junction formation, endothelial tissues with high FHL2 have increased permeability and tissue dysfunction. Scale: (**A**) 10 µm, (**H**) 2 µm. Statistical tests: (**B**, **G**–**I**) Student’s *t* test followed by Mann–Whitney test, (**E**) one-way ANOVA followed by Tukey’s multiple comparisons test. In the box-and-whisker plots, the box extends from the 25th to 75th percentile, the whiskers show minimum and maximum values, and the line within the box represents the median.

To probe the mechanism of FHL2-mediated GEF-H1 release, we tested whether FHL2 expression alters microtubule organisation at the cell periphery. In this case, we selected regions in the cell periphery and quantified the fraction of the region occupied by microtubules (Fig. 7A). In FHL2 knockdown cells, we observed that microtubules extended to the cell periphery, with a significantly higher fraction of the cell protrusion occupied by the microtubule network; control cells, on the other hand, were marked by fewer microtubules reaching the cell periphery (Fig. 7A,B). This strongly suggests that FHL2 prevents the microtubule network from reaching the periphery, possibly by promoting microtubule dynamics or depolymerisation close to the edge.

As GEF-H1 release occurs through dynamic microtubule rearrangements, we then investigated whether tuning FHL2 levels influenced microtubule network fluctuations at the cell edge. By measuring the relative correlation between microtubules over time, we found that the microtubule network, upon FHL2 knockdown, displayed fewer fluctuations or variations compared to control cells. This is represented by the higher correlation seen between microtubules across multiple frames of the FHL2 knockdown movie (Fig. 7C,E; Movie EV1). Conversely, with higher FHL2 levels in the overexpression conditions, we observed a lower microtubule correlation over time, depicting a more dynamically fluctuating microtubule network (Fig. 7D,E; Movie EV2).

Next, by live imaging microtubules (using Spy-tubulin) and FHL2 (using FHL2-OE mEmerald), we observed that FHL2-decorated actomyosin stress fibres restrict the penetration of microtubules through them, thus, dynamically regulating the fraction of microtubules reaching the cell periphery (Movie EV3). This is in line with our findings from Fig. 7A that fewer microtubules ventured to the periphery in cells with higher FHL2 levels (control, FHL2 OE; Movie EV2).

Finally, as it is known that GEF-H1 release is mediated by microtubule dynamics, we quantitatively characterised the effects of FHL2 expression on microtubule growth and depolymerisation rates as well as the variability in the length of microtubules as they dynamically remodelled over time. By tracking individual microtubules from start to end time points in FHL2 knockdown cells, we obtained trajectories/paths to represent the growth and depolymerisation rates of microtubules. Strikingly, we found that FHL2 knockdown (cfp-positive cells) resulted in significantly slower microtubule growth rate as compared to control conditions (cfp-negative cells; Fig. 7F,G). On the other hand, microtubule depolymerisation rates were also significantly lower in FHL2 knockdown cells (Fig. 7F,H). Furthermore, our analysis revealed that microtubules in FHL2 knockdown exhibited steady growth and shrinkage as depicted by the low speed variability as compared to control cells (Fig. 7I). These results strongly suggest that in the presence of FHL2, microtubules fail to reach the periphery of the cell, and the growth and depolymerisation of these microtubules occurs at a much faster rate.

Altogether, our findings depict two interlinked mechanisms by which FHL2 promotes contractility through GEF-H1 release (Fig. 7J): (1) FHL2-decorated actomyosin bundles in control cells restrict microtubules from penetrating into the periphery, and (2) FHL2 modulates microtubule dynamics, especially depolymerisation, which causes fewer microtubules to enter the protrusion. In both cases, our results are in line with other works showing that microtubule depolymerisation, restriction, and destabilisation leads to GEF-H1 release and heightened contractility (Rafiq et al, 2019; Seetharaman et al, 2022; Aureille et al, 2024), as seen in FHL2 expressing cells.

Overall, we demonstrate that in response to athero-prone DF, FHL2 expression promotes actomyosin contractility through dynamic microtubule-based GEF-H1 signalling. Further, our work highlights how stress fibre-binding mechanosensitivity of FHL2 tunes endothelial tissue phenotypes and function (Fig. 7J).

## Discussion

We uncover a positive mechanochemical feedback mechanism between transcription and subcellular FHL2 localisation to tune actomyosin contractility via GEF-H1 (Fig. 7J). This positive feedback is limited by FHL2 expression to modulate the extent of mechanosensitive Rho signalling and contractility. Over longer time scales, transcriptional regulation of FHL2 mediates hypercontractility of endothelial tissues subjected to athero-prone flow and is the origin of morphological hallmarks of endothelial dysfunction (Fig. 6J). Here, we show how the force-sensitivity of FHL2 regulate endothelial mechanotransduction from the subcellular to tissue-scale morphology and function, in response to one mechanical cue seen in arteries, i.e., flow. Further work is needed to explore how this could be a general phenomenon in response to other external forces (e.g., matrix stiffness, stretch or curvature).

Our results demonstrate that the transcriptional regulation of FHL2 occurs as a very early mechanoresponse to changes in flow profiles and precedes the atherosclerotic disease phenotype. Several studies have focused on the transcriptional changes in healthy vs. atherosclerosis arteries, where transcription factors including KLF2, NF-kB and c-Jun have been largely implicated in disease progression. In addition to known transcription factors and cardiovascular genes previously discovered, we find that over 31 LIM domain proteins, including FHL2, are significantly altered during flow-sensing in endothelial tissues. It is noteworthy that major endothelial transcription factors like KLF2 and KLF4, similar to LIM domain proteins, also contain Zinc finger motifs and bind DNA and are crucial for endothelial tissue function (Velyvis and Qin, 2013; Turpaev, 2020). Taken together, this suggests that the LIM domain protein family might be crucial for differentially contributing to mechanotransduction in various other cardiovascular diseases that are primarily mediated by changes in flow, either through their transcriptional regulation or interactions through Zn-finger motifs. Moreover, it is possible that FHL2 primes specific regions in the artery to endothelial dysfunction and subsequent atherosclerotic plaque formation. In this context, it would also be interesting to explore the extent to which perturbed tissue phenotypes are reversible by tuning the transcription of mechanosensitive genes, for instance, via FHL2 and its known regulators MRTF/SRF (Philippar et al, 2004; Hinson et al, 2008). Furthermore, the FHL2 expression varies across tissue types suggesting that the protein is differentially regulated to maintain specific functionality. For instance, FHL2 expression is extremely high in heart tissues, especially in highly contractile cardiomyocytes where FHL2 localises strongly to Z-discs, suggesting a potential role in mechanical stabilisation of muscle cells (Samson et al, 2004; Tran et al, 2016a). Thus, the distinct expression levels as well as localisation indicate that FHL2 could be a useful biomarker of the state of endothelial tissues, particularly as it is a crucial regulator of endothelial mechanoresponses.

We identify a function of the force-activated binding of FHL2 to stress fibres. Although the mechanosensitive binding of FHL2 to stress fibres has been well described (Winkelman et al, 2020; Sun et al, 2020; Nakazawa et al, 2016), the function of this binding was unknown. We show that the force-sensitive binding of FHL2 to actomyosin stress fibres promotes RhoA activation via GEF-H1 that, in turn, enhances contractility. Recent work suggests that nuclear-cytoplasmic mechanotransduction plays a key role in regulating endothelial contractility through proteins of the LINC complex (Buglak et al, 2023). Along these lines, we speculate that the concentration of cytoplasmic and stress fibre-bound FHL2, possibly through its nuclear-to-cytoplasmic shuttling or transcriptional status (Nakazawa et al, 2016; Philippar et al, 2004), can serve as a way to tune this positive mechanochemical feedback. Thus, this provides a pathway by which overall FHL2 transcriptional control coordinated with local subcellular mechanotransduction regulate RhoGTPase signalling.

Our results strongly suggest that FHL2 mediates force-dependent actomyosin-microtubule crosstalk. We show that FHL2-enriched stress fibres promote microtubule dynamics and prevent the microtubule network from reaching the cortex. In this case, local microtubule depolymerisation, instability at the cortex, or uncoupling from integrin-mediated focal adhesions can cause GEF-H1 release into the cytoplasm (Chang et al, 2008; Guilluy et al, 2011; Rafiq et al, 2019). For instance, focal adhesion proteins like KANK, CLASP, and αTAT1, allow targeting and stabilisation of microtubules at the cortex (Rafiq et al, 2019; Seetharaman et al, 2022; Aureille et al, 2024; Stehbens et al, 2014; Bouchet et al, 2016; Ju et al, 2024) thus serving as a local hotspot for changes in GEF-H1 release, actomyosin contractility, and adhesion turnover (Aureille et al, 2024). Similarly, it is possible that FHL2, in addition to its force-sensitive binding to actin, interacts with such focal adhesion components or microtubule-actin crosslinking factors to tune GEF-H1 localisation and activity, microtubule acetylation, or targeting/stabilisation at the cortex (Callow et al, 2005; Zenke et al, 2004; Tran et al, 2016b; Aureille et al, 2024). Furthermore, in the context of endothelial tissue function, our findings are in agreement with previous work showing that activated GEF-H1 and microtubule depolymerisation drive vascular permeability in both in vitro and in vivo models (Zhou et al, 2013; Birukova et al, 2010; Kratzer et al, 2012; Buglak et al, 2023; Malek and Izumo, 1996). Although we provide mechanistic insight into how FHL2 drives athero-like phenotypes in endothelial monolayers, future work in in vivo models of atherosclerosis can reveal whether FHL2-mediated contractility and actomyosin-microtubule crosstalk are important for disease progression.

Endothelial function is a multi-scale process that requires coordination of temporal events that control and maintain health. For instance, tissue-scale permeability is a function of cellular mechanotransduction which occurs at the junctions, and different adherens junction morphologies contribute to this mechanoresponse. In various tissue types, adherens junctions are mechanosensitive and are coupled tightly to the actomyosin network through force-dependent recruitment of proteins such as vinculin or α-catenin (Friedrich et al, 2019; Liu et al, 2010; Wójciak-Stothard et al, 2001; Jou et al, 1998; Sahai and Marshall, 2002; Tzima et al, 2005; Yonemura et al, 2010; Conway et al, 2013; Schulte et al, 2011; Frye et al, 2015; Kotini et al, 2022). In endothelial tissues, discontinuous focal adherens junctions are described as points of high tension (Oldenburg et al, 2015; Huveneers et al, 2012; Millán et al, 2010). Our work suggests a mechanism where FHL2 binding to stress fibres increases force generation to drive adherens junction remodelling. It is tempting to speculate that, through its effects on contractility, FHL2 directly or indirectly alters the activity of a key mechanosensitive protein at adherens junctions, resulting in endothelial physiology or dysfunction as described in this study. Using complex 3D and in vivo models, it would also be interesting to explore the physiological role of FHL2 in mediating endothelial force-adaptation and permeability during angiogenesis or inflammation. Finally, considering recent work showing that nuclear mechanotransduction through SUN1 loss drives discontinuous adherens junctions and contractility through GEF-H1 without any changes in transcriptional profiles (Buglak et al, 2023), it would be interesting to explore whether and how the transcriptional regulation of FHL2 in the nucleus mediates adherens junction remodelling, and whether this can be tuned to engineer endothelial physiology. Overall, our work bridges subcellular mechanochemical feedback and transcriptional regulation to tissue-scale endothelial mechanoresponses and function.

## Methods

**Table:** Reagents and tools table

Reagent/resource	Reference or source	Identifier or catalog number
**Experimental models**		
TeloHAEC Cell Line	ATCC	#CRL-4052
C57BL/6 mice	Jackson Laboratory	
**Recombinant DNA**		
**Antibodies**		
FHL2	Sigma	HPA006028
VE-Cadherin	Santa Cruz	SC-9989
GEF-H1	Abcam	ab155785
alpha-tubulin Y1/2	Abcam	ab6160
pMLC	Cell Signaling	3671S
GAPDH	Sigma	G9545
**Oligonucleotides and other sequence-based reagents**		
AAVS1-flag-gfp-fwd	IDT	AGAATTCGCGGCCGCACGCGATGGGTGGCGGTGGCTC
cfp shrna fwd	IDT	CGTAAAGAATTCGCGGCCGCACGCGATGGTGAGCAAGGGCGAGGAGC
cfp shrna hom rev	IDT	CTTGTACAGCTCGTCCATGCCG
shrna yfp fwd	IDT	TCGGCATGGACGAGCTGTACAAGTAAAGCTTCTAGACCCGGG
5 mir rev	IDT	CGCTCACTGTCAACAGC
3 mir fwd	IDT	TGCCTACTGCCTCGG
cfp shrna 3p rev	IDT	TCCAGAGGTTGATTGTCGAGAGATCTCGGTAGAATTCCATATGACGCGTAAAGTG
FHL2 shRNA 1	IDT	CGAGACTTTCTTCTAGTGCTTT (Nakazawa et al, 2016)
FHL2 shRNA 2	IDT	AAACGAATCTCTCTTTGGCAAG (Wu et al, 2013)
hGAPDH for	IDT	TGC ACC ACC AAC TGC TTA GC
hGAPDH rev	IDT	GGC ATG GAC TGT GGT CAT GAG
hACTB for	IDT	TCC CTG GAG AAG AGC TAC GA
hACTB rev	IDT	AGG AAG GAA GGC TGG AAG AG
hUbb for	IDT	ATT TAG GGG CGG TTG GCT TT
hUbb rev	IDT	TGC ATT TTG ACC TGT TAG CGG
mACTB for	IDT	GAT CAA GAT CAT TGC TCC TCC TG
mACTB rev	IDT	AGG GTG TAA AAC GCA GCT CA
mUbb for	IDT	AGT GAC GAG AGG CTT TGT CC
mUbb rev	IDT	CGA AGA TCT GCA TTT TGA CCT GT
hFHL2 for	IDT	GCC GTG AGT ACC TCC AAC C
hFHL2 rev	IDT	GGC TTT TCA GCA ACC CCA AA
mFHL2 for	IDT	TTC CAT ACT GCC TGA CCT GC
mFHL2 rev	IDT	CAC CCA GAC CAC TAA TGG GG
**Chemicals, enzymes and other reagents**		
EGM-2 medium	Lonza	CC-3156 & CC-4176
Mycoplasma kit	InvivoGen MycoStrip^TM^	rep-mys-10
Spytubulin	Cytoskeleton Inc.	CY-SC203
Spyfastact	Cytoskeleton Inc.	NC2204057
CellMask Orange	Invitrogen	C10045
NucleoSpin RNA kit	Macherey-Nagel	740955
QIAzol lysis reagent	QIAGEN	79306
GenElute Mammalian Total RNA Miniprep Kit	Sigma	RTN70
High-Capacity cDNA Reverse Transcription Kit	ThermoFisher	4374966
Pierce ECL western blot substrate	ThermoFisher	32106
Transwell plates	Corning	734-1579
FITC-dextran	Sigma	FD4-100MG
40% acrylamide	Bio-Rad	1610146
2% bis-acrylamide	Bio-Rad	1610142
**Software**		
Fiji		
GraphPad Prism		
Adobe Illustrator		
MATLAB		
**Other**		

### Cell culture, treatments and transfection

Human aortic endothelial cells (teloHAECs) were provided by Yun Fang (University of Chicago), who purchased them from ATCC (#CRL-4052). Cells were grown at 37 °C with 5%CO_2_ using EGM-2 medium supplemented with SingleQuots from Lonza (CC-3156 & CC-4176). Cells were passaged using 0.25% trypsin EDTA every 2–3 days. Cells were tested for mycoplasma every 6 months or as necessary (no mycoplasma detected) using InvivoGen MycoStrip^TM^ (rep-mys-10) and spyDNA/Hoechst staining. Cells were electroporated using the Neon™ Transfection System, with 1 pulse at 1350 V for 30 ms, and plated on appropriate supports, coverslips or culture dishes as needed. The growth medium was replaced 8 or 24 h post-transfection. For treatment with Rho-kinase (ROCK) inhibitor Y-27632, cells were treated with 10 µM for 4 h. To visualize microtubules and actin during live-cell imaging, cells were incubated with Spytubulin (CY-SC203; 1:2000) and Spyfastact (NC2204057; 1:2000) for 1-2 h, and fresh growth medium was replaced prior to imaging. For traction force experiments, cells were incubated with CellMask Orange (Invitrogen, Cat# C10045; 1:5000) for 20 min, washed once and replaced with fresh growth medium prior to imaging.

### Cell line generation

FHL2 overexpression and knockdown teloHAEC cell lines were made by CRISPR-mediated knock-in to the AAVS1 locus of tet-inducible FHL2-mEmerald or a tet inducible mir30a shRNA containing two previously published sequences targeting FHL2 (see below). These sequences were ordered from IDT and cloned into a previously published vector (pMK243) with homology arms targeting the human AAVS1 locus and co-expression of tet-on3g. mir30a based shRNA was designed as previously described (Chang et al, 2013). Each plasmid was co-transfected with a plasmid encoding CAS9 and a sgRNA targeting the AAVS1 locus (AAVS1 T2 CRIPR in pX330) at a ratio of 1:1 using the Neon transfection system. All constructs were sequenced at the University of Chicago Genomics core and verified prior to use. One week after transfection, cells were induced with doxycycline and sorted for expression of GFP or CFP. Cells were induced and sorted as needed before use for experiments. For flow experiments, cells were re-sorted to ensure a good population of positive GFP or CFP cells. FHL2 shRNA sequence is as follows: AAACGAATCTCTCTTTGGCAAG (Wu et al, 2013). Primers used in this study are listed in the Reagents Table.

Tet-inducible gene expression in these cell lines were carried out with 100–200 ng/ml doxycycline in all indicated experiments (+dox). For FHL2 overexpression, cells were treated with dox for at least 24 h, and for FHL2 knockdown cells, cells were treated with dox for a minimum of 4 days before that start of experimentation. In both cases, fresh doxycycline was given each day of incubation.

### Flow experiments

Cells were cultured in 6-well plates with EGM-2 medium supplemented with 4% dextran (Sigma-Aldrich, St. Louis, MO, 31392). Hemodynamic flows were applied to using a 1o tapered stainless steel motorised cone (UMD-17 (Arcus20 Technology, Livermore, CA). The rotation of the cone captures healthy unidirectional flow (UF) seen in athero-protective distal internal carotid artery, while the disturbed flow (DF) mimics athero-susceptible human carotid artery (Dai et al, 2004). During flow application to cells, the flow device was placed at 37 °C with 5% CO_2_.

### RNAsequencing

Cells subjected to UF and DF were lysed, and total RNA was collected using a NucleoSpin RNA kit (Macherey-Nagel, #740955). Two technical replicates for each condition were pooled together to obtain an experimental replicate. The RNA samples from three separate experimental replicates were submitted to the University of Chicago Genomics Facility. The quality and quantity of RNA were assessed using the Agilent bio-analyzer (RNA Integrity Number from 9.9 to 10). Using a TruSEQ mRNA RNA-SEQ library preparation protocol (Illumina provided), strand-specific RNA-SEQ libraries were prepared. Libraries were then sequenced using Illumina NovaSEQ6000 (Illumina provided reagents and protocols) with ~60 M PE reads/sample. The reads were aligned to the Homo Sapiens genome hg19/GCh37 reference by pseudoalignment using Kallisto 0.46.1. All raw files and counts have been uploaded to Annotare (ArrayExpress E-MTAB-16095) and will be made available prior to publication.

#### Differential gene expression analysis

DESeq2 (version 1.40.2) in R software (version 4.3.1) was used to normalize read counts per transcript and perform differential gene expression analysis. DESeq2 normalizes the data according to the following steps: 1. calculation of a fictive reference sample defined as the geometric mean for each gene across all samples, 2. counts for each gene for each sample are divided by this reference value, 3. size factors are estimated by calculation of the median of these ratios across samples, 4. raw gene counts for each sample are divided by the sample size factor to obtain normalized counts. This method normalization accounts for library size differences and samples bias. DESeq2 uses normalized counts to calculate log2fold changes and *P* values using the Wald test. The Benjamini–Hochberg correction was performed to obtain *P* values adjusted for multiple testing. Genes were defined as significantly differentially expressed at an adjusted *P* value of <0.05.

#### Gene set enrichment analysis

Simple gene set enrichment analysis was performed in R software using clusterProfiler 4.8.3 (Wu et al, 2021) and version 7.5.1 of the molecular signature database (Subramanian et al, 2005). Differentially expressed genes were tested for overrepresentation in Gene Ontology resource (release date 2023-01-01) clusters ((Ashburner et al, 2000) and the Gene Ontology Knowledge Base, 2023) of molecular function, cellular compartment and biological processes. The HGNC gene nomenclature committee annotation of genes (https://www.genenames.org/) was used as an additional database to test in what gene families selected genes were overrepresented using clusterProfiler.

### Partial carotid ligation surgery

All mouse procedures were approved by the Institutional Animal Care and Use Committee (IACUC ACUP#72567; PI Y. Fang) of The University of Chicago. Partial carotid artery ligation was surgically performed to introduce acute disturbed flow activating endothelial cells in the left carotid artery. Briefly, male 7–9 weeks old C57BL/6 mice (The Jackson Laboratory) were anesthetized by intraperitoneal injection of ketamine (100 mg/kg) and xylazine (10 mg/kg) mixed solution. A ventral midline incision (~5 mm) in the neck was opened by a tiny scissor (F.S.T 14090-11). The left carotid artery was exposed by blunt dissection under microscopy. Then, the left external carotid, internal carotid, and occipital artery were ligated with 6-0 silk suture while the superior thyroid artery remains intact. After that, the incision was closed with Nylon monofilament suture (Air-Tite products) and the cut was smeared with Betadine to prevent infection. 48 hours after ligation, the animals were euthanized, and both carotid arteries were harvested after saline perfusion via the left ventricle after severing the inferior vena cava. After euthanasia and saline perfusion, carotid arteries were isolated en bloc. Carotid arteries were fixed in 4% paraformaldehyde, sequentially immersed in 30% sucrose and optimal cutting temperature compound (OCT):30% sucrose (1:1) mixed solution, and then embedded in OCT. Frozen-embedded samples were sectioned in 5- to 8-μm thickness. Sections were then immunostained as described later in the “Methods” Section.

#### Intimal RNA isolation from carotid arteries

After careful isolation, the carotid lumen was quickly flushed with 350 μl of QIAzol lysis reagent (QIAGEN, MD, USA) using a 29-gauge insulin syringe, and the elute was collected in a microfuge tube. The elute was further applied for intimal RNA isolation. Total RNA was reverse transcribed with Maxima First Strand cDNA Synthesis Kit (Thermo Fisher Scientific, MA, USA) and SYBR Green qRT-PCR was performed.

### Quantitative real-time PCR (qPCR)

Total RNA was extracted with GenElute Mammalian Total RNA Miniprep Kit (Sigma). 0.2 μg mRNA was reverse-transcribed using High-Capacity cDNA Reverse Transcription Kit (ThermoFisher). The cDNA was amplified by LightCycler 480 II (Roche) system using a SYBR Green probe. Absolute quantification of the gene of interest was normalized to the geometric mean of β-actin, GAPDH and Ubiquitin. Primers used in this study are listed in the Reagents Table.

### Immunostaining

Endothelial cells were washed with 1× PBS gently, and subsequently fixed using one of the following three methods: (1) warm 4% PFA for 10 min at 37 °C, followed by 0.2% Triton X-100, (2) ice-cold methanol for 2–3 min at −20 °C to stain for microtubules and GEF-H1, (3) 4% warm PFA + 0.2% glutaraldehyde + 0.25% Triton X-100 for 10 min at 37 °C. In the case of method 3 using PFA and glutaraldehyde, the free aldehyde groups are quenched with a 1 mg/ml sodium borohydride freshly added to cytoskeletal buffer (10 mM MES or MOPS, 150 mM NaCl, 5 mM ethylene glycol-bis(β-aminoethyl ether)-N,N,N′,N′-tetraacetic acid (EGTA), 5 mM MgCl_2_ and 5 mM glucose, pH 6.1) and placed on ice or at 4 °C for 10 min. After fixation, a 5% bovine serum albumin solution in PBS was used for blocking at room temperature for 1 h. The same BSA solution was used to prepare the primary and secondary antibody solutions, which were incubated for 1 h each, with PBS washes in between the incubation periods. In the case of flow experiments, primary antibody incubation was carried out at 4 °C overnight. Coverslips were mounted on glass slides using Prolong Gold Antifade Mounting medium.

#### Antibodies used in this study

FHL2 (rabbit, Sigma, HPA006028, 1:100), VE-Cadherin (mouse, Santa Cruz, SC-9989, 1:2000) pMLC (rabbit, Cell Signaling, 3671S, 1:1000), GEF-H1 (rabbit, Abcam, ab155785, 1:500), alpha-tubulin (Y1/2, rat, Abcam, ab6160). Secondary antibodies were purchased from Jackson Antibodies.

### Western blotting

Cells were lysed using Laemmli buffer (60 mM Tris-HCl pH 6.8, 10% glycerol, 2% sodium dodecyl sulfate (SDS) and 50 mM dithiothreitol (DTT)), with the addition of anti-protease and phosphatase inhibitors. Cell lysates samples were boiled for 5 min at 95 °C before freezing at −20 °C or loading on to PAA gels. Proteins were transferred onto a nitrocellulose membrane, at 100 V for 1 h. Membranes were blocked with 5% milk in Tris-buffered saline, 0.1% Tween 20 detergent (TBST) for 1 h, followed by primary antibody (diluted in a solution of 5% milk in TBST) overnight at 4 °C. Membranes were then washed with TBST and incubated with horseradish peroxidase (HRP)-conjugated secondary antibody (diluted in 5% milk in TBST) for 1 h at room temperature. Bands were revealed using ECL chemoluminescent substrate (Thermo Fischer Pierce ECL western blot substrate).

#### Antibodies used in this study

GAPDH (rabbit, Sigma G9545, 1:2000), other antibodies were the same as for immunostaining and are listed above. Secondary antibodies used were anti-rat-HRP (Thermo Fischer, A10549, 1:10,000), anti-rabbit HRP (Cell Signaling, 7074S, 1:10,000), anti-mouse HRP (Cell Signaling, 7076, 1:10,000).

### Permeability assay

Cells were plated to confluency in 0.4 µm 12-well transwell plates (Corning, #734-1579). Total volume in the top chamber was 200 µl, and 1.5 ml of media was added to the bottom chamber. Cells were cultured on transwell inserts for 24–48 h until they formed a confluent monolayer. The media from the top chamber was carefully aspirated and replaced with 200 µl media containing 10 µl/ml FITC-dextran (Sigma, FD4-100MG) of 4 kDa molecular weight. Media from the bottom chamber was replaced with fresh media. Cells in transwells were incubated for 8 hours in 37 °C with 5% CO_2_. After 8 h, media from the top and bottom chambers were collected in a centrifuge tube for processing. 50 µl each from the top and bottom chambers were transferred into separate wells in a 96-well plate. A standard curve with FITC-dextran was created and the fluorescence was measured with an excitation wavelength of 488 nm and emission of 520 nm, using a plate reader. The data was analysed according to previous literature (Miteva et al, 2010). Briefly, using the volume of media in each chamber, time, surface area/well, and the measured fluorescence intensity in each condition, we calculated the fold change in permeability after calculation of the absolute concentration of FITC in the top and bottom chambers.

### Traction force experiments

Coverslips were silanised for 10 min in a solution of 1% (v/v) 3-(trimethoxysilyl)propyl methacrylate and 1% (v/v) acetic acid in ethanol, and washed twice with absolute ethanol and dried on Whatmann paper. PAA gels of 8.4 kPa Young’s Modulus were prepared using 3.125 ml 40% acrylamide (Bio-Rad Laboratories, Hercules, CA), 0.833 ml 2% bis-acrylamide (Bio-Rad), and 1.042 ml water. To a 500 µl aliquot of this gel mixture, 5 µl of 110-nm sulfate-modified fluorescent microspheres (Invitrogen, Carlsbad, CA). Following this, 2.5 µl of 10% ammonium persulfate (APS) and 0.25 µl of tetramethylethylenediamine (TEMED) were added and the gel solution was mixed well. A 14 µl drop of the solution was placed on each silanised coverslip (22 × 22 mm) and immediately a slide or 18 × 18 mm coverslip was placed gently over the solution. The solution was allowed to polymerize for 30 min at room temperature. Once the gel polymerised, Milli-Q water was added over the coverslips, and the top glass was detached using a scalpel blade. The polymerized gel was then activated for 5 min under ultraviolet light with Sulpho-SANPAH and washed with 10 mM HEPES (4-(2-hydroxyethyl)-1-piperazineethanesulfonic acid) twice. Gels were coated with a fibronectin solution in PBS immediately after washing and placed in the 37 °C incubator. Excess matrix protein was washed with PBS and 25,000 cells were plated per coverslip and allowed to adhere overnight. Right before the traction force experiments, cells were stained with CellMask Orange to visualise the cells and membranes and rinsed two times with warm PBS. Samples were then mounted on a Chamlide Magnetic Chamber and placed in a stage-top incubator maintained at 37 °C and 5% CO_2_. Z-stacks of the fluorescent beads and cell mask were imaged.

### Imaging and image analysis

Images were acquired from various fields of view that are randomly chosen, using channels like CFP nucleus/actin as standard reference staining of cells. This allowed us to acquire images covering a wide range of intensities, without choosing a specific signal range. Further, for some imaging experiments, when possible, one condition was first imaged blinded, without checking what treatment it belonged to, followed by the second condition. All imaging settings were maintained constant between conditions that are compared directly. ImageJ and MATLAB were used for image analysis. All codes are publicly available on GitHub (https://github.com/shailaja-seetharaman/Paper_draft_codes) or provided upon request from authors.

#### Fluorescence microscopy

Flow and traction force experiments were imaged on a Nikon spinning disc confocal microscope (Nikon TI-E, Nikon, Tokyo, Japan). Images were acquired on Andor Zyla 4.2 CMOS camera (Andor Technology, Belfast, UK), using 642, 561, and 488 lasers. All other imaging was done using a point scanning confocal microscope (Zeiss Airyscan LS980), equipped with laser lines at 405, 491, 561, and 642.

#### Cell alignment

Brightfield images of in vitro endothelial tissues under flow were acquired using a tissue culture microscope. Each frame containing several hundred cells were divided into 2 or 4 images for measuring orientation in small fields of view. The ‘local gradient orientation’ function in Fiji was used to analyse cell alignment. Briefly, a ‘local gradient orientation’ derived by using a 5 × 5 Sobel filter is then used for the histogram. The flow alignment angle (o) is the direction angle which represents the centre of the Gaussian. This has been rescaled such that the peak at angle 0 represents regions aligned with the flow.

#### Junction analysis in flow experiments

Cells were immunostained for Actin and VE-Cadherin and imaged on using a spinning disc confocal microscope as described above. Using a custom MATLAB script, cell junctions were manually annotated into 3 classes: (1) linear, (2) focal adherens, (3) reticular, based on VE-Cadherin morphologies as described in the main text. Having annotated junction morphologies, the fraction of the junction perimeter occupied by each type of junction was calculated. Cells whose boundaries did not fit in a frame or were difficult to annotate for several reasons (including weak/bad immunostaining, highly overlapping cells, dividing or dead cells) were excluded from analysis.

#### Aspect ratio of cells

Using cell boundaries, we also measured the aspect ratio of each cell (ratio of the major to the minor axis of cells), which depicts how elongated or circular a cell is. A higher aspect ratio value represents increased cell elongation. Furthermore, the mean intensities of actin and FHL2 per cell were measured as needed.

#### FHL2 intensity in cells

The mean FHL2 intensity in each cell was normalised to the respective actin intensity. For FHL2 on stress fibres, regions on fibres were highlighted and the intensity of FHL2 and actin were measured within the region (on a stress fibre). For western blotting, FHL2 signal was normalised to the loading control and the experimental mean of the respective controls.

#### FHL2 expression by western blotting

In western blots for mEmerald-FHL2 and mEmerald-FHL2-F1-4A (Figs. EV2C and EV7B), anti-FHL2 blotting revealed two bands marking endogenous FHL2 (~32 kDa) and overexpressed tagged FHL2 (~60 kDa). The sum of the intensities of these two bands was measured to represent the total FHL2 protein expression in cells, as compared to the Ctl cells expressing only the endogenous FHL2 protein.

#### k-means clustering

From measurements of cell junction fractions (linear, reticular, and focal adherens junctions) and cell aspect ratio, cells were clustered using a k-means algorithm on MATLAB using squared Euclidean distance, with k = 2. These distinctly represent UF-like and DF-like clusters as they were matched with the actual experimental condition shown in Fig. EV3B. Cells from all conditions are partitioned into two distinct clusters based on their similarities to one another and to the centroids. Distances of each point to the centroid were also depicted.

#### Linescan intensity profiles

For Fig. 2F, using background (100) subtracted intensities of FHL2 and actin, the FHL2 intensity was normalized to the actin intensity of each cell. For linescans in Fig. 2G, we measured the intensity profile of FHL2 and actin across a line drawn perpendicular to a few actomyosin stress fibres (~3–5 fibres). The FHL2 and actin intensities were normalized such that the max intensity peak is set to 1. In the cells where line scans were drawn, FHL2 intensity in the entire cell was measured, and was around 6.5 times lower in the case of UF cell. Thus, to accurately represent the intensity variations of FHL2 between the conditions as well, FHL2 intensity in the UF condition was divided by 6.5. For Fig. 6B, GEF-H1 and actin intensities were measured across a line spanning few microtubules (~4–6 microtubules). To account for variations in intensities across conditions, the GEF-H1 and tubulin intensities were normalized to the maximum intensity measured for each channel, i.e., the maximum intensity of GEF-H1 and tubulin were both set to 1.

#### Phosphorylated myosin light chain (pMLC) levels

The mean intensity of sparse cells and endothelial monolayers stained for pMLC Serine 19 (S19) residue were measured and normalized to the mean actin intensity of each cell to account for variability across experimental conditions. For western blotting, pMLC signal was normalised to the total MLC levels, and further normalised the experimental mean of the respective controls.

#### Traction force measurements

Traction forces and strain energies were analysed using a custom-designed macro in Fiji based on previous studies (Martiel et al, 2015; Seetharaman et al, 2022). Z-stacks of beads before and after cell trypsinization were selected and aligned using the Align Slices in the Stack plugin in Fiji (a normalized cross-correlation algorithm). The bead displacements were computed from bead movements using particle image velocimetry (PIV). PIV analysis parameters were three interrogation windows of 128, 64 and 32 pixels with a correlation of 0.60. Using Fourier transform traction cytometry, a Young’s modulus of 8.4 kPa, a regularization factor of 10^−9^ and a Poisson ratio of 0.5, traction forces were calculated from the bead displacement fields.

#### GEF-H1 localisation

By drawing a small region on the edge of a cell protrusion where individual microtubules can be easily segmented, a binary mask was created using the tubulin channel. This mask was transferred onto the GEF-H1 channel, and the ratio of the intensity of GEF-H1 within the mask (on microtubules) and outside the mask (outside microtubules) was calculated. Regions of dense microtubules were excluded from analysis.

#### Microtubule correlation

Protrusion regions were marked in live-cell imaging movies of endothelial cells with spyTubulin. First, microtubules were thresholded and segmented, and the correlation between each frame for the first five frames were calculated and averaged using a custom MATLAB script. To minimize variability across different experimental conditions and cell lines, the average correlation for each cell was normalised to the mean of the respective control cells in each experiment.

#### Microtubules at the cell edge

In control or FHL2 knockdown cells, a square region of a cell protrusion was marked, and microtubules were thresholded in the region. The area occupied by microtubules was measured.

#### Microtubule dynamics

Microtubules were labelled with spy-Tubulin and imaged with short time intervals (~5 s). Using Manual Tracking in Fiji, easily distinguishable, individual microtubules at the cell edge were tracked over the time course of the movie. One initial measurement of the microtubule lattice or shaft was marked to calculate the direction of growth or depolymerisation. Microtubule growth rate was defined by the average length increase per time for all frames during periods of growth. Depolymerisation was defined by average length decrease per time for all frames during periods of shrinkage. Speed variability is defined by the standard deviation of the absolute values of displacement between frames.

## Supplementary information

Peer Review File

Dataset EV1

Dataset EV2

Movie EV1

Movie EV2

Movie EV3

Expanded View Figures

## Data Availability

Raw files and counts from RNA sequencing data have been uploaded to Annotare (ArrayExpress E-MTAB-16095). Analysis codes are publicly available on GitHub (https://github.com/shailaja-seetharaman/Paper_draft_codes) and provided upon request. Plasmids will be deposited to Addgene. Data corresponding to the figures are made available on Zenodo (10.5281/zenodo.19020814). Any other raw data or unique reagents related to this study are available upon reasonable request. The source data of this paper are collected in the following database record: biostudies:S-SCDT-10_1038-S44318-026-00807-y.
