# Supplementary material for: Mechanosensitive FHL2 tunes endothelial function via microtubule-actomyosin crosstalk
Source: EMBO J. 2026 May 26;45(13):4569–604. doi: 10.1038/s44318-026-00807-y (PMC13324624; doi:10.1038/s44318-026-00807-y)
Supplement: Supplementary file 4 — Movie EV1 [file 44318_2026_807_MOESM4_ESM.zip › EV1 movie legend.docx]

**Extended View Movie:**

**Movie EV1. FHL2 knockdown alters microtubule stability in TeloHAECs.** Live-cell imaging showing microtubule dynamics in Control (Ctl, left) and FHL2 knockdown (shFHL2, right) TeloHAECs labeled with spyTubulin. We observed that the relative correlation of microtubules between frames in FHL2 knockdown cells is higher as compared to Control cells, suggesting that microtubules in FHL2 knockdown are less dynamic. Images were captured approximately 3 min (192s) over a period of approximately 1 h. Scale bar: 10 µm.
