# Supplementary material for: Mechanosensitive FHL2 tunes endothelial function via microtubule-actomyosin crosstalk
Source: EMBO J. 2026 May 26;45(13):4569–604. doi: 10.1038/s44318-026-00807-y (PMC13324624; doi:10.1038/s44318-026-00807-y)
Supplement: Supplementary file 5 — Movie EV2 [file 44318_2026_807_MOESM5_ESM.zip › EV2 movie legend.docx]

**Extended View Movie:**

**Movie EV2. FHL2 overexpression promotes microtubule dynamics in TeloHAECs.** Live-cell imaging showing microtubule dynamics in Control (Ctl, left) and FHL2 overexpression (FHL2 OE, right) TeloHAECs labeled with spyTubulin. We observed that the relative correlation of microtubules between frames is higher in FHL2 overexpression cells, suggesting a highly dynamic microtubule population in the presence of FHL2. Images were captured approximately 2 min (119 s) over a period of approximately 30 min. Scale bar: 10 µm.
