# Supplementary material for: Mechanosensitive FHL2 tunes endothelial function via microtubule-actomyosin crosstalk
Source: EMBO J. 2026 May 26;45(13):4569–604. doi: 10.1038/s44318-026-00807-y (PMC13324624; doi:10.1038/s44318-026-00807-y)
Supplement: Supplementary file 6 — Movie EV3 [file 44318_2026_807_MOESM6_ESM.zip › EV3 movie legend.docx]

**Extended View Movie:**

**Movie EV3:** FHL2-decorated actomyosin bundles imaged along with microtubules. Representative movie showing FHL2-mEmerald-expressing TeloHAECs (left in white/black, right in green) imaged along with microtubules (labelled in magenta, using spyTubulin). Dynamics of FHL2 and microtubules were captured every 120 s. Scale: 10 µm.
