# Supplementary material for: Mechanosensitive FHL2 tunes endothelial function via microtubule-actomyosin crosstalk
Source: EMBO J. 2026 May 26;45(13):4569–604. doi: 10.1038/s44318-026-00807-y (PMC13324624; doi:10.1038/s44318-026-00807-y)
Supplement: Supplementary file 7 — Expanded View Figures [file 44318_2026_807_MOESM7_ESM.pdf]

Expanded View Figures

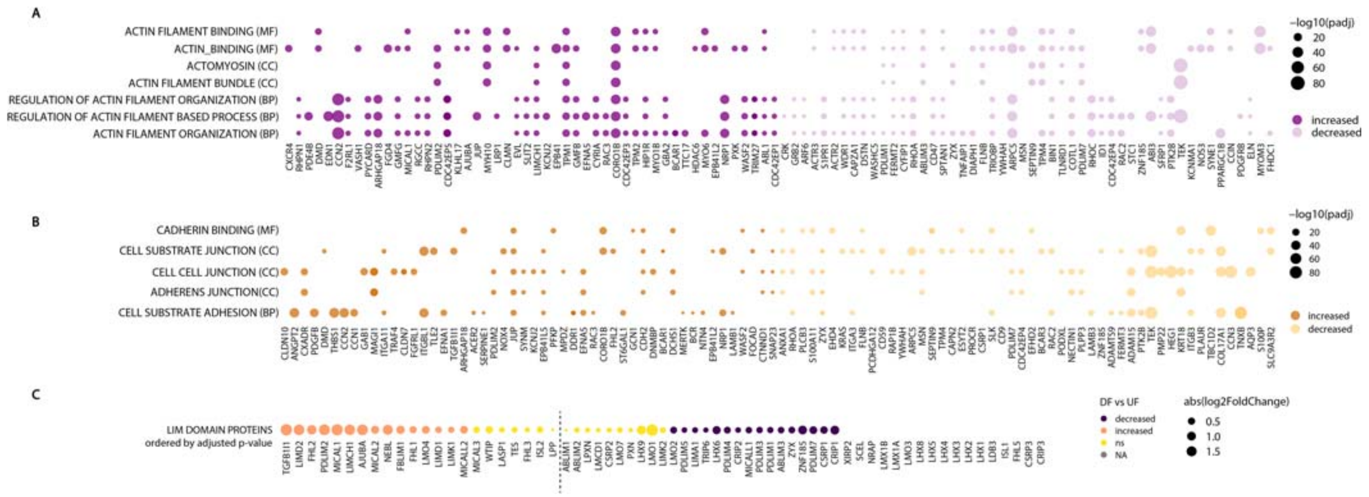

Figure EV1. Actin- and adhesion- related differentially expressed genes in response to flow.

(**A**) Actin-related genes present within the top 10 BP, CC or MF most enriched actin-related GO terms which are significantly different between endothelial cells under UF or DF are shown. The size of the dot reflects the $-\log_{10}$(adjusted *P* value). (**B**) Adhesion-related genes present within the top 10 BP, CC or MF most enriched adhesion-related GO terms which are significantly different between endothelial cells under UF or DF are shown. The size of the dot reflects the $-\log_{10}$(adjusted *P* value). (**C**) Dotplot showing all genes encoding for LIM domain proteins which were detected in endothelial cells under DF or UF. Genes are ordered by adjusted *P* value and $\log_2$FC. The size of the dots indicates absolute $\log_2$FC values. The left-most dot represents the most significantly upregulated gene in DF, and the right-most dot is the most significantly downregulated gene in DF encoding for a LIM domain protein (orange = *P*adj <0.05 & $\log_2$FC >0, purple = *P*adj <0.05 & $\log_2$FC <0, yellow = *P*adj = > 0.05). Genes of the LIM family that are not expressed in TeloHAECs are also shown.

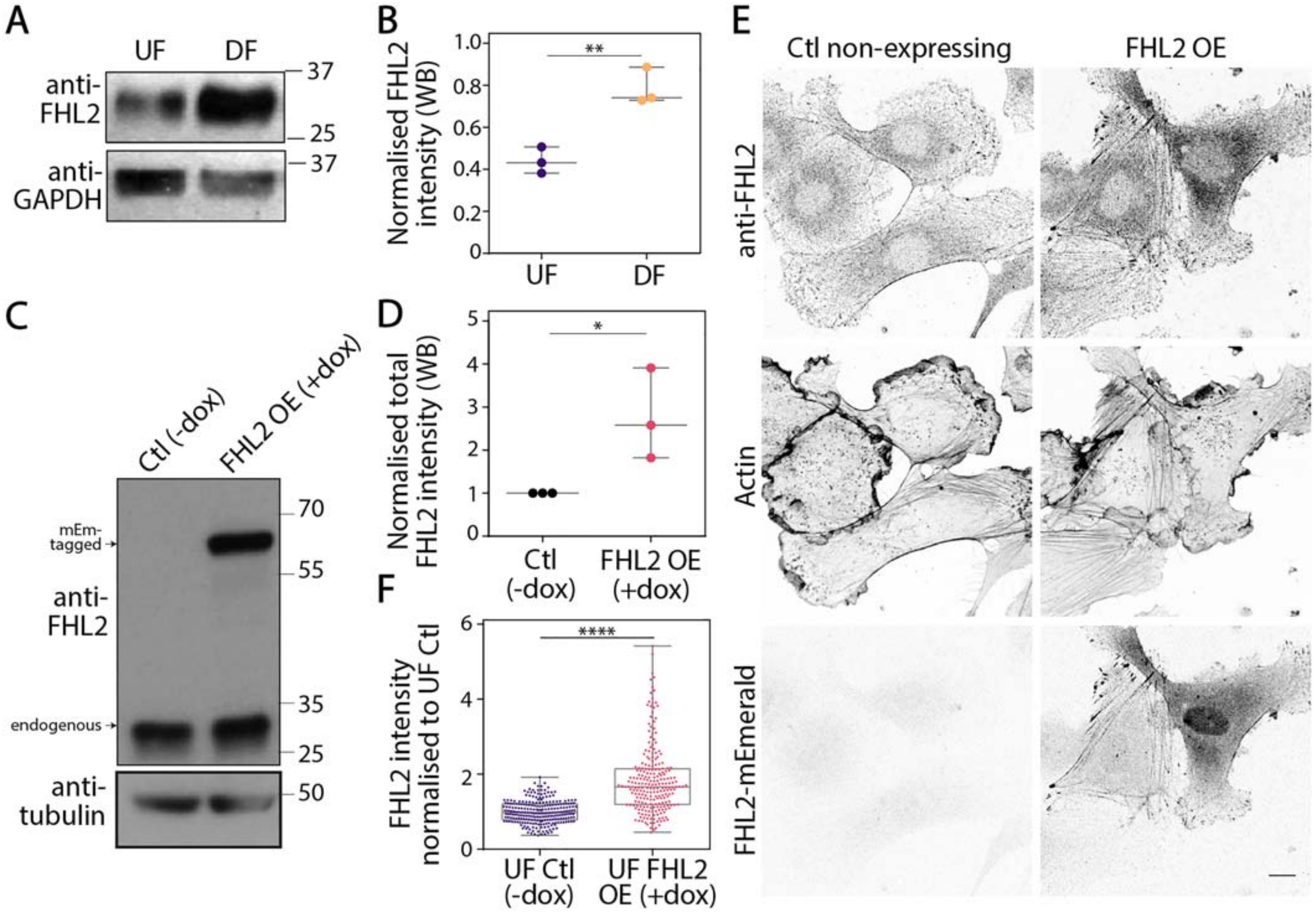

Figure EV2. FHL2 expression and localisation in endothelial cells.

(**A**) Western blot of lysates from TeloHAECs subjected to UF and DF for 24 h. Samples were analysed by immunoblotting for FHL2 and GAPDH (loading control). (**B**) Normalised FHL2 intensity in UF and DF cells. $N = 3$ independent experiments. Dots represent experimental means, whiskers show the minimum and maximum values, and the line within represents the median. (**C**) Western blot of lysates from Ctl (-dox) and FHL2-overexpressing (+dox; FHL2 OE) cells. Samples were analysed by immunoblotting for FHL2 and tubulin (loading control). FHL2 blotting reveals two bands - endogenous FHL2 (~32 kDa) and overexpressed m-Emerald tagged FHL2 protein (~60 kDa). (**D**) Graph shows the 'total FHL2 expression levels' in Ctl and FHL2 OE cells, defined by the sum of the intensities of both bands (FHL2 endogenous + overexpression) normalised to the respective loading control. $N = 3$ independent experiments. Dots represent experimental means, whiskers show the minimum and maximum values, and the line within represents the median. (**E**) Immunostaining of FHL2 and actin, and visualisation of mEmerald-FHL2 tagging in Ctl and FHL2-OE cells. (**F**) Ctl and FHL2 OE cells were subjected to UF and immunostained for FHL2 and actin. Graph shows normalised FHL2 intensity of each cell under UF in both conditions. $N = 2$ independent experiments ($n = 253$ for Ctl and 236 for FHL2 OE). Scale: (**E**) 10 μm. Statistical tests: (**B**, **D**) Unpaired *t* test. (**E**) Unpaired *t* test followed by Mann–Whitney test. $^{****}P < 0.0001$, $^{**}P < 0.01$, $^{*}P < 0.05$. In the box-and-whisker plots, the box extends from the 25th to 75th percentile, the whiskers show minimum and maximum values, and the line within the box represents the median.

 © The Author(s)

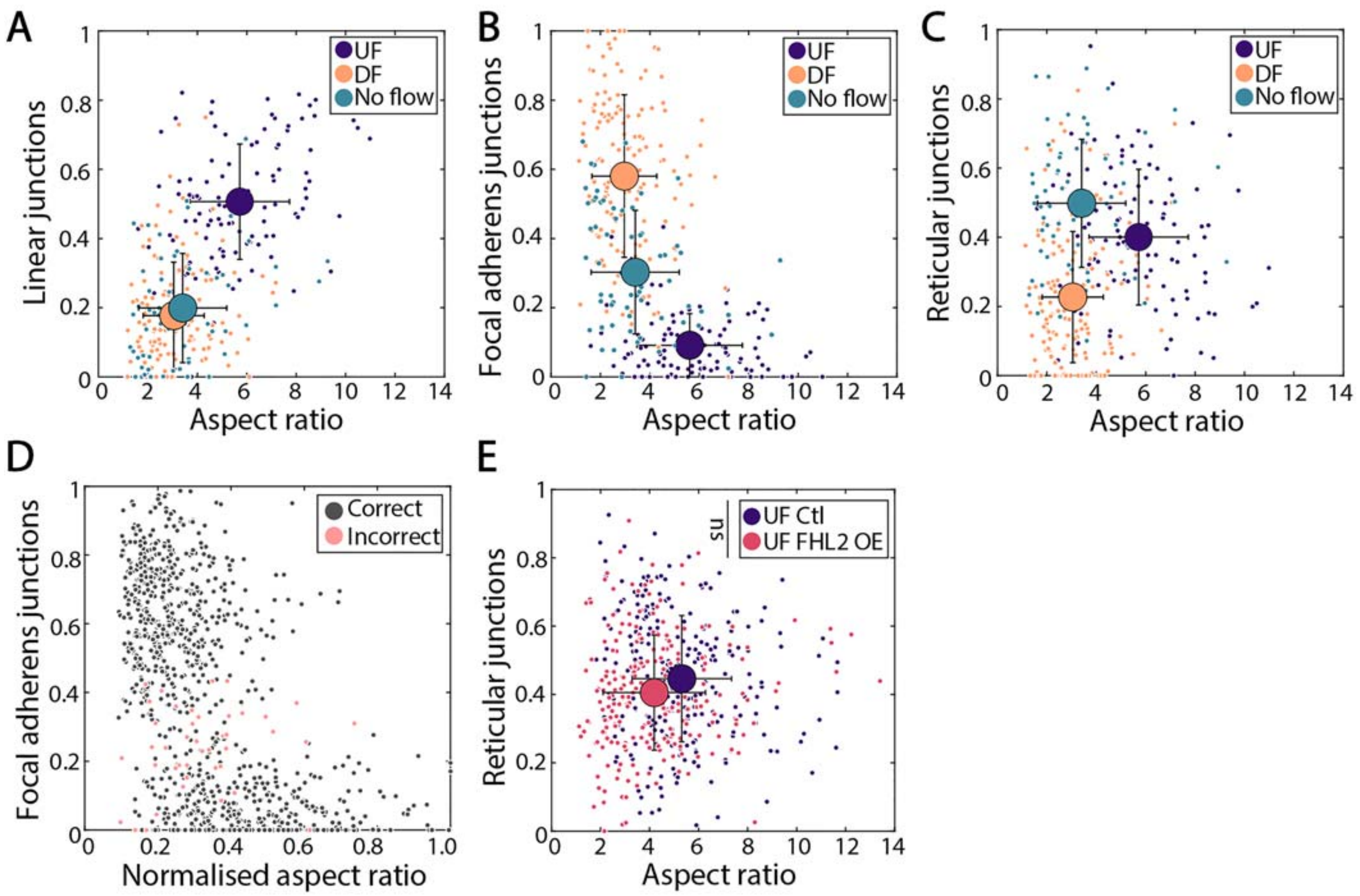

Figure EV3. Junction morphology analysis and unbiased clustering of cell populations under varying flow profiles.

(**A**) Graphs showing the aspect ratio (ratio of the major to the minor axis of a cell) vs. the fraction of the cell perimeter occupied by linear junctions (**A**), focal adherens junctions (**B**), reticular junctions (**C**). $n = 66$ for No Flow, 106 for UF, 149 for DF. (**D**) Using measurements of aspect ratio and fractions of the three junction types in Fig. 3B–D, cells were clustered using k-means, with $k = 2$. Two clusters and the corresponding data points are shown. $N > 3$ independent experiments ($n = 1179$ cells clustered into Cluster 1 or Cluster 2). Graphs showing the normalised aspect ratio vs. the fraction of the cell perimeter occupied by focal adherens junctions. Each cell is represented as a dot, and all the WT cells under UF and DF from across various experiments were clustered and are represented on the graph. Cells are colour coded based on whether they were correctly categorised into Clusters 1 or 2, based on the experimental condition. All the grey dots show that the cells that were under UF or DF were clustered into UF-like Cluster 1 or DF-like Cluster 2, respectively. The pink dots represent a mismatch in the clustering where the cells are clustered different from the actual experimental condition. (**E**) Graphs showing the aspect ratio (ratio of the major to the minor axis of a cell) vs. the fraction of the cell perimeter occupied by reticular junctions. $N = 2$ independent experiments ($n = 253$ for UF Ctl, 256 for UF FHL2 OE). Statistical tests: Unpaired *t* test. ns: *P* value not significant. In the box-and-whisker plots, the box extends from the 25th to 75th percentile, the whiskers show minimum and maximum values, and the line within the box represents the median.

© The Author(s)

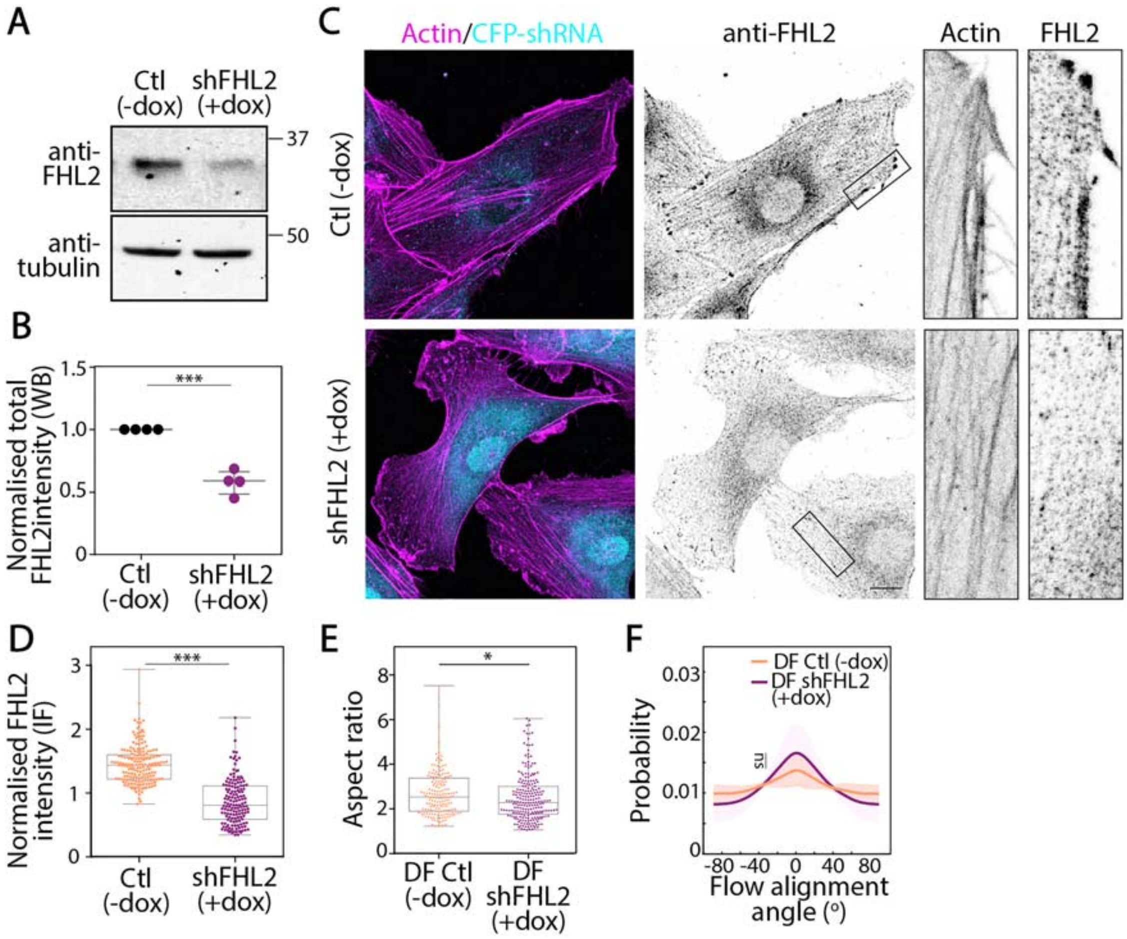

Figure EV4. Knockdown of FHL2 in endothelial tissue response to DF.

(**A**) Western blot of lysates from control (Ctl; -dox) and FHL2-knockdown (shFHL2; +dox) TeloHAECs. Samples were analysed by immunoblotting for FHL2 and tubulin (loading control). (**B**) Normalised FHL2 intensity in Ctl and shFHL2 cells. $N = 4$ independent experiments. Dots represent experimental means, whiskers show the minimum and maximum values, and the line within represents the median. (**C**) Inverted contrast images of Ctl and shFHL2 cells immunostained for actin and FHL2. (**D**) Fluorescence intensity of FHL2 normalised to actin intensity of each cell, in Ctl and shFHL2 TeloHAECs. $N = 5$ independent experiments ($n = 187$ for Ctl and 151 for shFHL2). (**E**) Graph shows the aspect ratio (ratio of the major to the minor axis of a cell) of control and shFHL2 (+dox) cells under DF. $N = 2$ independent experiments ($n = 171$ for Ctl, 225 for shFHL2). (**F**) From brightfield images, the alignment of Ctl and shFHL2 cells under DF are calculated by local gradient orientation and represented as the probability distribution of cells with the flow alignment angles (centred around 0°). Shaded error bars representing standard deviation are shown. $N = 1$ independent experiment ($n = 32$ for UF Ctl, and $n = 39$ for shFHL2; fields of view, with >100 cells/field). Scale: (C) 10 µm. Statistical tests: (**B**) Unpaired *t* test. (**D**) Paired, two-tailed *t* test. (**E**, **F**) Unpaired *t* test followed by Mann–Whitney test. $^*P < 0.05$, $^{**}P < 0.01$, $^{***}P < 0.001$, ns: *P* value not significant. In the box-and-whisker plots, the box extends from the 25th to 75th percentile, the whiskers show minimum and maximum values, and the line within the box represents the median.

 © The Author(s)

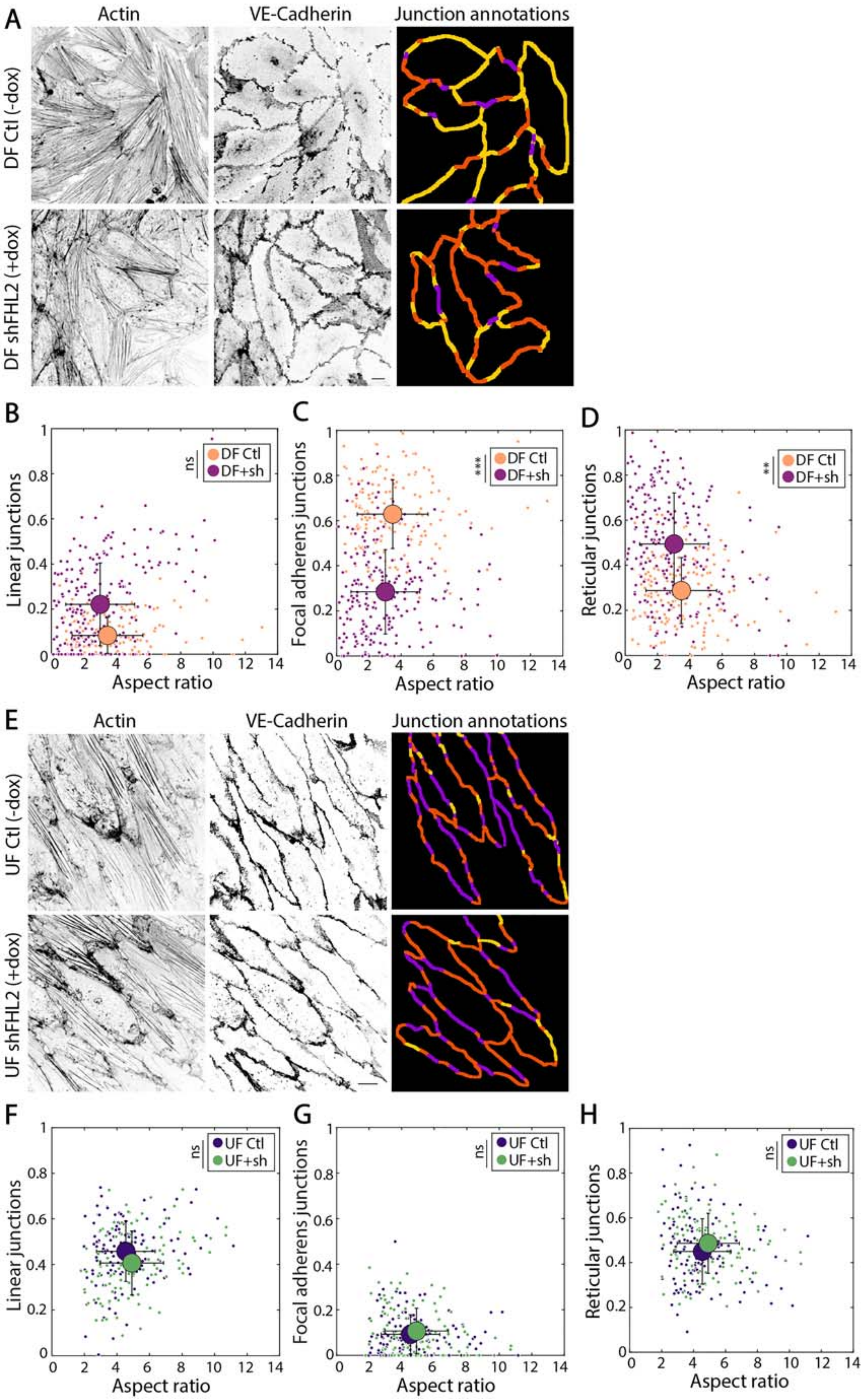

A
Actin
VE-Cadherin
Junction annotations
DF Ctl (-dox)
DF shFHL2 (+dox)
B
Linear junctions
Aspect ratio
ns
DF Ctl
DF+sh
C
Focal adherens junctions
Aspect ratio
***
DF Ctl
DF+sh
D
Reticular junctions
Aspect ratio
**
DF Ctl
DF+sh
E
Actin
VE-Cadherin
Junction annotations
UF Ctl (-dox)
UF shFHL2 (+dox)
F
Linear junctions
Aspect ratio
ns
UF Ctl
UF+sh
G
Focal adherens junctions
Aspect ratio
ns
UF Ctl
UF+sh
H
Reticular junctions
Aspect ratio
ns
UF Ctl
UF+sh

Figure EV5. Knockdown of FHL2 partially reverses focal adherens junction morphology without any effects on control conditions.

(**A**) Inverted contrast confocal images of control (Ctl; -dox) and shFHL2 TeloHAECs under DF, stained for phalloidin and VE-Cadherin. Annotated junctions are depicted as in Fig. 3A (purple: linear junctions, yellow: focal adherens junctions, orange: reticular junctions). (**B–D**) Graphs showing the aspect ratio (ratio of the major to the minor axis of a cell) vs. the fraction of the cell perimeter occupied by linear junctions (**B**), focal adherens junctions (**C**), reticular junctions (**D**). $N = 2$ independent experiments ($n = 171$ for DF Ctl, 225 for DF shFHL2). The DF Ctl means were pooled from 5 experiments for statistical tests. (**E**) Inverted contrast confocal images of control (Ctl; -dox) and shFHL2 TeloHAECs under UF, stained for phalloidin and VE-Cadherin. Annotated junctions are depicted as in Fig. 3A (purple: linear junctions, yellow: focal adherens junctions, orange: reticular junctions). (**F–H**) Graphs showing the aspect ratio (ratio of the major to the minor axis of a cell) vs. the fraction of the cell perimeter occupied by linear junctions (**F**), focal adherens junctions (**G**), reticular junctions (**H**). $N = 2$ independent experiments ($n = 135$ for UF Ctl, 107 for UF shFHL2). The UF Ctl means were pooled from five experiments for statistical tests. Scale: (**A**, **E**) 10 µm. Statistical tests: (**B–D**, **F–H**) Unpaired *t* test followed by Mann–Whitney test. $^{**}P < 0.01$, $^{***}P < 0.001$, ns: *P* value not significant.

 © The Author(s)

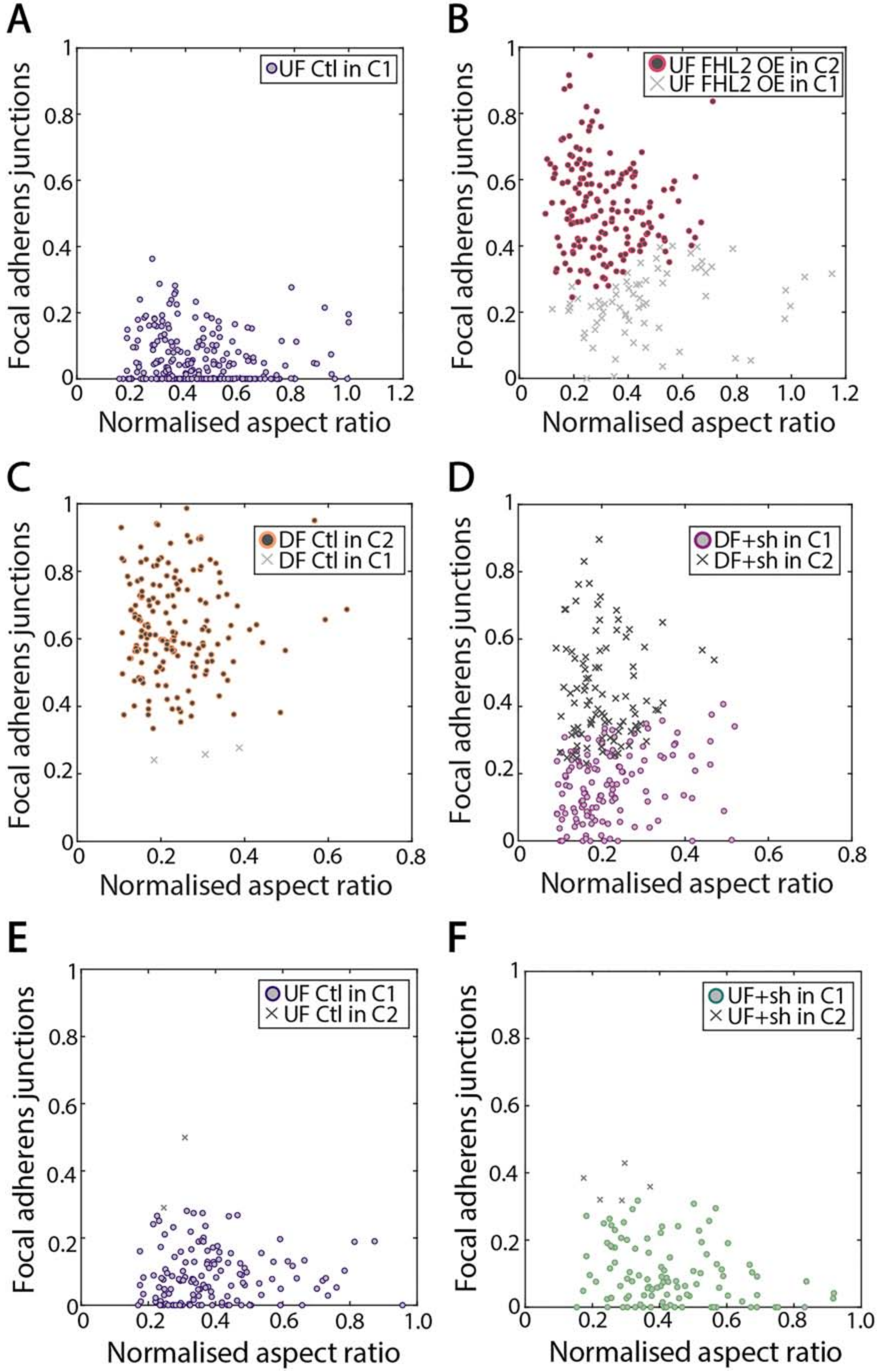
A
UF Ctl in C1
Focal adherens junctions
Normalised aspect ratio
B
UF FHL2 OE in C2
UF FHL2 OE in C1
Focal adherens junctions
Normalised aspect ratio
C
DF Ctl in C2
DF Ctl in C1
Focal adherens junctions
Normalised aspect ratio
D
DF+sh in C1
DF+sh in C2
Focal adherens junctions
Normalised aspect ratio
E
UF Ctl in C1
UF Ctl in C2
Focal adherens junctions
Normalised aspect ratio
F
UF+sh in C1
UF+sh in C2
Focal adherens junctions
Normalised aspect ratio

Figure EV6. Clustering analysis of cell populations in UF and DF revealed that FHL2 OE cells are highly represented in DF-like cluster.

(**A**, **B**) UF Ctl and FHL2 OE cells were clustered (unbiased) into the pre-defined UF-like Cluster 1 (C1) or DF-like Cluster 2 (C2). Graphs showing the normalised aspect ratio vs. the fraction of the cell perimeter occupied by focal adherens junctions in (**A**) Ctl, (**B**) FHL2 OE cells. (**A**) UF Ctl cells completely belong to UF-like Cluster 1. (**B**) FHL2 OE cells were clustered into the predefined clusters, and the circles represent the dominant cluster (in this case, DF-like Cluster 2; C2), while the ‘x’ depicts the minority cluster (in this case, UF-like Cluster 1; C1). (**C**, **D**) DF Ctl and shFHL2 cells were clustered (unbiased) into the pre-defined UF-like Cluster 1 (C1) or DF-like Cluster 2 (C2). Graphs showing the normalised aspect ratio vs. the fraction of the cell perimeter occupied by focal adherens junctions in (**C**) Ctl, (**D**) shFHL2 cells. (**C**) DF Ctl cells almost completely belong to DF-like Cluster 2 (circles represent the dominant cluster, in this case, DF-like Cluster 2, C2; while the ‘x’ depicts the minority cluster (in this case, UF-like Cluster 1; C1). (**D**) DF shFHL2 cells were clustered into the predefined clusters, and the circles represent the dominant cluster (in this case, UF-like Cluster 1; C1), while the ‘x’ depicts the minority cluster (in this case, DF-like Cluster 2; C2). (**E**, **F**) UF Ctl and shFHL2 cells were clustered (unbiased) into the pre-defined UF-like Cluster 1 (C1) or DF-like Cluster 2 (C2). Graphs showing the normalised aspect ratio vs. the fraction of the cell perimeter occupied by focal adherens junctions in (**E**) Ctl, (**F**) shFHL2 cells. (**E**) UF Ctl cells almost completely belong to UF-like Cluster 1 (circles represent the dominant cluster, in this case, UF-like Cluster 1, C1; while the ‘x’ depicts the minority cluster (in this case, DF-like Cluster 2; C2). (**F**) UF shFHL2 cells were clustered into the predefined clusters, and the circles represent the dominant cluster (in this case, UF-like Cluster 1; C1), while the ‘x’ depicts the minority cluster (in this case, DF-like Cluster 2; C2).

 © The Author(s)

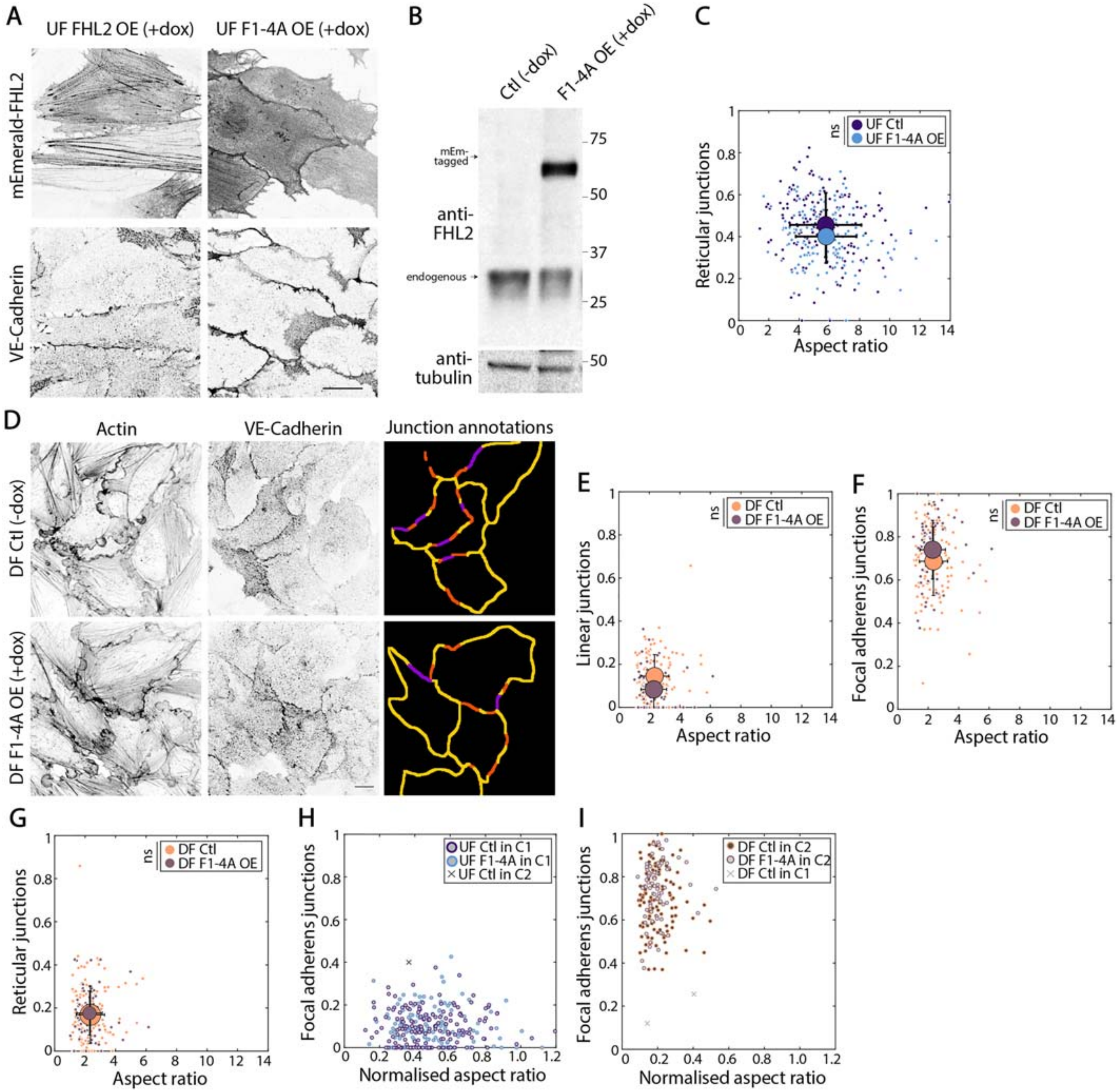
A
UF FHL2 OE (+dox)
UF F1-4A OE (+dox)
mEmerald-FHL2
VE-Cadherin
B
Ctl (-dox)
F1-4A OE (+dox)
mEm-tagged
anti-FHL2
endogenous
anti-tubulin
75
50
37
25
50
C
ns
UF Ctl
UF F1-4A OE
Reticular junctions
Aspect ratio
D
Actin
VE-Cadherin
Junction annotations
DF Ctl (-dox)
DF F1-4A OE (+dox)
E
ns
DF Ctl
DF F1-4A OE
Linear junctions
Aspect ratio
F
ns
DF Ctl
DF F1-4A OE
Focal adherens junctions
Aspect ratio
G
ns
DF Ctl
DF F1-4A OE
Reticular junctions
Aspect ratio
H
UF Ctl in C1
UF F1-4A in C1
UF Ctl in C2
Focal adherens junctions
Normalised aspect ratio
I
DF Ctl in C2
DF F1-4A in C2
DF Ctl in C1
Focal adherens junctions
Normalised aspect ratio

Figure EV7. FHL2 mutant and clustering analysis shows its similarity with UF-like Cluster 1.

(**A**) Inverted contrast images of FHL2-overexpressing (FHL2 OE; +dox) and FHL2-F1-4A-overexpressing (F1-4A OE; +dox) cells immunostained for VE-Cadherin. mEmerald tagging of FHL2 protein is visualised. (**B**) Western blot of lysates from control (Ctl; -dox) and F1-4A OE (+dox) cells. Samples were analysed by immunoblotting for FHL2 and tubulin (loading control). FHL2 blotting reveals two bands - endogenous FHL2 (~ 32 kDa) and overexpressed m-Emerald tagged FHL2-F1-4A mutant protein (~60 kDa). $N = 2$ independent experiments. (**C**) Graph showing the aspect ratio (ratio of the major to the minor axis of a cell) vs. the fraction of the cell perimeter occupied by reticular junctions. $N = 2$ independent experiments ($n = 143$ for UF Ctl, 167 for UF F1-4A OE). One data point belonging to UF Ctl (aspect ratio 18.25, linear 0.62, focal adherens 0.01) used in the analyses is excluded from the graph for representation purposes. The UF Ctl means were pooled from 5 experiments for statistical tests. (**D**) Inverted contrast confocal images of control (Ctl; -dox) and FHL2-F1-4A-overexpressing (F1-4A OE; +dox) TeloHAECs under DF, stained for phalloidin and VE-Cadherin. Annotated junctions are depicted as in Fig. 3A (purple: linear junctions, yellow: focal adherens junctions, orange: reticular junctions). (**E–G**) Graphs showing the aspect ratio (ratio of the major to the minor axis of a cell) vs. the fraction of the cell perimeter occupied by linear junctions (**E**), focal adherens junctions (**F**), reticular junctions (**G**). $N = 2$ independent experiments ($n = 121$ for DF Ctl, 74 for DF F1-4A OE). (**H**, **I**) UF Ctl and UF F1-4A OE cells were clustered (unbiased) into the pre-defined UF-like Cluster 1 (C1) or DF-like Cluster 2 (C2). Graphs showing the normalised aspect ratio vs. the fraction of the cell perimeter occupied by focal adherens junctions in (**H**) Ctl, (**I**) F1-4A OE cells. UF Ctl and UF F1-4A OE cells almost completely belong to UF-like Cluster 1 (circles represent the dominant cluster, in this case, UF-like Cluster 1, C1; while the 'x' depicts the minority cluster (in this case, DF-like Cluster 2; C2). Scale: (**A**, **D**) 20 µm. Statistical tests: (**C**, **E–G**) Unpaired *t* test followed by Mann–Whitney test. ns: *P* value not significant.

 © The Author(s)

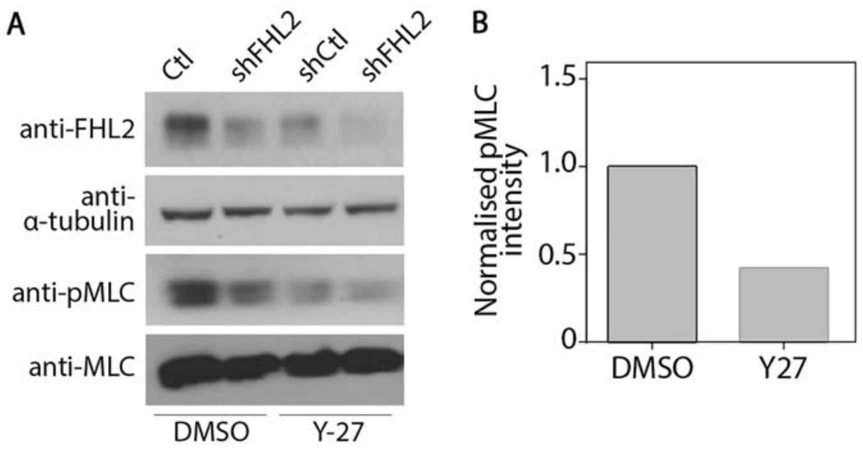

Figure EV8. FHL2 and pMLC levels upon ROCK inhibition using Y-27632.

(**A**) Western blot of lysates from Ctl and shFHL2 cells (from Fig. 5), with or without Rho-kinase inhibitor (Y-27632 a.k.a. Y-27) are shown. Samples were analysed by immunoblotting for FHL2, pMLC, MLC, and tubulin (loading control). For representative purposes, this blot blot shows the first two lanes that are also depicted in Fig. 5C. (**B**) Normalised pMLC intensity (pMLC/MLC intensity) in cells treated with DMSO Ctl or Y-27. $N = 1$ independent experiment. In the box-and-whisker plots, the box extends from the 25th to 75th percentile, the whiskers show minimum and maximum values, and the line within the box represents the median.

© The Author(s)

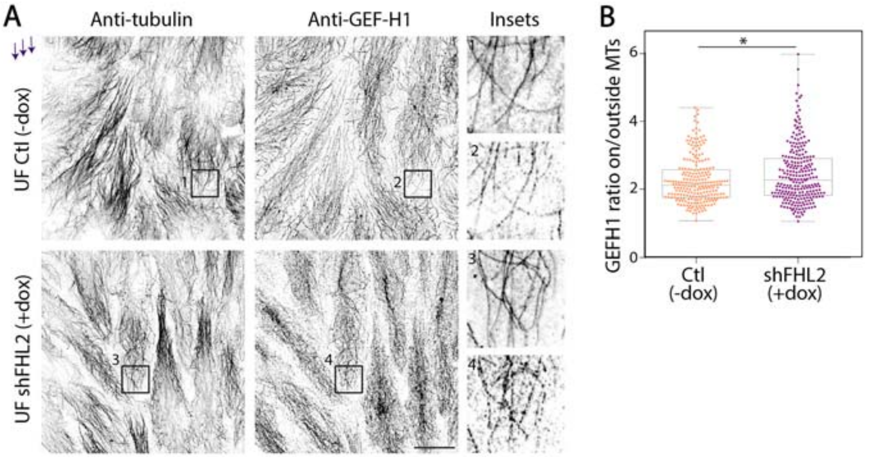

Figure EV9. GEFH1 localisation on microtubules in cells under UF.

(**A**) Inverted contrast confocal images of control (Ctl; -dox) and FHL2-knockdown (shFHL2; +dox) TeloHAECs under UF, immunostained for α-tubulin and Rho guanine nucleotide exchange factor, GEF-H1. (**B**) Graph showing the ratio of GEF-H1 intensity on vs. off microtubules. $N = 3$ independent experiments ($n = 237$ for UF Ctl, 250 for UF shFHL2; each dot represents a region of interest; ~1–2 regions per cell measured). Scale: (**A**) 20 µm. Statistical tests: (**B**) Unpaired *t* test followed by Mann–Whitney test. $^{*}P < 0.05$. In the box-and-whisker plots, the box extends from the 25th to 75th percentile, the whiskers show minimum and maximum values, and the line within the box represents the median.

 © The Author(s)
