# Supplementary material for: Mechanosensitive FHL2 tunes endothelial function via microtubule-actomyosin crosstalk
Source: EMBO J. 2026 May 26;45(13):4569–604. doi: 10.1038/s44318-026-00807-y (PMC13324624; doi:10.1038/s44318-026-00807-y)
Supplement: Supplementary file 1 — Peer Review File [file 44318_2026_807_MOESM1_ESM.pdf]

THE EMBO JOURNAL

Mechanosensitive FHL2 tunes endothelial function via microtubule-actomyosin crosstalk

Margaret Gardel, Shailaja Seetharaman, John Devany, Ha Kim, Emma van Bodegraven, Theresa Chmiel, Shentu Tzu-Pin, Wen-hung Chou, and Yun Fang

Corresponding author(s): Margaret Gardel (gardel@uchicago.edu) , Shailaja Seetharaman (shailaja@nus.edu.sg)

Review Timeline:

Submission Date:	5th Jul 24
Editorial Decision:	14th Aug 24
Revision Received:	14th Oct 25
Editorial Decision:	18th Dec 25
Revision Received:	22nd Mar 26
Accepted:	28th Apr 26

Editor: Ieva Gailite

Transaction Report:

(Note: With the exception of the correction of typographical or spelling errors that could be a source of ambiguity, letters and reports are not edited. Depending on transfer agreements, referee reports obtained elsewhere may or may not be included in this compilation. Referee reports are anonymous unless the Referee chooses to sign their reports.)

Dear Dr. Gardel,

Thank you for submitting your manuscript for consideration by the EMBO Journal. We have now received comments from three reviewers, which are included below for your information.

As you will see from the reports, all reviewers appreciate the topic and the quality of the work, while also pointing out a few overlapping concerns that would need to be addressed before they can recommend acceptance of the manuscript. As a common theme, these concerns focus on the physiological relevance of the data acquired using endothelial monolayers for the conditions in the blood vessels, as well as the need for a biochemical validation of the proposed changes in FHL2/actin and GEF-H1/microtubule interactions.

Based on overall interest expressed in the reviewer reports, I would invite you to revise the manuscript along the lines indicated in the referee comments, especially focusing on the points above. I think it would be helpful to discuss the revision in more detail via email or phone/videoconferencing - please let me know which option you prefer. I should also add that it is The EMBO Journal policy to allow only a single major round of revision and that it is therefore important to resolve the main concerns at this stage.

We generally allow three months as standard revision time, which can be extended to six months in the case of major revisions. Should you foresee a problem in meeting this deadline, please let us know in advance to discuss an extension.

As a matter of policy, competing manuscripts published during this period will not negatively impact on our assessment of the conceptual advance presented by your study. However, please contact me as soon as possible upon publication of any related work to discuss the appropriate course of action.

When preparing your letter of response to the referees' comments, please bear in mind that this will form part of the Review Process File and will therefore be available online to the community. For more details on our Transparent Editorial Process, please visit our website: https://www.embopress.org/page/journal/14602075/authorguide#transparentprocess. Please also see the attached instructions for further guidelines on preparation of the revised manuscript.

Please feel free to contact me if you have any further questions regarding the revision. Thank you for the opportunity to consider your work for publication. I look forward to discussing your revision.

With best wishes,

Ieva

Ieva Gailite, PhD
Senior Scientific Editor
The EMBO Journal
Meyerhofstrasse 1
D-69117 Heidelberg
Tel: +4962218891309
i.gailite@embojournal.org

Instructions for preparing your revised manuscript:

Please make sure you upload a letter of response to the referees' comments together with the revised manuscript.

Please also check that the title and abstract of the manuscript are brief, yet explicit, even to non-specialists.

When assembling figures, please refer to our figure preparation guideline in order to ensure proper formatting and readability in print as well as on screen:
https://bit.ly/EMBOPressFigurePreparationGuideline
See also guidelines for figure legends: https://www.embopress.org/page/journal/14602075/authorguide#figureformat

At EMBO Press we ask authors to provide source data for the main manuscript figures. Our source data coordinator will contact you to discuss which figure panels we would need source data for and will also provide you with helpful tips on how to upload and organize the files.

IMPORTANT: When you send the revision we will require
- a point-by-point response to the referees' comments, with a detailed description of the changes made (as a word file).
- a word file of the manuscript text.
- individual production quality figure files (one file per figure)
- a complete author checklist, which you can download from our author guidelines (https://www.embopress.org/page/journal/14602075/authorguide).
- Expanded View files (replacing Supplementary Information)
Please see out instructions to authors
https://www.embopress.org/page/journal/14602075/authorguide#expandedview
- a Reagents and Tools Table as part of the Methods section, which can be downloaded from our author guidelines (https://www.embopress.org/page/journal/14602075/authorguide#structuredmethods)

Please remember: Digital image enhancement is acceptable practice, as long as it accurately represents the original data and conforms to community standards. If a figure has been subjected to significant electronic manipulation, this must be noted in the figure legend or in the 'Materials and Methods' section. The editors reserve the right to request original versions of figures and the original images that were used to assemble the figure.

Further information is available in our Guide For Authors: https://www.embopress.org/page/journal/14602075/authorguide

We realize that it is difficult to revise to a specific deadline. In the interest of protecting the conceptual advance provided by the work, we recommend a revision within 3 months (12th Nov 2024). Please discuss the revision progress ahead of this time with the editor if you require more time to complete the revisions. Use the link below to submit your revision:

https://emboj.msubmit.net/cgi-bin/main.plex

Referee #1:

Through a series of in vivo (mice) and in vitro (human) experiments the Authors have shown that FHL2 expression levels in endothelial cells are coincident with their mechano-regulation. Specifically, in aortic endothelial cells, overexpression of FHL2 leads to hypercontractility, resulting in loss of barrier function due to rearrangements in junctional types - hallmarks of endothelial dysfunction. In addition to previous work that shows a number of other transcription factors implicated in disease progression, herein LIM domain proteins, including FHL2, are differentially regulated due to flow. While FHL2 binding to stress fibers is well-documented, here it is shown that binding to actin fibers leads to RhoA activation via GEF-H1 and subsequent cell contractility.
The results are interesting and well-presented; however, the main concern here is the use of endothelial monolayers to assess the majority of findings. It is well known that 2D versus 3D leads to differences in the response to flow (10.1016/j.biomaterials.2021.121248) and also results in variable morphology and permeability measurements (10.1016/j.biomaterials.2019.05.022) and transcription (particularly in the context of other non-endothelial subtypes 10.1039/D2LC00570K). Moreover, the response to flow in a distensible aorta with its layered structure may behave very differently to a single layer of endothelium. For instance, are the findings expected to be different on different matrices? what about the SMC component and different intimal layers? Moreover, heterogenous populations of endothelial cells exist in human aorta (https://doi.org/10.7554/eLife.91729.3) while here an immortalized line is used.

Increased contractility is proposed to be the consequence of discontinuous junctions. What is the upper and lower bounds of shear stress that results in deleterious junctional effects? (see 10.3389/fbioe.2019.00081)
Minor comments/suggestions
• Were any controls performed +dox? (PMC8351744) as doxycycline can impair mitochondrial function and can alter proliferation
• How was the timing of dox induction chosen? what is considered disturbed?
• It would help to highlight the expected range of shear for human aorta.
• Relative permeability is described, but should include molecular weight of solute.
• Fig. 2G - left is UF and right DF?
• Fig. 3I It is unclear why over-expression of FLH2 in uniform flow be similar to disturbed flow? Would it be important to show shFHL2 in UF conditions?
• Fig. 5 Traction force is higher in control than in knockdown FHL2, but under which condition? Possible to measure this after exposure to UF and DF?
• Authors mention tissue heterogeneity in FHL2 expression lines 366-68, but without any concrete examples. Please include in discussion.
• It would be nice to see whether deep learning approaches could identify differences in junctions and colocalizations between FHL2 and stress fibers.

Referee #2:

This manuscript by Seetharaman, et al, aims to connect the blood flow responsive gene, FHL2, to endothelial cell dysfunction under in vitro modeled athero-like conditions. Overall, the work is nicely done and the data are well presented. There are a few questions from this reviewer, before the manuscript would be considered suitable for publication.

Fig 2C should either be quantified or western blots carried out as secondary confirmation of changes in FHL2 protein levels

Supp. Fig. 2C, D-what is being quantified as the ctrl -dox condition? The WB doesn't seem to have signal in the control condition, so it seems that the increase following Dox exposure would be much higher than 2x.

Supp. Fig. 4G- panels are mislabeled

While the in vitro models are excellent as in vivo mimics, this reviewer would caution against calling the phenotypes athero-prone, given that athero will not develop in a dish.

Figure 4- What happens to the UF F1-4A OE under DF? Presumptively this is the more relevant flow type?

For panels such as Fig. 6A-F, while localization certainly looks co-associated, without super resolution microscopy or biochemical analysis of binding affinities/co-IPs, the data supports correlated localization, not direct binding. Same is true for discussions of FHL2 and actin binding. Either additional studies to confirm direct binding or softening of the language is needed.

For Fig. 6D,E what happens under UF?

Unless RhoA activity is assess, it should not be claimed to be regulated as an output of these studies.

Fig. 6G- how were these boundaries set? This data seems somewhat arbitrary, was this done under flow, how was a leading edge established?

Fig. 6H- how is the proposed restricted penetration of microtubules though actin stress fibers differentiated from the inability of microtubules to assemble/grow or their 'freezing' for other reason?

Images should be shown for all conditions quantified in Fig. 6I

Referee #3:

The study by Seetharaman and colleagues investigates the role of FHL2 (LIM-domain protein) in mechanosensitive responses of endothelial cells. Using transcriptional profiling of uniform vs. disturbed flow in vitro and in a carotid ligation mouse model, FHL2 is identified as up-regulated under disturbed flow. Gain- and loss-of-function links FHL2 to alignment and junction properties associated with disturbed flow, and constructs that prevent actin binding do not reproduce the effect. The study then moves to microtubules (MT), and evidence is presented that supports that changes in MT dynamics and regulation of the Rho activator GEF-H1 are linked to FMH2 manipulations. The final model that FHL2 regulates stress fibers that affect MT dynamics and GEF-H1 to affect contractility is consistent with the data and in line with other recently published work, and reveals novel findings regarding FHL2. Some minor details, if addressed, would strengthen the work.

Minor:

1. It would strengthen the work to include more MT analysis, for example plus-end tip tracking, to better understand how FHL2 affects the MT cytoskeleton.
2. Fig 6C is described twice in the text (p.8 line 296-301).
2. The insets of Fig 6D are very pixelated and do not well support the graph in 6E. Suggest higher resolution images.
4. Other work only briefly cited Aureille et al, 2024; Buglak et al, 2023) has examined peripheral MT/GEF-H1 interactions in focal adhesion regulation and endothelial adherens junctions contractility, suggest discuss this work in that context more thoroughly.

We thank all three reviewers for their thoughtful and constructive feedback. We are encouraged by their recognition of the significance and quality of our work, particularly the novelty of our findings on FHL2-mediated mechanotransduction in endothelial cells. The reviewers raised important points regarding the physiological relevance of our 2D in vitro models and the need for biochemical validation of FHL2/actin and GEF-H1/microtubule interactions. In response, we have incorporated stronger evidence from *in vivo* data, expanded our molecular analyses, clarified experimental conditions, and revised our terminology and figures for greater precision. We believe these revisions have strengthened the manuscript and addressed the reviewers' concerns comprehensively.

Referee #1: Through a series of in vivo (mice) and in vitro (human) experiments the Authors have shown that FHL2 expression levels in endothelial cells are coincident with their mechano-regulation. Specifically, in aortic endothelial cells, overexpression of FHL2 leads to hypercontractility, resulting in loss of barrier function due to rearrangements in junctional types - hallmarks of endothelial dysfunction. In addition to previous work that shows a number of other transcription factors implicated in disease progression, herein LIM domain proteins, including FHL2, are differentially regulated due to flow. While FHL2 binding to stress fibers is well-documented, here it is shown that binding to actin fibers leads to RhoA activation via GEF-H1 and subsequent cell contractility.
The results are interesting and well-presented; however, the main concern here is the use of endothelial monolayers to assess the majority of findings. It is well known that 2D versus 3D leads to differences in the response to flow (10.1016/j.biomaterials.2021.121248) and also results in variable morphology and permeability measurements (10.1016/j.biomaterials.2019.05.022) and transcription (particularly in the context of other non-endothelial subtypes 10.1039/D2LC00570K). Moreover, the response to flow in a distensible aorta with its layered structure may behave very differently to a single layer of endothelium. For instance, are the findings expected to be different on different matrices? what about the SMC component and different intimal layers? Moreover, heterogenous populations of endothelial cells exist in human aorta (https://doi.org/10.7554/eLife.91729.3) while here an immortalized line is used.

We thank the reviewer for their thoughtful comments and for highlighting the limitations of 2D in vitro models in capturing the full complexity of human vascular physiology. We agree that endothelial monolayers do not fully recapitulate the 3D architecture, cellular heterogeneity, and biomechanical environment of native vessels.

To address this concern, we validated our *in vitro* findings using an *in vivo* partial carotid ligation model, which induces disturbed flow in a physiologically relevant 3D vascular context. As shown in Fig. 2, FHL2 expression is significantly upregulated at both the transcriptional and protein levels in ligated arteries. Importantly, this *in vivo* model incorporates key features absent in 2D systems, including blood components, a layered structure comprising diverse cell types, and the presence of smooth muscle cells, fibroblasts, and extracellular matrix. Despite this complexity, we observed consistent upregulation of endothelial FHL2 in response to disturbed flow in the partially ligated carotid artery, reinforcing the robustness of our findings. In our qPCR experiments, we specifically isolated the intima—the endothelial lining of the artery—two days post-surgery, allowing us to assess acute flow-induced changes in FHL2 expression within a native vascular microenvironment. Notably, endothelial FHL2 expression changes occur prior to the formation of an atherosclerotic plaque in the artery, indicating that this gene expression occurs very early on and can play a role in endothelial dysfunction. This supports the physiological relevance of our focus on FHL2 gleaned from our in vitro studies. We do, however, recognise the limitations of the 2D cell culture models, and we have now highlighted the importance of our *in vivo* model and future areas to explore in the discussion (lines 457-460, 471-474).

To further support the relevance of our 2D model, we confirmed via RNA sequencing differential expression of several well-established atherosclerosis-associated endothelial markers, consistent with

patterns reported in *in vivo* and *in vitro* models (Fig. 1F). Additionally, to address the reviewers' concern, we made observations from published single-cell RNA sequencing data from human patient-derived (carotid endarterectomy) samples of the atherosclerotic core plaques and patient-matched proximal adjacent portions of the carotid artery tissue. Here, FHL2 expression was significantly enriched in the calcified atherosclerotic plaque core compared to adjacent healthy tissue (Alsaigh et al., *Communications Biology*, 2022; $p = 4.44 \times 10^{-5}$), where the atherosclerotic core represents a region characterized by disturbed flow and endothelial dysfunction. Similarly, in brain arteriovenous malformations, another context of disturbed flow in blood vessels, FHL2 was markedly upregulated compared to healthy tissue (Walchi et al., *Nature*, 2024; $p = 10^{-303}$). These observations support the role that FHL2 is upregulated in disturbed flow conditions in various scenarios.

We also acknowledge the reviewer's point regarding endothelial heterogeneity and the use of an immortalized cell line. While this is a limitation, our primary focus is on elucidating the mechanistic role of FHL2 in endothelial mechanotransduction. The majority of our findings (Figures 3-7) dissect the cellular pathways by which force-sensitive binding of FHL2 to the actin cytoskeleton promotes RhoA signalling via GEF-H1, leading to increased actomyosin contractility and aberrant cellular phenotypes. Although FHL2 has been implicated in mechanotransduction previously, this is the first detailed description of its downstream signalling mechanism alongside functional roles for FHL2-actin binding. Given that FHL2 is broadly expressed and interacts with conserved cytoskeletal components, we believe these mechanistic insights are likely generalisable across multiple cell types.

We hope this clarifies how our study integrates both *in vitro* and *in vivo* approaches to address the reviewer's concerns and supports the physiological relevance of our findings.

Increased contractility is proposed to be the consequence of discontinuous junctions. What is the upper and lower bounds of shear stress that results in deleterious junctional effects? (see 10.3389/fbioe.2019.00081).

We thank the reviewer for this insightful question. Previous studies have shown that endothelial junction integrity responds non-monotonically to shear stress. Specifically, both low shear stress (<2 dyn/cm²) and very high shear stress (>80 dyn/cm²) are associated with the formation of discontinuous junctions, whereas physiological arterial levels of laminar shear stress (approximately 10–30 dyn/cm²) support the maintenance of continuous, stable junctions (Caolo et al., 2018; Morel et al., 2021; Chandurkar et al., 2024).

In our study, we applied three distinct shear profiles: disturbed (~5 dyn/cm²), laminar (~20 dyn/cm²) and static/no flow. Consistent with the literature, we observed junctional disruption under both static (no flow) and DF conditions, while laminar UF preserved junctional integrity. In response to the reviewer, these comparisons are now included in Supplementary Fig. S3A-C and are discussed in the revised manuscript text (lines 194-199).

Minor comments/suggestions:

1) Were any controls performed +dox? (PMC8351744) as doxycycline can impair mitochondrial function and can alter proliferation

We thank the reviewer for raising this point. We have indeed taken this into consideration in our work. The concentration of doxycycline used in our experiments is as low as 0.1-0.2 µg/ml (as also cited by the review PMC8351744 and Zhou et. al., 2006). At these low concentrations, we found no significant differences in the rate of proliferation between -dox and +dox conditions over 6 days (Fig. 1 shown below). This is similar to Ahler et. al., 2013 where they show no effect at 0.1 µg/ml but impaired proliferation at 1 µg/ml or 5 µg/ml, which is 5 or 25 times the concentrations we use in our study (Ahler et. al., 2013; Moullan et. al., Cell Rep., 2015). Therefore, at the concentrations of dox as used in this paper, we believe that the off-target effects on other cellular processes in negligible.

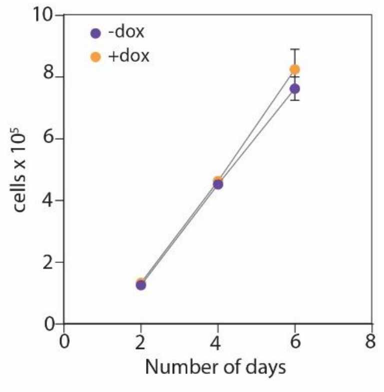

Figure 1. Cell numbers over 2, 4, or 6 days in control and doxycycline treated conditions.

2) How was the timing of dox induction chosen?

We thank the reviewer for raising this point, and we have now clarified this in the methods section. For the overexpression of FHL2, +dox treatment was carried out for a minimum of 24 h prior to experiment. This ensures significant expression of FHL2. On the other hand, for knockdown of FHL2, we performed a time course experiment of +dox treatment over 3-8 days. In all cases, FHL2 protein levels were determined by western blotting (Fig. 2 shown below). As we observed a significant drop in the levels of FHL2 ~3 days onwards in all conditions, we treated cells for at least 3-4 days with doxycycline prior to the experiment.

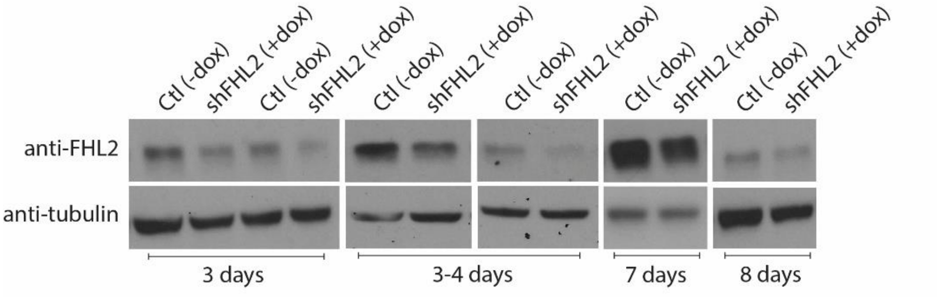

Figure 2. FHL2 knockdown levels at 3, 4, 7 and 8 days of doxycycline treatment.

3) what is considered disturbed?

We apologize for not clarifying this better. We have now defined disturbed flow and provided key references for this terminology (lines 60-70). The DF in atherosclerosis-prone regions of arteries is defined by the low magnitude shear stress with rapid changes in direction (Wang et. al., Arterioscler Thromb Vasc Biol, 2013, Yeh CF et. al., Sci Adv, 2022; Wu D et. al., eLife, 2017; Chiu and Chien, Physiol Rev, 2011). In our work, we are using established terminology for flow profiles derived from carotid artery bifurcations of normal human subjects (Dai et. al., PNAS, 2004). These flows were characterized using 3D flow analysis and consider actual vascular geometries and blood flow profiles. In these arteries, the authors describe two arterial waveforms (Fig. 3 below), "athero-prone" disturbed flow and "athero-protective" unidirectional flow, both of which distinctly represent regions of the artery that are susceptible or resistant respectively, to atherosclerotic plaque development. Based on this *in vivo* data, the disturbed flow DF profile consisted of spatial and time-dependent retrograde flow with an average shear stress of 2 dyn/cm^2. The UF and DF waveforms as described in the paper Dai et. al., PNAS, 2004 are shown below, and have been used in our study to model flow profiles experienced by endothelial cells.

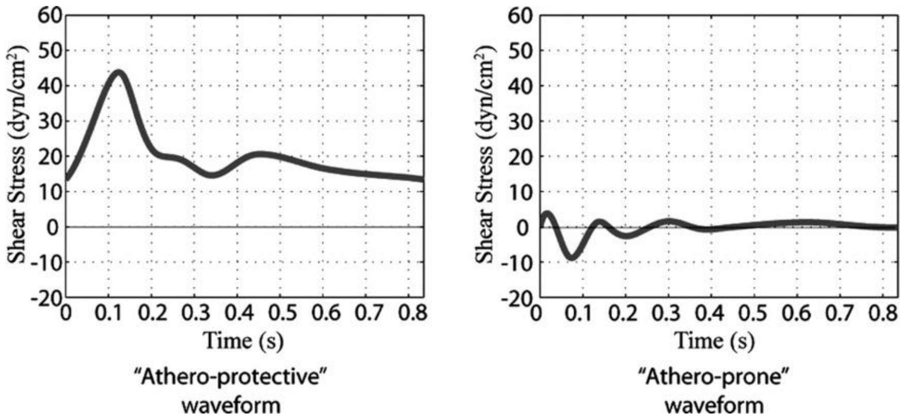

Figure 3. Athero-protective unidirectional flow and athero-prone disturbed flow waveforms from Dai et. al., PNAS, 2004.

4) It would help to highlight the expected range of shear for human aorta.

Thank you for this suggestion, we have now included these estimates in the first paragraph of the results (lines 62-66), describing the waveforms and expected range of shear with more clarity. We also provided citations to Dai et al. 2004 as well as other literature supporting these measurements.

5) Relative permeability is described but should include molecular weight of solute.

Thank you for this suggestion. We have now included the molecular weight of the dextran (4 kDa) in the methods section and Fig. 3.

6) Fig. 2G - left is UF and right DF?

Thank you for pointing this out, we have now labelled this on Fig. 2G.

7) Fig. 3I It is unclear why over-expression of FLH2 in uniform flow be similar to disturbed flow? Would it be important to show shFHL2 in UF conditions?

Thank you for this question as it pushed us to improve communication of key findings.

In short, we first found that FHL2 expression is increased in DF, and FHL2 localisation was enriched on actomyosin stress fibres (Fig. 2). This interesting observation led us to probe FHL2 functions further. Then, we tested if FHL2 expression is sufficient to drive athero-like phenotypes when we subjected cells to healthy UF conditions. This experimental design helped us uncover that the high expression of FHL2 and its localisation to actin was sufficient to drive endothelial dysfunction, even without any perturbations to healthy UF (Fig. 2).

We know that under UF conditions, FHL2 expression is already significantly low, so we hypothesized that lowering this expression further is not expected to affect junction morphology. We have now tested this hypothesis and observed no significant differences in junction morphologies between control and FHL2 knockdown cells under UF. The results have been added to Supplementary Fig. S5E-H.

Overall, in response to this question of the reviewer, FHL2 increases cellular contractility and causes discontinuous focal adherens junctions in endothelial monolayers, a similar phenotype observed in disturbed or static flow. Likewise, we find that FHL2 knockdown is sufficient to abrogate morphological changes observed in DF. We interpret these changes in transcriptional regulation of FHL2 to be downstream of flow sensing mechanotransduction pathways. The main finding of this current study was that FHL2 protein serves to stimulate actomyosin contractility and causes cell junction disruptions through its binding to mechanically strained actin filaments. Further, this discovery of positive feedback between actomyosin tension promoting FHL2 binding and, in turn, stimulating actomyosin tension provides insight to the mechanisms both by which actomyosin stresses are spatially distributed across adherent cells, and also how cells might tune the level of this stress.

8) Fig. 5 Traction force is higher in control than in knockdown FHL2, but under which condition? Possible to measure this after exposure to UF and DF?

The traction force measurements were carried out on endothelial cells which are under static/no flow conditions. In static conditions, FHL2 expression is high and knocking down FHL2 allowed us to assess how traction forces were altered. Unfortunately, the measurement of traction forces coupled to UF and DF was not possible in our hands due to the lack of live imaging capabilities along with the flow setup. It was also not possible for us to make PAA gels for traction force measurements inside microfluidic chips to measure forces during cell alignment, although this will be a very interesting future direction.

9) Authors mention tissue heterogeneity in FHL2 expression lines 366-68, but without any concrete examples. Please include in discussion.

Thank you for this suggestion. We have now cited expression levels and examples of different tissue types with variable FHL2 expression. These modifications are reflected in lines 428-432.

10) It would be nice to see whether deep learning approaches could identify differences in junctions and colocalizations between FHL2 and stress fibers.

We thank the reviewer for this excellent question, and this is also something that we are eagerly pursuing by building custom algorithms. Existing deep learning approaches including Cellpose and Ilastik have not been able to perform segmentation tasks accurately in these endothelial monolayers, particularly, in DF conditions where cell junctions are discontinuous. Therefore, we are currently developing deep learning algorithms to identify differences in junction morphologies and actin architecture. However, training these algorithms has been challenging due to various reasons including the need for more training data and validations, as well as optimization for the different junction morphologies. Unfortunately, this technique is not ready for use yet in this current manuscript.

Referee #2:

This manuscript by Seetharaman, et al, aims to connect the blood flow responsive gene, FHL2, to endothelial cell dysfunction under in vitro modeled athero-like conditions. Overall, the work is nicely done and the data are well presented. There are a few questions from this reviewer, before the manuscript would be considered suitable for publication.

1) Fig 2C should either be quantified or western blots carried out as secondary confirmation of changes in FHL2 protein levels.

We thank the reviewer for this comment and have now included quantification of the protein levels in the mouse sections in Figure 2C.

2) Supp. Fig. 2C, D-what is being quantified as the ctrl -dox condition? The WB doesn't seem to have signal in the control condition, so it seems that the increase following Dox exposure would be much higher than 2x.

We apologize for this confusion; this was indeed not explained well in the text. We have now clarified this point in the y axis label of the graph, marked the 2 bands on the blot, and added a description of the quantification in the methods section.

The western is blotted for anti-FHL2, which will mark all the FHL2 protein in cells, including the endogenous (~32 kDa) and overexpressed protein (~60 kDa). In the quantification, our goal was to measure the ‘total FHL2 protein levels’ in -dox and +dox conditions. The control -dox condition consists of only one band, which corresponds to the endogenous FHL2 protein levels, which is then normalized to the loading control (tubulin) intensity. On the other hand, for +dox condition, there are two bands visible – a low band marking endogenous FHL2 and a higher band (~60 kDa) marking the mEmerald-tagged-FHL2 overexpression. Here, we quantified the sum of the intensities of both bands (FHL2 endogenous +

overexpression) to determine the 'total FHL2 expression levels in +dox cells'. In other words, we are not quantifying the fraction/amount of overexpressed FHL2 protein with respect to the control, which would be much higher than 2x as pointed out by the reviewer, but we are merely measuring the 'total FHL2 protein expression' in the two conditions (which is endogenous + overexpressed protein in +dox).

3) Supp. Fig. 4G- panels are mislabeled

We apologize for this and have now corrected the labels for actin and VE-Cadherin.

4) While the in vitro models are excellent as in vivo mimics, this reviewer would caution against calling the phenotypes athero-prone, given that athero will not develop in a dish.

We agree with the reviewer and have revised our terminology throughout the manuscript. The reviewer is right to caution against calling the aberrant endothelial dysfunctional phenotypes as athero-prone. Therefore, we now refer to these phenotypes as athero-like (atherosclerosis-like) and have clearly defined this term in lines 61-70. We use the terminology "athero-prone" flow for disturbed flow, to be consistent with literature and to indicate the similarities in the waveforms (Dai et. al., Proc Natl Acad Sci USA, 2004; Wang et. al., Arterioscler Thromb Vasc Biol, 2013, Yeh CF et. al., Sci Adv, 2022; Wu D et. al., eLife, 2017; Chiu and Chien, Physiol Rev, 2011).

5) Figure 4- What happens to the UF F1-4A OE under DF? Presumptively this is the more relevant flow type?

By creating F1-4A mutant which lacks force-sensitive localization to stressed actin filaments, our goal was to explore whether perturbing this FHL2-binding to stress fibres is essential for its function in driving athero-like phenotypes. In UF, overexpressing FHL2 WT resulted in discontinuous focal adherens junction morphologies, and on the other hand, overexpressing mutant FHL2-F1-4A in UF did not result in discontinuous junctions (Fig. 4). This key result demonstrated that the binding of FHL2 to stressed actin filaments is necessary for promoting athero-like phenotypes, even when the flow is healthy UF.

However, the reviewer is right to point out an additional control experiment of UF F1-4A overexpression under DF. We have now added this experiment to show that overexpression of FHL2-F1-4A in DF did not have significant effects as compared to the control (Fig. S7D-G, S7I). This is as expected, because DF already leads to a high endogenous FHL2 expression and focal adherens junctions, and a further increase in mutant FHL2 does not have a significant effect.

6) For panels such as Fig. 6A-F, while localization certainly looks co-associated, without super resolution microscopy or biochemical analysis of binding affinities/co-IPs, the data supports correlated localization, not direct binding. Same is true for discussions of FHL2 and actin binding. Either additional studies to confirm direct binding or softening of the language is needed.

We appreciate this important question. We have revised our text to better clarify this and have softened the language used to describe our own data as the interactions between actin-FHL2 and GEF-H1-Microtubule have been demonstrated in several previous studies explained below.

The direct interaction of GEF-H1 with MTs has been previously documented in several studies (Krendel et. al., Nat Cell Biol. 2002; Ren et. al., J. Biol. Chem., 1998; Chang et. al., Mol. Biol. Cell, 2008). The localisation of GEF-H1, vis-à-vis its release from microtubules into the cytosol, is not only correlated with GEF-H1 protein activity but is also a critical mechanism for regulating cellular contractility (Rafiq et. al., Nat. Mat. 2019; Meiri et. al., Mol. Cell, 2012; Seetharaman et. al., Nat. Mat., 2022). Our results use analyses approaches established in prior work showing that the cytoplasmic versus microtubule-bound GEF-H1 is a clear readout of GEF-H1 activity. Our analysis revealed that FHL2 is an upstream regulator of GEF-H1 localisation, which informs its activity and ability to promote Rho signalling.

Similarly, the direct binding of FHL2 to stressed actin has been previously described through precise *in vitro* studies (Sun et. al., Dev Cell, 2020). Furthermore, studies have demonstrated the force-dependent localisation of FHL2 to actin (Sun et. al., Dev Cell, 2020; Nakazawa et. al., Proc. Nat. Acad. Sci. USA, 2016; Winkelman et. al., Proc. Nat. Acad. Sci. USA, 2020). In our work, we leverage this knowledge and the known FHL2 mutation F1-4A that blocks stress fibre binding (Sun et. al., Dev Cell, 2020) to understand the functional role of FHL2 and its localisation in endothelial mechanotransduction.

7) For Fig. 6D,E what happens under UF?

This is an excellent question which now helped us compare the baseline GEF-H1 release in UF and DF conditions, as well as the role of FHL2 in promoting GEF-H1 release. In revised Fig. S9, we show that FHL2 knockdown cells under UF have a slightly higher ratio of GEF-H1 on microtubules/cytoplasm as compared to the control cells (S9A, S9B). Further, when comparing control cells in UF (Fig. 6) and DF (Fig. S9), we find that GEF-H1 is predominantly sequestered on microtubules in UF conditions, in contrast to DF where it is seen released into the cytoplasm.

8) Unless RhoA activity is assess, it should not be claimed to be regulated as an output of these studies.

We thank the reviewer for raising this important point. We have now attempted RhoA activity pulldown experiments using ELISA (Cytoskeleton Inc. G-LISA kit). In short, by comparing active RhoA to total RhoA, we quantitatively measured the amount of RhoA activity in cells. Despite increasing the concentration of the lysate, it was extremely difficult to obtain detectable and accurate values of active RhoA in endothelial cells (<0.2, which is outside the range of 0.2-1 indicated in the kit). This is reflected also in the extremely low traction forces generated by endothelial cells (Fig. 5A, B) as compared to fibroblasts, which were used in the validation of the kit. Although we observed few experiments where the trends are what we expected, given the variability and detection limits, we are unable to draw solid conclusions from these experiments. Therefore, we did not include these experiments in the manuscript and have now softened the language to describe these results more accurately.

9) Fig. 6G- how were these boundaries set? This data seems somewhat arbitrary, was this done under flow, how was a leading edge established?

We have now added images to represent how the cell edge was determined and to support our results with better clarity. Here, the cell edge represents the cell periphery/boundary, and not a leading edge. These experiments were carried out on static, no flow conditions to be able to observe nice protrusive lamellipodia in well-spread cells for making reliable measurements. Given that our data on static conditions and DF are similar with respect to cell junctions and GEF-H1 release, and as seen in literature that static conditions represent a more DF phenotype (Noria et. al., Circ. Res., 1999; Oldenburg et. al., Sci. Rep., 2015), we believe that this condition is relevant for investigation.

10) Fig. 6H- how is the proposed restricted penetration of microtubules though actin stress fibers differentiated from the inability of microtubules to assemble/grow or their 'freezing' for other reason?

We thank the reviewer for this insightful question. The reviewer is correct in pointing out that the inability of microtubules to grow or their freezing might be due to other reasons. We apologize that we did not address this point clearly in the manuscript.

As seen in our measurements of the fraction of the cell edge occupied by microtubules, as well as the live imaging with actomyosin networks, we show that microtubules fail to penetrate and extend to the cell edge, with depolymerisation seen near the actomyosin bundles. This is in line with published literature, where in high-contractile situations with dense actomyosin bundles, these bundles act as a physical barrier that prevents the interaction of plus tips of microtubules with the membrane (Jiang et. al., Curr. Biol.

2012), or through actin retrograde flow causes rearward transport and depolymerisation of microtubules, restricting them from the periphery (Salmon et. al., J. Cell. Biol., 2002; Even-Ram et. al., Nat. Cell Biol., 2007; Dogterom and Koenderink, Nat. Rev. Mol. Cell Biol., 2018; Janson et. al., J. Cell. Biol. 2003; Waterman-Storer and Salmon, J. Cell Biol., 1997).

To delineate the mechanism further and address whether microtubules fail to penetrate due to their inability to grow or their freezing at the periphery, we now investigated how FHL2 affects microtubule dynamics. We demonstrate that FHL2 causes dynamic instability and fluctuations in the microtubule network, further supporting our findings from Fig. 7A-E. We measured microtubule dynamics near the cell periphery, specifically the growth and depolymerization or catastrophe rates. In the FHL2 knockdown cells, microtubules grow much slower and also depolymerise at a slower rate as compared to the control cells with FHL2 (Fig. 7F-I), suggesting that the microtubules are steadily venturing into the periphery and do not freeze or undergo depolymerisation as much. On the other hand, in control cells with FHL2-decorated actomyosin bundles, the microtubules grow and explore the protrusion at a faster rate; further, the microtubules also undergo rapid depolymerisation events, possibly due to actomyosin retrograde flow and tension, network crosslinking, or microtubule stabilization defects at the cortex (Seetharaman and Etienne-Manneville, J. Cell Sci., 2019; Bouchet et. al., eLife, 2016).

Thus, we actually propose that a combination of the above mechanisms occur in our system to tune cellular contractility, where: 1) FHL2-decorated actomyosin bundles in control cells restrict microtubules from penetrating into the periphery, and 2) FHL2 modulates microtubule dynamics, especially depolymerisation, which causes fewer microtubules to enter the protrusion.

In all of the above scenarios, our results are in line with other works showing that microtubule depolymerisation, restriction, and destabilisation leads to GEF-H1 release and heightened contractility (Rafiq et. al., Nat. Mat., 2019; Seetharaman et. al., Nat. Mat., 2022; Aureille et. al., EMBO Journal, 2024), as seen in FHL2 expressing cells. In the sections describing these results, we have now highlighted the possible mechanisms better by adding new results and modifying the language.

11) Images should be shown for all conditions quantified in Fig. 6I

We thank the reviewer for pointing this out, and we have now added representative images in the new Fig. 7C and 7D.

Referee #3: The study by Seetharaman and colleagues investigates the role of FHL2 (LIM-domain protein) in mechanosensitive responses of endothelial cells. Using transcriptional profiling of uniform vs. disturbed flow in vitro and in a carotid ligation mouse model, FHL2 is identified as up-regulated under disturbed flow. Gain- and loss-of-function links FHL2 to alignment and junction properties associated with disturbed flow, and constructs that prevent actin binding do not reproduce the effect. The study then moves to microtubules (MT), and evidence is presented that supports that changes in MT dynamics and regulation of the Rho activator GEF-H1 are linked to FMH2 manipulations. The final model that FHL2 regulates stress fibers that affect MT dynamics and GEF-H1 to affect contractility is consistent with the data and in line with other recently published work, and reveals novel findings regarding FHL2. Some minor details, if addressed, would strengthen the work.

Minor:

1) It would strengthen the work to include more MT analysis, for example plus-end tip tracking, to better understand how FHL2 affects the MT cytoskeleton.

We thank the reviewer for this suggestion. In the revised manuscript, we investigated how FHL2 affects microtubule dynamics. We demonstrate that FHL2 causes dynamic instability and fluctuations in the

microtubule network, further supporting our findings from Fig. 7A-7E. Using fast live-cell imaging of microtubules and tracking the ends, we measured microtubule dynamics near the cell periphery, specifically the growth and depolymerization or catastrophe rates. In the FHL2 knockdown cells, microtubules grow much slower and also depolymerise at a slower rate as compared to control cells expressing FHL2 (Fig. 7F-I), suggesting that the microtubules are steadily venturing into the periphery. On the other hand, in control cells with FHL2-decorated actomyosin bundles, the microtubules grow and explore the protrusion at a faster rate; further, the microtubules also undergo rapid depolymerisation events, possibly due to actomyosin retrograde flow, network crosslinking, or microtubule stabilization defects at the cortex (Seetharaman and Etienne-Manneville, J. Cell Sci., 2019; Bouchet et. al., eLife, 2016). This leads us to propose that in cells with FHL2 on stress fibres: 1) microtubules are restricted from penetrating into the periphery by the FHL2-decorated actomyosin networks, and 2) microtubule become highly dynamic and depolymerise with FHL2 expression. These results are in line with other works showing that microtubule depolymerisation, restriction, and destabilisation leads to GEF-H1 release and heightened contractility (Rafiq et. al., Nat. Mat., 2019; Seetharaman et. al., Nat. Mat., 2022; Aureille et. al., EMBO Journal, 2024; Meiri et. al., Mol. Cell, 2012), as seen in FHL2 expressing cells.

2) Fig 6C is described twice in the text (p.8 line 296-301).

We apologize for this mistake; the text is now modified.

3) The insets of Fig 6D are very pixelated and do not well support the graph in 6E. Suggest higher resolution images.

Thank you for this suggestion. We have replaced Fig. 6D with higher-resolution images to better support the quantification shown in Fig. 6E.

4) Other work only briefly cited Aureille et al, 2024; Buglak et al, 2023) has examined peripheral MT/GEF-H1 interactions in focal adhesion regulation and endothelial adherens junctions contractility, suggest discuss this work in that context more thoroughly.

We thank the reviewer and now have expanded discussion of this work (lines 352-357, 438-440, 449-455, 474-476).

Dear Margaret,

Thank you for submitting a revised version of your manuscript. I apologise for the delay in the assessment of your revised manuscript. I have received input from one of the original reviewers, who finds that their concerns have been sufficiently addressed. I have gone through your responses to the other two reviewers, and I find them constructive and reasonable. Therefore, there now remain only a few editorial points that need to be addressed before I can extend official acceptance of the manuscript:

1. Please correct the order and the headings of the manuscript sections to: Abstract / Keywords / Introduction / Results / Discussion / Methods / Data Availability / Acknowledgements / Disclosure and Competing Interests Statement / References / Figure Legends / Expanded View Figure Legends.
2. Please rename the supplementary Figures into Figure EV1 - EV9 and provide their legends after main figure legends.
3. CRediT has replaced the traditional author contributions section because it offers a systematic, machine-readable author contributions format that allows for more effective research assessment. Please remove the Authors Contributions from the manuscript and use the free text boxes beneath each contributing author's name in our online submission system to add specific details on the author's contribution. More information is available in our guide to authors.
4. Please rename "Declaration of interests" section into "Disclosure and competing interests statement".
5. Please update references according to The EMBO Journal style - where there are more than 10 authors on a paper, the first 10 should be listed, followed by 'et al.' Please remove DOIs from references to published articles.
6. Please rename the supplementary tables into "Dataset EV1" and "Dataset EV2" and add their legends to the respective files in a separate tab/worksheet.
7. Please rename the movie files "Movie EV1" - "Movie EV3" and zip each movie file together with its corresponding legend.
8. Figure panels for Fig 2N, Fig 7I,J, Fig S9A,B are not mentioned in the manuscript text; please add the corresponding callouts.
9. Please ensure that the movies are called out in the sequential numerical order.
10. Please provide source data for the main manuscript figures as requested by our source data analyst on August 22, 2024.
11. In our standard image integrity check, we noted that the figure panels appear to be reused between Figure 5C and Appendix Figure S8A (the first two lanes). If this is intentional, please note the reuse in the figure legend.
12. Please provide higher resolution images for Western blots, as these figure panels currently appear highly pixelated. This could be a pdf conversion issue.
13. Our data editors have flagged the following issues in figure legends that need correcting:
- Please provide the exact p values in the legends of figures 1C, D; 2B, D, F, G, K, L, M; 5B, D, F; 6C, E; 7B, E, G, H, I.
- Please indicate the statistical test used for data analysis in the legends of figures 1E, I; 2K.
- Please define the box plots in terms of minima, maxima, centre, bounds of box and whiskers, and percentile in the legends of figures 5B, F; 6C, E; 7B, E, G, H, I.
- Please provide information on the number and nature of replicates in the legends of figures 1E, 7I.
- Please define the error bars in the legends of figures 2B, G; 3B, C, D, G, H; 5D.
14. Papers published in The EMBO Journal are accompanied online by a 'Synopsis' to enhance discoverability of the manuscript. It consists of A) a short (1-2 sentences) summary of the findings and their significance, B) 3-4 bullet points highlighting key results and C) a synopsis image that is 550x300-600 pixels large (width x height, jpeg or png format). You can either show a model or key data in the synopsis image. Please note that the image size is rather small and that text needs to be readable at the final size.

Please let me know if you have any questions regarding any of these points. You can use the link below to upload the revised files.

Thank you again for giving us the chance to consider your manuscript for The EMBO Journal. I look forward to receiving the final version.

With best wishes,

Ieva

Ieva Gailite, PhD
Senior Scientific Editor
The EMBO Journal
Meyerhofstrasse 1
D-69117 Heidelberg
Tel: +4962218891309
i.gailite@embojournal.org

Instructions for preparing your revised manuscript:

Read our guidance for manuscript revisions and related editorial policies: https://link.springer.com/journal/44318/submission-guidelines#cms-Revised-submissions

We realize that it is difficult to revise to a specific deadline. In the interest of protecting the conceptual advance provided by the work, we recommend a revision within 3 months (18th Mar 2026). Please discuss the revision progress ahead of this time with the editor if you require more time to complete the revisions. Use the link below to submit your revision:

https://emboj.msubmit.net/cgi-bin/main.plex

Referee #2:

The authors have substantially addressed all of my concerns, and are thanked for the thorough efforts they put forward.

All editorial and formatting issues were resolved by the authors.

2nd Revision - Editorial Decision 28th Apr 2026

Dear Margaret and Shailaja,

Thank you for incorporating the final editorial and formatting requests in the manuscript. I am now pleased to inform you that your manuscript has been accepted for publication. Congratulations with a nice study!

Before we forward your manuscript to our publishers, there are a few of aspects that I still need your input on:

1. We need a statement about the whiskers/error bars in figure panels 2B, G; 5D to be added to the figure legends - do they represent the minimum and maximum values? I can add this information in the figure legend for you.

2. Please let me know if you would like to use British or American English spelling in your manuscript - currently, the verbs are written in British style, while the American English style is used when describing actin fibers (British English would be fibres).

3. We would like to propose some edits in the manuscript title, abstract and synopsis. We felt that the original title was a bit too general and wanted to add further information to catch the attention of our readers. I have also written a short blurb that will accompany the title of your manuscript in our online table of contents. Please take a look at the proposed text changes in the attached text file - any corrections or edits from your side are very welcome.

Once we have finalised these remaining questions, your manuscript will be processed for publication by EMBO Press. It will be copy edited and you will receive page proofs prior to publication. Please note that you will be contacted by Springer Nature Author Services to complete licensing and payment information.

You may qualify for financial assistance for your publication charges - either via a Springer Nature fully open access agreement or an EMBO initiative. Check your eligibility: https://link.springer.com/journal/44318/how-to-publish-with-us

Should you be planning a Press Release on your article, please get in contact with embo_production@springernature.com as early as possible in order to coordinate publication and release dates.

I look forward to hearing from you about the final changes soon, after which we will be able to swiftly proceed with the transfer of your manuscript for typesetting.

Best wishes,

Ieva

Ieva Gailite, PhD
Senior Scientific Editor
The EMBO Journal
Meyerhofstrasse 1
D-69117 Heidelberg
Tel: +4962218891309
i.gailite@embojournal.org

*****
Please note that it is The EMBO Journal policy for the transcript of the editorial process (containing referee reports and your response letters) to be published as an online supplement to each paper. If you should prefer removal of any referee-only figures included in the point-by-point response(s), e.g. because they may still be used for future publication or because they have been reproduced from published work by others, please do let us know immediately via response email.
More information is available here: https://link.springer.com/partners/embo-press/editorial-policies#Peer%20review

** Click here to be directed to your login page: https://emboj.msubmit.net

EMBO Press Author Checklist

Corresponding Author Name: Margaret Gardel, Shailaja Seetharaman
Journal Submitted to: The EMBO Journal
Manuscript Number: EMBOJ-2024-118397

USEFUL LINKS FOR COMPLETING THIS FORM
The EMBO Journal - Author Guidelines
EMBO Reports - Author Guidelines
Molecular Systems Biology - Author Guidelines
EMBO Molecular Medicine - Author Guidelines

Reporting Checklist for Life Science Articles (updated January
This checklist is adapted from Materials Design Analysis Reporting (MDAR) Checklist for Authors. MDAR establishes a minimum set of requirements in transparent reporting in the life sciences (see Statement of Task: 10.31222/osf.io/9sm4x). Please follow the journal's guidelines in preparing your
Please note that a copy of this checklist will be published alongside your article.

Abridged guidelines for figures
1. Data
The data shown in figures should satisfy the following conditions:
- the data were obtained and processed according to the field's best practice and are presented to reflect the results of the experiments in an accurate and unbiased manner.
- ideally, figure panels should include only measurements that are directly comparable to each other and obtained with the same assay.
- plots include clearly labeled error bars for independent experiments and sample sizes. Unless justified, error bars should not be shown for technical
- if n<5, the individual data points from each experiment should be plotted. Any statistical test employed should be justified.
- Source Data should be included to report the data underlying figures according to the guidelines set out in the authorship guidelines on Data

2. Captions
Each figure caption should contain the following information, for each panel where they are relevant:
- a specification of the experimental system investigated (eg cell line, species name).
- the assay(s) and method(s) used to carry out the reported observations and measurements.
- an explicit mention of the biological and chemical entity(ies) that are being measured.
- an explicit mention of the biological and chemical entity(ies) that are altered/varied/perturbed in a controlled manner.
- the exact sample size (n) for each experimental group/condition, given as a number, not a range;
- a description of the sample collection allowing the reader to understand whether the samples represent technical or biological replicates (including how many animals, litters, cultures, etc.).
- a statement of how many times the experiment shown was independently replicated in the laboratory.
- definitions of statistical methods and measures:
  - common tests, such as t-test (please specify whether paired vs. unpaired), simple χ2 tests, Wilcoxon and Mann-Whitney tests, can be unambiguously identified by name only, but more complex techniques should be described in the methods section;
  - are tests one-sided or two-sided?
  - are there adjustments for multiple comparisons?
  - exact statistical test results, e.g., P values = x but not P values < x;
  - definition of 'center values' as median or average;
  - definition of error bars as s.d. or s.e.m.

Please complete ALL of the questions below.
Select "Not Applicable" only when the requested information is not relevant for your study.

Materials

Newly Created Materials	Information included in the manuscript?	In which section is the information available? (Reagents and Tools Table, Materials and Methods, Figures, Data Availability Section)
New materials and reagents need to be available; do any restrictions apply?	Not Applicable	Data Availability

Antibodies	Information included in the manuscript?	In which section is the information available? (Reagents and Tools Table, Materials and Methods, Figures, Data Availability Section)
For **antibodies** provide the following information:<br>- Commercial antibodies: RRID (if possible) or supplier name, catalogue number and or/clone number<br>- Non-commercial: RRID or citation	Yes	Materials and Methods

DNA and RNA sequences	Information included in the manuscript?	In which section is the information available? (Reagents and Tools Table, Materials and Methods, Figures, Data Availability Section)
**Short novel DNA or RNA including primers, probes**: provide the sequences.	Yes	Materials and Methods

Cell materials	Information included in the manuscript?	In which section is the information available? (Reagents and Tools Table, Materials and Methods, Figures, Data Availability Section)
**Cell lines:** Provide species information, strain. Provide accession number in repository **OR** supplier name, catalog number, clone number, and/**OR** RRID.	Yes	Materials and Methods
**Primary cultures:** Provide species, strain, sex of origin, genetic modification status.	Not Applicable	
Report if the cell lines were recently **authenticated** (e.g., by STR profiling) and tested for mycoplasma contamination.	Yes	Materials and Methods

Experimental animals	Information included in the manuscript?	In which section is the information available? (Reagents and Tools Table, Materials and Methods, Figures, Data Availability Section)
**Laboratory animals or Model organisms:** Provide species, strain, sex, age, genetic modification status. Provide accession number in repository **OR** supplier name, catalog number, clone number, **OR** RRID.	Yes	Materials and Methods
**Animal observed in or captured from the field:** Provide species, sex, and age where possible.	Not Applicable	
Please detail **housing and husbandry conditions**.	Yes	Materials and Methods

Plants and microbes	Information included in the manuscript?	In which section is the information available? (Reagents and Tools Table, Materials and Methods, Figures, Data Availability Section)
**Plants:** provide species and strain, ecotype and cultivar where relevant, unique accession number if available, and source (including location for collected wild specimens).	Not Applicable	
**Microbes:** provide species and strain, unique accession number if available, and source.	Not Applicable	

Human research participants	Information included in the manuscript?	In which section is the information available? (Reagents and Tools Table, Materials and Methods, Figures, Data Availability Section)
If collected and within the bounds of privacy constraints report on age, sex and gender or ethnicity for all study participants.	Not Applicable	

Core facilities	Information included in the manuscript?	In which section is the information available? (Reagents and Tools Table, Materials and Methods, Figures, Data Availability Section)
If your work benefited from core facilities, was their service mentioned in the acknowledgments section?	Yes	Acknowledgements

Design

Study protocol	Information included in the manuscript?	In which section is the information available? (Reagents and Tools Table, Materials and Methods, Figures, Data Availability Section)
If study protocol has been **pre-registered, provide DOI in the manuscript**. For clinical trials, provide the trial registration number **OR** cite DOI.	Not Applicable	
Report the **clinical trial registration number** (at ClinicalTrials.gov or equivalent), where applicable.	Not Applicable	

Laboratory protocol	Information included in the manuscript?	In which section is the information available? (Reagents and Tools Table, Materials and Methods, Figures, Data Availability Section)
Provide DOI OR other citation details if **external detailed step-by-step protocols** are available.	Not Applicable	

Experimental study design and statistics	Information included in the manuscript?	In which section is the information available? (Reagents and Tools Table, Materials and Methods, Figures, Data Availability Section)
Include a statement about **sample size** estimate even if no statistical methods were used.	Yes	Figure legends
Were any steps taken to minimize the effects of subjective bias when allocating animals/samples to treatment (e.g. **randomization procedure**)? If yes, have they been described?	Yes	Materials and Methods
Include a statement about **blinding** even if no blinding was done.	Yes	Materials and Methods
Describe **inclusion/exclusion criteria** if samples or animals were excluded from the analysis. Were the criteria pre-established?<br><br>If sample or data points were omitted from analysis, report if this was due to attrition or intentional exclusion and provide justification.	Yes	Materials and Methods
For every figure, are **statistical tests** justified as appropriate? Do the data meet the assumptions of the tests (e.g., normal distribution)? Describe any methods used to assess it. Is there an estimate of variation within each group of data? Is the variance similar between the groups that are being statistically compared?	Yes	Materials and Methods

Sample definition and in-laboratory replication	Information included in the manuscript?	In which section is the information available? (Reagents and Tools Table, Materials and Methods, Figures, Data Availability Section)
In the figure legends: state number of times the experiment was **replicated** in laboratory.	Yes	Materials and Methods
In the figure legends: define whether data describe **technical or biological replicates**.	Yes	Materials and Methods

Ethics

Ethics	Information included in the manuscript?	In which section is the information available? (Reagents and Tools Table, Materials and Methods, Figures, Data Availability Section)
Studies involving **human participants**: State details of **authority granting ethics approval** (IRB or equivalent committee(s), provide reference number for approval.	Not Applicable	
Studies involving **human participants**: Include a statement confirming that **informed consent** was obtained from all subjects and that the experiments conformed to the principles set out in the WMA Declaration of Helsinki and the Department of Health and Human Services Belmont Report.	Not Applicable	
Studies involving **human participants:** For publication of **patient photos**, include a statement confirming that consent to publish was obtained.	Not Applicable	
Studies involving experimental **animals**: State details of **authority granting ethics approval** (IRB or equivalent committee(s), provide reference number for approval. Include a statement of compliance with ethical regulations.	Yes	Materials and Methods
Studies involving **specimen and field samples:** State if relevant **permits** obtained, provide details of authority approving study; if none were required, explain why.	Not Applicable	

Dual Use Research of Concern (DURC)	Information included in the manuscript?	In which section is the information available? (Reagents and Tools Table, Materials and Methods, Figures, Data Availability Section)
Could your study fall under dual use research restrictions? Please check biosecurity documents and list of **select agents and toxins** (CDC): https://www.selectagents.gov/sat/list.htm	Not Applicable	
If you used a select agent, is the security level of the lab appropriate and reported in the manuscript?	Not Applicable	
If a study is subject to dual use research of concern regulations, is the name of the **authority granting approval and reference number** for the regulatory approval provided in the manuscript?	Not Applicable	

Reporting

The MDAR framework recommends adoption of discipline-specific guidelines, established and endorsed through community initiatives. Journals have their own policy about requiring specific guidelines and recommendations to complement MDAR.

Adherence to community standards	Information included in the manuscript?	In which section is the information available? (Reagents and Tools Table, Materials and Methods, Figures, Data Availability Section)
State if relevant guidelines or checklists (e.g., **ICMJE, MIBBI, ARRIVE, PRISMA**) have been followed or provided.	Not Applicable	
For **tumor marker prognostic studies,** we recommend that you follow the **REMARK** reporting guidelines (see link list at top right). See author guidelines, under 'Reporting Guidelines'. Please confirm you have followed these guidelines.	Not Applicable	
For **phase II and III randomized controlled trials**, please refer to the **CONSORT** flow diagram (see link list at top right) and submit the CONSORT checklist (see link list at top right) with your submission. See author guidelines, under 'Reporting Guidelines'. Please confirm you have submitted this list.	Not Applicable	

Data Availability

Data availability	Information included in the manuscript?	In which section is the information available? (Reagents and Tools Table, Materials and Methods, Figures, Data Availability Section)
Have **primary datasets** been deposited according to the journal's guidelines (see 'Data Deposition' section) and the respective accession numbers provided in the Data Availability Section?	Yes	Will be made available in the Data Availability Section with Accession number
Were **human clinical and genomic datasets** deposited in a public access-controlled repository in accordance to ethical obligations to the patients and to the applicable consent agreement?	Yes	Data Availability Section with Accession number
Are **computational models** that are central and integral to a study available without restrictions in a machine-readable form? Were the relevant accession numbers or links provided?	Yes	Data Availability Section with GitHub link
If publicly available data were reused, provide the respective **data citations in the reference list**.	Not Applicable	
